# Borylation of α,β-Unsaturated Acceptors by Chitosan Composite Film Supported Copper Nanoparticles

**DOI:** 10.3390/nano8050326

**Published:** 2018-05-14

**Authors:** Wu Wen, Biao Han, Feng Yan, Liang Ding, Bojie Li, Liansheng Wang, Lei Zhu

**Affiliations:** 1 School of Chemistry and Materials Science, Hubei Engineering University, Hubei Collaborative Innovation Center of Conversion and Utilization for Biomass Resources, Xiaogan 432000, China; m17363363665@163.com (W.W.); hanbiao1125@163.com (B.H.); fengyanlab505@126.com (F.Y.); bojie.li@hbeu.edu.cn (B.L.); 2 School of Materials Science and Engineering, Hubei University, Wuhan 430062, China; 3 Department of Polymer and Composite Material, Yancheng Institute of Technology, School of Materials Engineering, Yancheng 224051, China

**Keywords:** chitosan composite film, copper nanoparticles, organoboron compounds, easily recyclable

## Abstract

We describe here the preparation of copper nanoparticles stabilized on a chitosan/poly (vinyl alcohol) composite film. This material could catalyze the borylation of α,β-unsaturated acceptors in aqueous media under mild conditions. The corresponding organoboron compounds as well as their converted β-hydroxyl products were all obtained in good to excellent yields. It is noteworthy that this catalyst of copper nanoparticles can be easily recycled eight times and remained catalytically reactive. This newly developed methodology provides an efficient and sustainable pathway for the synthesis of organoboron compounds and application of copper nanoparticles.

## 1. Introduction

**Figure 1:**
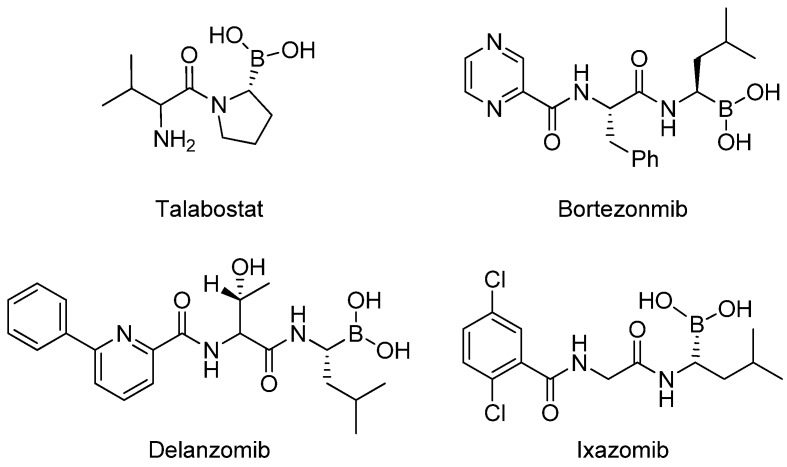
Organoboron compounds with biological activities.

Organoboron compounds attract an intensive interest in chemistry and pharmacy because of their versatile applications [1,2]. In recent years, these boron containing compounds were demonstrated with unique biological activities [3,4,5]. For example, talabostat [6] and bortezomib [7,8] were approved as proteasome inhibitors used for cancer treatment. Delanzomib [9] and ixazomib [10] were found to be active in the design of new drugs (Figure 1). On the other hand, organoboron derivatives are important intermediates for various chemical transformations [11,12,13,14,15], such as Suzuki–Miyaura coupling [16,17], Petasis reaction [18], allylborations [19], oxidations [20] and so on. Thus, new methodologies for the synthesis of organoboron compounds were continuously developed by chemists in both laboratories and industry. In past decades, series of methods have been reported for the establishment of C-B bonds. Among them, one of the most efficient pathway is the metal catalyzed borylation of α,β-unsaturated acceptors by using bis(pinacolato)diboron (B_2_(pin)_2_, **1**) [21,22]. The scope of metal catalysts was subsequently extended to Rh [23], Ni [24], Pt [25], Pd [26], Zn [27] and Cu [28,29,30,31,32,33,34,35]. Apparently, Cu-based catalytic system was most widely explored, owing to its low cost, less toxicity and solid performance over wide variety of substrates. However, several reported examples involved the use of strong base and specific designed ligands. Development of alternative highly active and sustainable copper catalyst for the borylation of α,β-unsaturated acceptors is still in high demand.

Metallic nanoparticles have been widely used as catalysts for many kinds of reactions [36,37,38,39,40]. To the best of our knowledge, only one example has shown that copper nanoparticles on carbon black were able to catalyze the borylation of α,β-unsaturated ketones [41]. However, further extension of the substrate scope was required, for example, α,β-unsaturated esters and amides. Besides, the use of organic solvent and deactivation of recycled catalyst largely limited the practical application of this method. In our previous work, we have found that chitosan supported copper could perform as a heterogeneous catalyst for the formation of C–B [42] and C–Si [43] bonds with 5 mol % catalyst loading. In both cases, either ligand or base must be added to avoid the copper leaching. Comparing with other supports, chitosan has inherent advantages due to its green property, abundance, stability and ability of chelation [44]. With our continuous efforts in exploring applications of chitosan supported metal catalysts, we were interested in developing a chitosan composite film stabilized copper nanoparticles and its application for the synthesis of useful organoboron compounds. Hence, we herein report copper nanoparticles anchored on a chitosan/poly (vinyl alcohol) (CP) composite film as highly reactive and recyclable catalysts for the boron conjugate additions of α,β-unsaturated acceptors.

## 2. Materials and Methods

### 2.1. Materials

Chitosan (degree of acetylation = 5%; *M*_v_ 80 kDa) was purchased from Aladdin (Shanghai, China), poly (vinyl alcohol) (*M*_w_ 120 kDa) was purchased from Sinopharm Chemical Reagent Co. Ltd., (Beijing, China), bis (pinacolato) diboron (CAS: 73183-34-3) was purchased from Energy Chemical (Shanghai, China) and Cu(II) salts were purchased from J&K (Beijing, China). All α,β-unsaturated acceptors were obtained commercially from Energy Chemical (Shanghai, China) and used without further purification. Chitosan/poly (vinyl alcohol) composite film supported copper nanoparticles (CP@Cu NPs) was prepared according to the procedures reported [45]. As shown in Supplementary Materials (Figures S1 and S2), the IR spectra and SEM image of the prepared CP@Cu NPs were similar with literature report [45].

### 2.2. Analytical Methods

Nuclear magnetic resonance (NMR) spectra were recorded on a Bruker Avance III 400 MHz spectrometer (Karlsruhe, Germany), operating at 400 for ^1^H and 100 MHz for ^13^C NMR in CDCl_3_ unless otherwise noted. CDCl_3_ is served as the internal standard (δ = 7.26 ppm) for ^1^H NMR and (δ = 77.0 ppm) for ^13^C NMR. Data for ^1^H-NMR is reported as follows: chemical shift (ppm, scale), multiplicity (s = singlet, d = doublet, t = triplet, q = quartet, m = multiplet and/or multiplet resonances, br = broad), coupling constant (Hz), and integration. Data for ^13^C NMR are reported in terms of chemical shift (ppm, scale), multiplicity, and coupling constant (Hz). Mass spectra were recorded on an Agilent 5975C (Santa Clara, CA, USA) using electrospray ionization (ESI) techniques. Infrared (IR) spectra were obtained using a Nicolet 380 spectrometer (Waltham, MA, USA). Scanning electron microscope (SEM) image was recorded on a Hitachi X-650 scanning electron microscope (Tokyo, Japan). Purification of products was accomplished using flash column chromatography on silica gel (200–300 mesh) or preparative TLC. The weight percentage and metal leaching of copper were determined by inductively coupled plasma-optical emission spectroscopy (ICP-OES) (PerkinElmer, Waltham, MA, USA) analysis. The copper loading of CP@Cu NPs was found to be 0.37 mmol/g.

### 2.3. General Procedure for the Sample Preparation for ICP Analysis to Determine Catalyst Loading

Chitosan/poly (vinyl alcohol) composite film supported copper nanoparticles (CP@Cu NPs) (~20 mg) was placed in a clean test tube and heated with H_2_SO_4_ (1 mL) at 200 °C. After 30 min, several drops of concentrated HNO_3_ were added carefully and the tube was shaken occasionally. HNO_3_ was continuously added until a clear solution was obtained and excess amount of HNO_3_ was allowed to evaporate under heating. After the solution was cooled to room temperature, 1 mL of aqua regia was added carefully. Effervescence of gas was observed and the solution become clearer. The solution was then transferred to a volumetric flask and made up to 50 mL with water which was submitted for ICP analysis.

### 2.4. General Procedure for the Sample Preparation for ICP Analysis to Determine Metal Leaching

After the reaction was finished, the reaction mixture was filtered. The filtrate obtained was concentrated and diluted with 10 mL of THF. Then, 50% *v*/*v* of the crude THF solution (5 mL) was then passed through a membrane filter (0.25 or 0.45 μm) into a clean test tube. After evaporation of solvent, the solid obtained in the test tube was heated to 200 °C and 1.0 mL of concentrated H_2_SO_4_ was added. Following a procedure similar to that described above, concentrated HNO_3_ were added at regular intervals until the resulting solution was clear. After the solution was cooled to room temperature, 1 mL of aqua regia was added carefully. Effervescence of gas was observed and the solution become clearer. The solution was then transferred to a volumetric flask and made up to 50 mL with water which was submitted for ICP analysis.

### 2.5. General Procedure for CP@Cu NPs-Catalyzed Borylation of α,β-Unsaturated Acceptors in Aqueous Media

In a sample vial, acetone (1.6 mL) and H_2_O (0.4 mL) were successively added to a mixture of CP@Cu NPs (5.4 mg, 1 mol %) and B_2_(pin)_2_ (**1**) (60.9 mg, 0.24 mmol) under air. The reaction mixture was stirred for 1 h at room temperature, followed by successive addition of substrate (**2**) (0.2 mmol) After stirring for 12 h at room temperature, the reaction mixture was filtered and the filtrate was extracted with EtOAc (20 mL) three times. The combined organic phase was concentrated under reduced pressure and the residue was dissolved in THF (3 mL) and H_2_O (2 mL). Excess amount of NaBO_3_·4H_2_O (244 mg) was then added and the mixture was stirred at room temperature for 4 h. The aqueous layer was extracted with EtOAc (20 mL) three times, and the combined organic layers were dried over anhydrous Na_2_SO_4_. After concentrated under reduced pressure, the crude mixture was purified by preparative TLC (petroleum ether/EtOAc = 4/1~10/1) to afford the desired product **3**.

### 2.6. General Procedure for Gram-Scale Synthesis of **3a**

In a 100 mL round bottom flask, acetone (48 mL) and H_2_O (12 mL) were successively added to a mixture of CP@Cu NPs (162 mg, 1 mol %) and B_2_(pin)_2_ (**1**) (1.83 g, 7.2 mmol) under air. The reaction mixture was stirred for 1 h at room temperature, followed by successive addition of chalcone (**2a**) (1.25 g, 6 mmol) After stirring for 12 h at room temperature, the reaction mixture was filtered and the filtrate was extracted with EtOAc (50 mL) three times. The combined organic phase was concentrated under reduced pressure and the residue was dissolved in THF (30 mL) and H_2_O (20 mL). Excess amount of NaBO_3_·4H_2_O (7.32 g) was then added and the mixture was stirred at room temperature for 6 h. The aqueous layer was extracted with EtOAc (50 mL) three times, and the combined organic layers were dried over anhydrous Na_2_SO_4_. After concentrated under reduced pressure, the crude mixture was purified by flash column chromotograph (petroleum ether/EtOAc = 4/1) to afford the desired product **3a** in 92% yield (1.25 g) as a white solid.

### 2.7. Recycling and Reuse of CP@Cu NPs

To demonstrate the recyclability of CP@Cu NPs, the boron conjugate addition was repeated eight times with the same composite film. The initial amount of catalyst was 54 mg (1 mol % Cu loading). Reactions were carried out for 12 h. After the reaction, catalyst was filtered off, washed by EtOAc and water, and then dried for 4 h at 50 °C before next run. Each recovery rate of the catalyst at the time of reusing is about 90%.

## 3. Results and Discussion

For initial investigation, we conducted the reaction of bis(pinacolato)diboron **1** with chalcone **2a** by using the prepared chitosan/poly (vinyl alcohol) composite film supported copper nanoparticles (CP@Cu NPs) as catalyst. To enhance the applicability of our designed strategy, one-pot process of borylation followed by oxidation was chosen as the model reaction (Table 1). First, various organic solvents were screened, however, reaction in toluene, diethyl ether and tetrahydrofuran did not proceed at all (Table 1, Entries 1–3). It was reported alcohol additive can accelerate the catalytic cycle [28] because protonation process with alcohol was recognized as the rate determine step [46]. Thus, we used methanol as an additive and the desired product **3a** was successfully observed (Table 1, Entries 4–6). To our delight, reaction in acetone with one equivalent methanol as additive gave product in 66% yield (Table 1 Entry 7). Besides, comparable result was obtained when only methanol was used as the solvent (Table 1, Entry 8). Based on our previous explorations, organoboron [42] and organosilicon [43,47] compounds could be formed in water under mild conditions. Unlike traditional organic solvents, water is non-toxic, cheap, safe and non-flammable. Thus, we turned to involve water as a co-solvent and ran the reaction in aqueous media. Apparent improvements were found when aqueous media was used instead of pure organic solvents (Table 1, Entries 9–13) and acetone/H_2_O mixture was the best one. After screening different ratios of acetone to water, the solvent was finally determined as a 4:1 (*v*/*v*) mixture (Table 1, Entry 12). In absence of catalyst, the reaction did not proceed at all (Table 1, Entry 14). By changing support to either chitosan or chitosan/poly (ethylene glycol) composite film, the reactivities diminished significantly (Table 1, Entries 15 and 16). These results indicated that the CP composite film is essential to stabilize and activate the copper nanoparticles. To be mentioned, the copper nanoparticles were extremely active for borylation of chalcone comparing with previous reports [41,42]. The copper loading for this reaction is as low as only 1 mol%. It is also feasible to further reduce the catalyst loading to half while a similar result was achieved (Table 1, Entry 17). In addition, argon atmosphere was not necessary in our procedures (Table 1, Entry 18). Therefore, the optimized reaction conditions were determined to run the reaction at room temperature under air in acetone/H_2_O (*v*/*v* = 4/1), using CP@Cu NPs as catalyst (Table 1, Entry 12).

**Figure 2:**
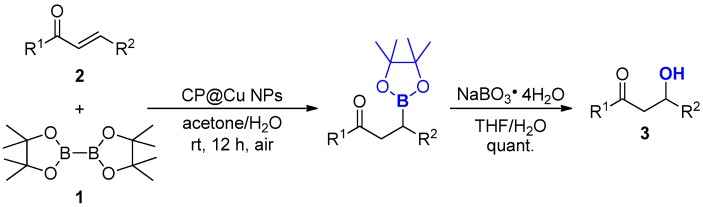

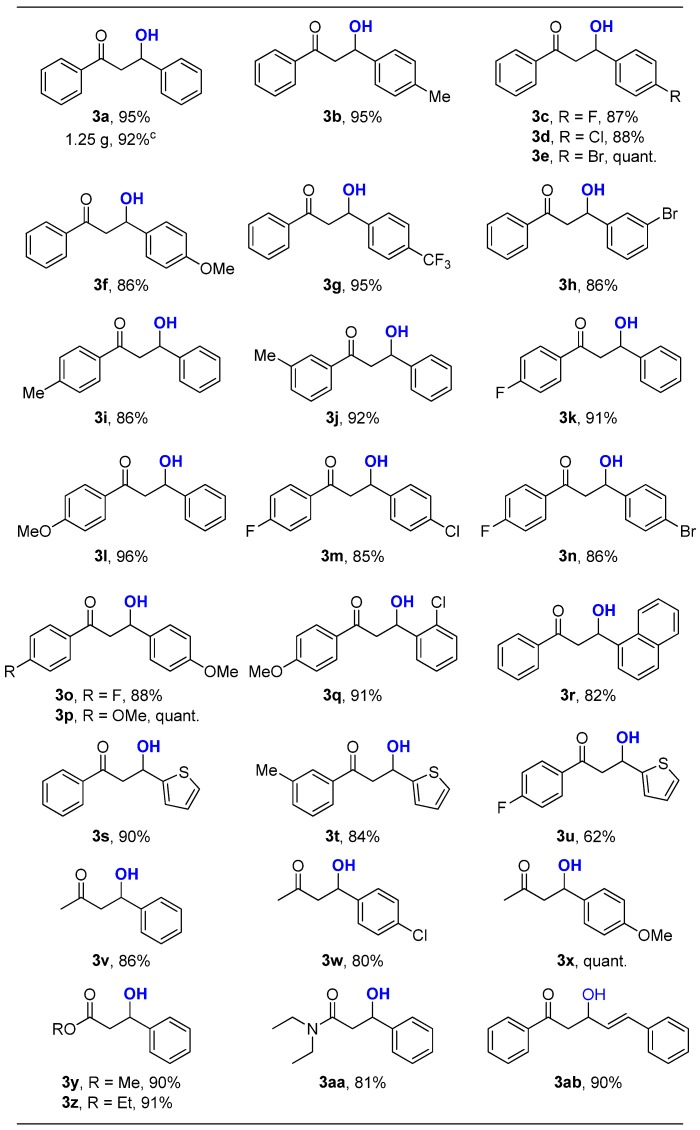
Substrate scope of α,β-unsaturated acceptors ^a,b^. ^a^ Reaction conditions: substrate **2** (0.2 mmol), B_2_(pin)_2_ **1** (1.2 equiv.), CP@Cu NPs (1 mol % Cu loading), acetone (1.6 mL), H_2_O (0.4 mL), room temperature, air, 12 h; ^b^ Isolated yields were listed; ^c^ Gram scale synthesis.

The substrate scope of α,β-unsaturated acceptors was surveyed under the optimized conditions and the results are summarized in Figure 2. Chalcone derivatives with different substituted groups including halides at 4-position all proceeded smoothly to give the desired products in good yields (**3b**–**3g**) (**3b**–**3z**, **3aa** and **3ab** are in Supplementary Materials). Both electro-donating (**3f**) and electron-withdrawing (**3g**) functional groups were tolerated in this borylation process. Comparable results were found using either 2-bromo or 4-bromo substituted chalcone as the substrate (**3h**). Next, 4′-substituted chalcones were explored and good to excellent yields were obtained in all cases (**3i**–**3l**). Besides mono-substituted chalcones, it was found that di-substituted chalcones were also suitable substrates (**3m**–**3q**). Notably, 4,4′-dimethoxy chalcone was converted to the corresponding β-hydroxyl product **3p** quantitatively via its β-boryl intermediate. The reaction can also be carried out when the substrates containing naphthalene or thiophene structures (**3r**–**3u**). Borylation of α,β-unsaturated ketones bearing aliphatic-aromatic motifs resulted in good yields as well as chalcone derivatives (**3v**–**3x**). Ester and amide proved to be difficult substrates which were not disclosed in previous report [41]. Gratifyingly, good conversions of esters and amide were accomplished applying our newly developed method (**3y**, **3z**, and **3aa**). When α,β,γ,δ-dienone was used as substrate, 1,4-addition product was obtained exclusively and 1,6-addition byproduct was not detected. In addition, gram scale synthesis of β-hydroxyl chalcone **3a** (in Supplementary Materials) was realized, which explored the perspective for industrial application of this CP@Cu NPs catalyst. Therefore, it was demonstrated that quite a broad range of α,β-unsaturated acceptors could be borylated effectively, catalyzing by CP@Cu NPs catalyst in aqueous media under mild conditions.

**Figure 3:**
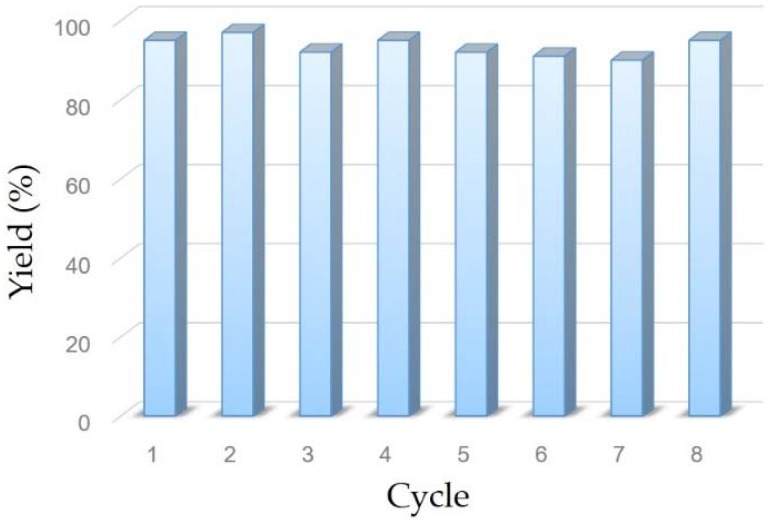
Recycling for the chitosan/poly (vinyl alcohol) composite film supported copper nanoparticles.

In our experiment, the chitosan/poly (vinyl alcohol) composite film with stabilized copper nanoparticles was easily removed from the reaction mixture and recovered by simple filtration. The CP@Cu NPs may perform as a heterogeneous catalyst in the whole reaction system. To evaluate this assumption, the recyclability of the copper nanoparticles was tested in the reaction of chalcone **2a** with bis(pinacolato)diboron **1**. Remarkably, the catalyst kept excellent activity even after being used eight cycles, as shown in Figure 3. In each cycle, fresh substrate and reagent was added to the recovered copper nanoparticles and reacted under standard conditions. Until now, only one example of the recycling of nanoparticle catalyst for similar borylation of chalcone analog has been reported [41]. However, an obvious decrease of reactivity was observed. Then, we quenched the reaction by removing the catalyst when it reached about 60% conversion of starting material. The rest of reaction mixture failed to generate additional product, even with extension of reaction time. In addition, no detectable copper leaching was found in the residue as illustrated by ICP analysis. These results strongly indicated that the CP@Cu NPs was a highly active heterogeneous catalyst for the borylation of α,β-unsaturated acceptors. It displayed several advantages such as easy isolation, operational simplicity and prominent ability of recycle. 

## 4. Conclusions

In conclusion, we have demonstrated copper nanoparticles supported on a chitosan/poly (vinyl alcohol) composite film were effective to catalyze the boron conjugate additions of α,β-unsaturated acceptors in aqueous media at room temperature. Various α,β-unsaturated ketones, esters and amides were successfully applied as the substrates and desired products were all obtained in good to excellent yields. A simple one-pot synthesis of β-hydroxyl ketones was proven by sequential borylation and oxidation processes. In addition, 1,4-addition took place selectively when α,β,γ,δ-dienone was used as a substrate. Remarkably, the copper nanoparticles could be easily recovered and reused eight cycles without any significant decrease of activity. Using chitosan as the support and water as the co-solvent makes our methodology green and sustainable. The advantages of this newly described method include recyclability of catalyst, simple operation, mild conditions and good functional group compatibility. 

## Figures and Tables

**Table 1:**
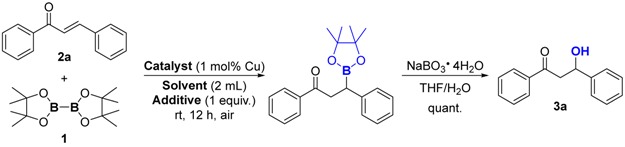
Optimization of the reaction conditions ^a^.

Entry	Catalyst	Solvent	Additive	Yield(%) ^b^
1	CP@Cu NPs	toluene	--	NR ^c^
2	CP@Cu NPs	Et_2_O	--	NR ^c^
3	CP@Cu NPs	THF	--	trace
4	CP@Cu NPs	toluene	MeOH	7
5	CP@Cu NPs	Et_2_O	MeOH	6
6	CP@Cu NPs	THF	MeOH	32
7	CP@Cu NPs	acetone	MeOH	66
8	CP@Cu NPs	MeOH	--	70
9 ^d^	CP@Cu NPs	THF/H_2_O = 2/1	--	75
10 ^d^	CP@Cu NPs	MeOH/H_2_O = 2/1	--	89
11 ^d^	CP@Cu NPs	acetone/H_2_O = 2/1	--	91
12 ^d^	CP@Cu NPs	acetone/H_2_O = 4/1	--	95
13 ^d^	CP@Cu NPs	acetone/H_2_O = 1/4	--	76
14 ^d^	--	acetone/H_2_O = 4/1	--	NR ^c^
15 ^d,e^	CS@Cu	acetone/H_2_O = 4/1	--	35
16 ^d,f^	CS/PEG@Cu NPs	acetone/H_2_O = 4/1	--	75
15 ^d,g^	CP@Cu NPs	acetone/H_2_O = 4/1	--	89
16 ^c,h^	CP@Cu NPs	acetone/H_2_O = 4/1	--	94

^a^ Reaction conditions: substrate **2** (0.2 mmol), B_2_(pin)_2_ **1** (1.2 equiv), catalyst (1 mol % Cu loading), Solvent (2 mL), room temperature, air, 12h; ^b^ Isolated yield of product; ^c^ NR = no reaction; ^d^ Ratio of volume to volume; ^e^ Chitosan supported Cu was used; ^f^ Chitosan/poly (ethylene glycol) composite film supported Cu nanoparticles were used; ^g^ 0.5 mol % Cu loading was used; ^h^ Performed under Ar atmosphere.

## Supplementary Materials

The following are available online at http://www.mdpi.com/2079-4991/8/5/326/s1, characterization data, and spectra for the ^1^H and ^13^C NMR of compounds **3a**–**3****z**, **3aa** and **3ab**; SEM image and IR spectra of the prepared CP@Cu NPs.
