# Supplementary material for: Borylation of α,β-Unsaturated Acceptors by Chitosan Composite Film Supported Copper Nanoparticles
Source: Nanomaterials (Basel). 2018 May 14;8(5):326. doi: 10.3390/nano8050326 (PMC5977340; doi:10.3390/nano8050326)
Supplement: Supplementary file 1 [file nanomaterials-08-00326-s001.pdf]

Borylation of α,β-Unsaturated Acceptors by Chitosan Composite Film Supported Copper Nanoparticles

Wu Wen,[1] Biao Han,[1] Feng Yan,[1]Liang Ding,[2,*], Bojie Li,[1]
Liansheng Wang,[1,*]and Lei Zhu,[1,*]

1 School of Chemistry and Materials Science, Hubei Engineering University, Hubei Collaborative Innovation Center of Conversion and Utilization for Biomass Resources, Xiaogan 432000, China

2 Department of Polymer and Composite Material, School of Materials Engineering, Yancheng Institute of Technology, Yancheng 224051, China

3 School of Materials Science and Engineering, Hubei University, Wuhan 430062, China

Supporting Information

Table of Contents

1. Characterization Data for β-Hydroxyl Products S2-S11
2. ^{1}H NMR and ^{13}C NMR Spectra S13-S40
3. IR Spectra of CP@Cu Nanoparticles S41
4. SEM Image of CP@Cu Nanoparticles S41

1. Characterization Data for *β*-Hydroxyl Products

All of adducts are literature-known; obtained characterization data for these compounds is in full agreement with reported data.

3-Hydroxy-1,3-diphenylpropan-1-one（**3a**）

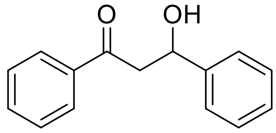

Colorless oil

^{1}H NMR (400 MHz); δ = 7.97 (d, *J* = 7.1 Hz, 2H), 7.61-7.57 (m, 1H), 7.49-7.44 (m, 4H), 7.40-7.37 (m, 2H), 7.33-7.29 (m, 1H), 5.37 (t, *J* = 6.1 Hz, 1H), 3.39 (d, *J* = 6.1 Hz, 2H).

^{13}C NMR (100 MHz); δ = 200.0, 142.9, 136.5, 133.6, 128.6, 128.5, 128.1, 127.6, 125.7, 69.9, 47.3.

MS (ESI): *m/z* 227 $[M+H]^+$.

3-Hydroxy-1-phenyl-3-(*p*-tolyl)propan-1-one (**3b**)

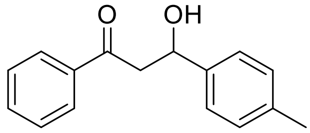

White solid

^{1}H NMR (400 MHz); δ = 7.97 (d, *J* = 7.0 Hz, 2H), 7.61 (m, *J* = 7.4 Hz, 1H), 7.49-7.45 (m, 2H), 7.35 (d, *J* = 8.0 Hz, 2H), 7.21 (d, *J* = 7.8 Hz, 2H), 5.34-5.30 (m, 1H), 3.64 (d, *J* = 2.3 Hz, 1H), 3.38-3.35(m, 2H), 2.37 (s, 3H).

^{13}C NMR (100 MHz); δ = 200.1, 139.9, 137.2, 136.5, 133.5, 129.1, 128.6, 128.1, 125.6, 69.8, 47.3, 21.1.

MS (ESI): *m/z* 241 $[M+H]^+$.

3-(4-Fluorophenyl)-3-hydroxy-1-phenylpropan-1-one (**3c**)

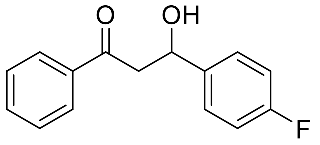

White solid

^{1}H NMR (400 MHz); δ = 7.96 (d, *J* = 7.0 Hz, 2H), 7.62-7.58 (m, 2H), 7.49-7.40 (m, 4H), 7.06 (t, *J* = 8.7 Hz, 2H), 5.34-5.32 (m, 1H), 3.36-3.34 (m, 2H).

^{13}C NMR (100 MHz); δ = 200.0, 163.4, 160.9, 138.69, 138.66, 136.4, 133.7, 128.7, 128.1, 127.44, 127.36, 115.4, 115.2, 69.4, 47.3.

MS (ESI): *m*/*z* 245 $[M+H]^+$.

3-(4-Chlorophenyl)-3-hydroxy-1-phenylpropan-1-one (**3d**)

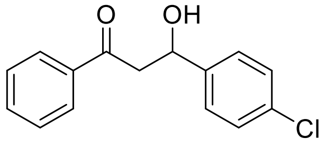

White solid

^{1}H NMR (400 MHz); δ = 7.96 (d, *J* = 7.0 Hz, 2H), 7.62 (t, *J* = 7.4 Hz, 1H), 7.50 (t, *J* = 7.9 Hz, 2H), 7.39-7.34 (m, 4H), 5.34 (m, 1H), 3.35-3.33 (m, 2H).

^{13}C NMR (100 MHz); δ = 199.8, 141.4, 136.3, 133.7, 133.2, 128.64, 128.56, 128.1, 127.1, 69.3, 47.2.

MS (ESI): *m*/*z* 262 $[M+H]^+$.

3-(4-Bromophenyl)-3-hydroxy-1-phenylpropan-1-one (**3e**)

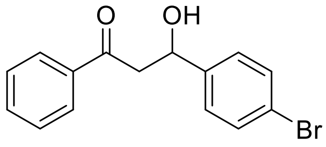

White solid

^{1}H NMR (400 MHz); δ = 7.95 (d, *J* = 7.0 Hz, 2H), 7.62 (t, *J* = 7.4 Hz, 1H), 7.51-7.45 (m, 4H), 7.33 (d, *J* = 8.4 Hz, 2H), 5.32 (dd, *J* = 7.4, 4.8 Hz, 1H), 3.68 (br, 1H), 3.38-3.28 (m, 2H).

^{13}C NMR (100 MHz); δ = 199.9, 141.9, 136.3, 133.8, 131.6, 128.7, 128.1, 127.5, 121.4, 69.4, 47.2.

MS (ESI): *m*/*z* 306 [M+H] $^{+}$.

3-Hydroxy-3-(4-methoxyphenyl)-1-phenylpropan-1-one (**3f**)

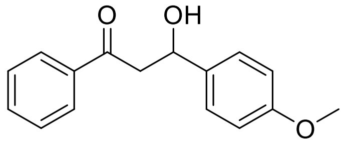

White solid

^{1}H NMR (400 MHz); δ = 7.97 (d, *J* = 7.0 Hz, 2H), 7.61 (t, *J* = 7.4 Hz, 1H), 7.49-7.45 (m, 2H), 7.38 (t, *J* = 8.5 Hz, 2H), 6.93-6.91 (m, 2H), 5.32 (dd, *J* = 7.1, 5.0 Hz, 1H), 3.82 (s, 3H), 3.38-3.33 (m, 2H).

^{13}C NMR (100 MHz); δ = 200.2, 159.0, 136.5, 135.1, 133.6, 128.6, 128.1, 127.0, 113.9, 69.6, 55.2, 47.3.

MS (ESI): *m*/*z* 257 [M+H] $^{+}$.

3-Hydroxy-1-phenyl-3-(4-(trifluoromethyl)phenyl)propan-1-one (**3g**)

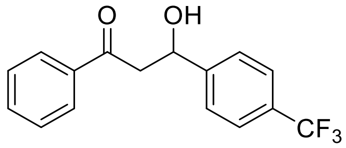

White solid

^{1}H NMR (400 MHz); δ = 7.96 (d, *J* = 7.4 Hz, 2H), 7.65-7.57 (m, 5H), 7.50 (t, *J* = 7.7 Hz, 2H), 5.43 (dd, *J* = 8.6, 3.4 Hz, 1H), 3.38-3.35 (m, 2H).

^{13}C NMR (100 MHz); δ = 199.8, 146.9, 136.3, 133.9, 130.0, 129.6, 128.8, 128.1, 126.0, 125.499, 125.495, 125.46, 125.42, 122.7, 68.4, 47.2.

MS (ESI): *m*/*z* 295 [M+H] $^{+}$.

3-(3-Bromophenyl)-3-hydroxy-1-phenylpropan-1-one (**3h**)

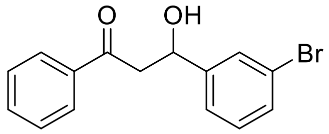

White solid

^{1}H NMR (400 MHz); δ = 7.95 (d, *J* = 7.5 Hz, 2H), 7.61-7.57 (m, 2H), 7.48-7.21 (m, 5H), 5.37 (m, 1H), 3.82 (br, 1H), 3.34-3.32 (m, 2H).

^{13}C NMR (100 MHz); δ = 199.7, 145.2, 136.2, 133.7, 130.6, 130.0, 128.8, 128.6, 128.1, 124.3, 122.6, 69.1, 47.1.

MS (ESI): *m*/*z* 306 $[M+H]^{+}$.

3-Hydroxy-3-phenyl-1-(*p*-tolyl)propan-1-one (**3i**)

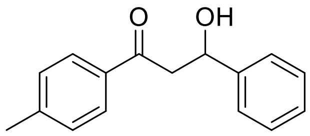

White solid

^{1}H NMR (400 MHz); δ = 7.84 (d, *J* = 8.3 Hz, 2H), 7.43 (d, *J* = 6.8 Hz, 2H), 7.37-7.34 (m, 2H), 7.30-7.23 (m, 3H), 5.33-5.29 (m, 1H), 3.76 (d, *J* = 2.9 Hz, 1H), 3.33 (d, *J* = 6.0 Hz, 2H), 2.39 (s, 3H).

^{13}C NMR (100 MHz); δ = 199.8, 144.5, 142.9, 134.0, 129.3, 128.4, 128.2, 127.5, 125.7, 70.0, 47.1, 21.6.

MS (ESI): *m*/*z* 241 $[M+H]^{+}$.

3-Hydroxy-3-phenyl-1-(*m*-tolyl)propan-1-one (**3j**)

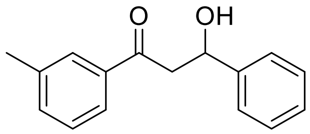

White solid

^{1}H NMR (400 MHz); δ = 7.76 (d, *J*= 8.3 Hz, 2H), 7.46-7.29 (m, 7H), 5.36-5.33 (m, 1H), 3.37 (br, 2H), 2.40 (s, 3H).

^{13}C NMR (100 MHz); δ = 200.3, 142.9, 138.4, 136.5, 134.2, 128.6, 128.5, 128.4, 127.5, 125.7, 125.3, 69.9, 47.4, 21.2.

MS (ESI): *m*/*z* 241 $[M+H]^{+}$.

1-(4-Fluorophenyl)-3-hydroxy-3-phenylpropan-1-one (**3k**)

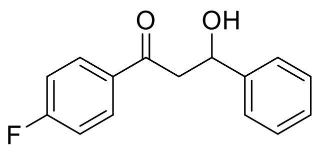

White solid

^{1}H NMR (400 MHz); δ = 7.99-7.96 (m, 2H), 7.44-7.42 (m, 2H), 7.40 (t, *J* = 7.6 Hz, 2H), 7.32 (t, *J* = 7.2 Hz, 1H), 7.15-7.10 (m, 2H), 5.35-5.32 (m, 1H), 3.63 (d, *J* = 3.0 Hz, 1H), 3.40-3.27 (m, 2H).

^{13}C NMR (100 MHz); δ = 198.3, 167.2, 164.7, 142.8, 133.0, 132.9, 130.8, 130.7, 128.5, 127.7, 125.7, 115.9, 115.6, 69.9, 47.3.

MS (ESI): *m*/*z* 245 $[M+H]^+$.

3-Hydroxy-1-(4-methoxyphenyl)-3-phenylpropan-1-one (**3l**)

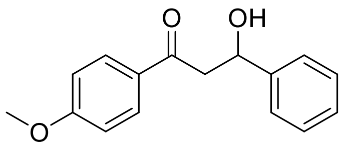

White solid

^{1}H NMR (400 MHz); δ = 7.95 -7.93 (m, 2H), 7.45-7.26 (m, 5H), 6.94 (d, *J* = 9.0 Hz, 2H), 5.34 (dd, *J*= 8.4, 3.7 Hz, 1H), 3.87 (s, 3H), 3.33-3.30 (m, 2H).

^{13}C NMR (100 MHz); δ = 198.7, 163.8, 143.0, 130.4, 129.5, 128.4, 127.5, 125.7, 113.7, 70.0, 55.4, 46.8.

MS (ESI): *m*/*z* 257 $[M+H]^+$.

3-(4-Chlorophenyl)-1-(4-fluorophenyl)-3-hydroxypropan-1-one (**3m**)

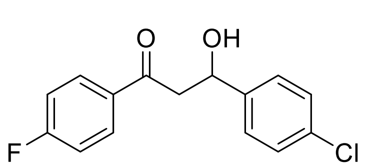

White solid

^{1}H NMR (400 MHz); δ = 7.99 (dd, *J* = 8.9, 5.3 Hz, 2H), 7.38 (dd, *J* = 12.2, 8.7 Hz, 4H), 7.14 (t, *J* = 8.6 Hz, 2H), 5.33-5.30 (m, 1H), 3.31-3.30 (m, 2H).

^{13}C NMR (100 MHz); δ = 198.2, 167.4, 164.8, 141.3, 133.4, 132.9, 132.8, 130.9, 130.8, 128.7, 127.1, 116.0, 115.8, 69.3, 47.2.

MS (ESI): *m/z* 280 $[M+H]^+$.

3-(4-Bromophenyl)-1-(4-fluorophenyl)-3-hydroxypropan-1-one (**3n**)

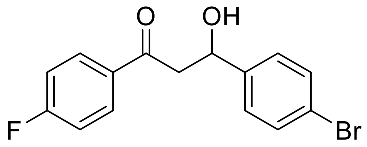

White solid

^{1}H NMR (400 MHz); δ = 7.99 (dd, *J* = 8.9 Hz, 5.4 Hz, 2H), 7.51 (d, *J* = 8.4 Hz, 1H), 7.32 (d, *J* = 8.4 Hz, 2H), 7.14 (t, *J* = 8.6 Hz, 2H), 5.31-5.28 (m, 1H), 3.59 (br, 1H), 3.30 (d, *J* = 5.9 Hz, 2H).

^{13}C NMR (100 MHz); δ = 198.2, 167.4, 164.8, 141.8, 132.83, 132.81, 131.7, 130.9, 130.8, 127.4, 121.5, 116.0, 115.8, 69.4, 47.1.

MS (ESI): *m/z* 324 $[M+H]^+$.

1-(4-Fluorophenyl)-3-hydroxy-3-(4-methoxyphenyl)propan-1-one (**3o**)

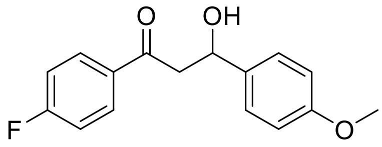

White solid

^{1}H NMR (400 MHz); δ = 8.00-7.97 (m, 2H), 7.37 (d, *J* = 8.6 Hz, 2H), 7.16 (t, *J* = 8.6 Hz, 2H), 6.92 (d, *J* = 8.7 Hz, 2H), 5.31-5.27 (m, 1H), 3.81 (s, 3H), 3.45 (br, 1H), 3.35-3.31 (m, 2H).

^{13}C NMR (100 MHz); δ = 198.4, 167.2, 164.6, 159.0, 135.0, 133.0, 132.98, 130.8, 130.7, 126.9, 115.8, 115.6, 113.8, 69.6, 55.2, 47.2.

MS (ESI): *m/z* 275 $[M+H]^+$.

3-Hydroxy-1,3-bis(4-methoxyphenyl)propan-1-one (**3p**)

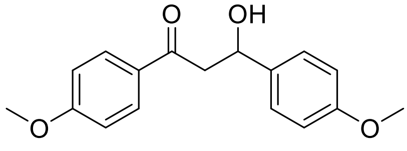

White solid

^{1}H NMR (400 MHz); δ = 7.95 (d, *J* = 8.9 Hz, 2H), 7.37 (d, *J* = 8.6 Hz, 2H), 6.94 (t, *J* = 8.3 Hz, 4H), 5.29-5.26 (m, 1H), 3.87 (s, 3H), 3.81 (s, 3H), 3.31-3.29 (m, 2H).

^{13}C NMR (100 MHz); δ = 198.8, 163.9, 159.0, 135.2, 130.5, 129.6, 127.0, 113.9, 113.8, 69.8, 55.5, 55.3, 46.8.

MS (ESI): *m*/*z* 287 [M+H]$^{+}$.

3-(2-Chlorophenyl)-3-hydroxy-1-(4-methoxyphenyl)propan-1-one (**3q**)

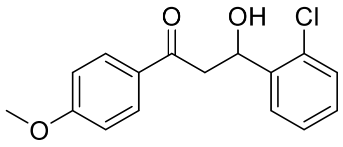

White solid

^{1}H NMR (400 MHz); δ = 7.93 (d, *J* = 9.0 Hz, 2H), 7.71.-7.68 (dd, *J* = 7.5, 1.6 Hz, 1H), 7.33-7.29 (m, 2H), 7.24-7.18 (m, 1H), 6.93 (d, *J* = 9.0 Hz, 2H), 5.66-5.62 (m, 1H), 4.06 (br, 1H), 3.84 (s, 3H), 3.52-3.47 (dd, *J* = 17.5, 2.2 Hz, 1H), 3.08-3.02 (dd, *J* = 17.5, 9.6 Hz, 1H).

^{13}C NMR (100 MHz); δ=198.8, 163.9, 140.4, 131.1, 130.5, 129.4, 129.2, 128.4, 127.23, 127.18, 113.8, 66.9, 55.5, 44.8.

MS (ESI): *m*/*z* 292 [M+H]$^{+}$.

3-Hydroxy-3-(naphthalen-1-yl)-1-phenylpropan-1-one (**3r**)

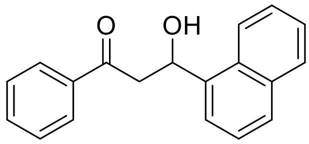

White solid

^{1}H NMR (400 MHz); δ = 8.07-8.05 (m, 1H), 8.00-7.90 (m, 3H), 7.83 (d, *J* = 7.6 Hz, 2H), 7.60-7.43 (m, 6H), 6.19-6.15 (m, 1H), 3.86 (br, 1H), 3.58-3.45 (m, 2H).

^{13}C NMR (100 MHz); δ = 200.2, 138.4, 136.4, 133.7, 133.6, 129.8, 129.0, 128.6, 128.1, 128.0, 126.1, 125.6, 125.5, 123.1, 122.7, 66.7, 46.7.

MS (ESI): *m*/*z* 277 [M+H]$^{+}$.

3-Hydroxy-1-phenyl-3-(thiophen-2-yl)propan-1-one (**3s**)

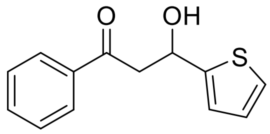

White solid

^{1}H NMR (400 MHz); δ = 7.99-7.97 (m, 2H), 7.61-7.50 (m, 1H), 7.50-7.46 (m, 2H), 7.29-7.26 (m, 1H), 7.04-6.98 (m, 2H), 5.62 (m, 1H), 3.79 (s, 1H), 3.53-3.50 (m, 2H).

^{13}C NMR (100 MHz); δ = 199.6, 146.6, 136.4, 133.8, 128.7, 128.1, 126.7, 124.7, 123.5, 66.4, 47.1.

MS (ESI): *m*/*z* 233 [M+H]$^{+}$.

3-Hydroxy-3-(thiophen-2-yl)-1-(*m*-tolyl)propan-1-one (**3t**)

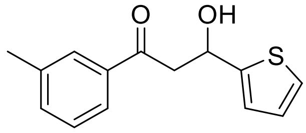

White solid

^{1}H NMR (400 MHz); δ = 7.78 (d, *J* = 7.9 Hz, 2H), 7.42-7.35 (m, 2H), 7.28 (dd, *J* = 5.0, 1.2 Hz, 2H), 7.05 (d, *J* = 3.5 Hz, 1H), 7.00 (dd, *J* = 5.0, 3.5 Hz , 1H), 5.60 (t, *J* = 5.8 Hz 1H), 3.50 (d, *J* = 5.9 Hz, 2H), 2.42 (s, 3H).

^{13}C NMR (100 MHz); δ = 199.9, 146.7, 138.6, 136.4, 134.5, 128.63, 128.59, 126.7, 125.4, 124.7, 123.5, 66.5, 47.2, 21.3.

MS (ESI): *m*/*z* 247 [M+H]$^{+}$.

1-(4-Fluorophenyl)-3-hydroxy-3-(thiophen-2-yl)propan-1-one (**3u**)

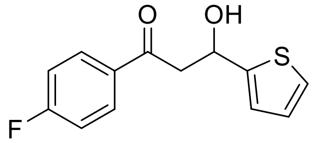

White solid

^{1}H NMR (400 MHz); δ = 8.01-7.98 (dd, *J* = 7.6, 5.4 Hz, 2H), 7.28-7.26 (m, 1H), 7.17-7.13 (m, 2H), 7.03-6.97 (m, 2H), 5.61-5.58 (m, 1H), 3.70 (br, 1H), 3.54-3.41 (m, 2H).

^{13}C NMR (100 MHz); δ = 197.9, 167.4, 164.8, 146.5, 132.9, 132.8, 130.9, 130.8, 126.7, 124.7, 123.6, 116.0, 115.8, 66.4, 47.1.

MS (ESI): *m/z* 251 $[M+H]^+$.

4-Hydroxy-4-phenylbutan-2-one (**3v**)

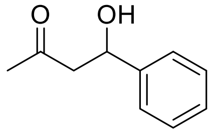

Colorless oil

^{1}H NMR (400 MHz); δ = 7.37 (d, *J* = 4.4 Hz, 4H), 7.31-7.28 (m, 1H), 5.18-5.14 (m, 1H), 3.41 (br, 1H), 2.93-2.78 (m, 2H), 2.20 (s, 3H).

^{13}C NMR (100 MHz); δ = 209.1, 142.6, 128.5, 127.7, 125.6, 69.8, 51.9, 30.8.

MS (ESI): *m/z* 165 $[M+H]^+$.

4-(4-Clorophenyl)-4-hydroxybutan-2-one (**3w**)

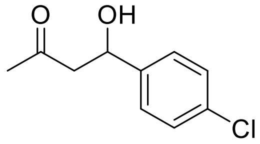

White solid

^{1}H NMR (400 MHz); δ = 7.38-7.32 (m, 4H), 5.19-5.16 (m, 1H), 3.2 (br, 1H), 2.89-2.85 (m, 2H), 2.25 (s, 3H).

^{13}C NMR (100 MHz); δ = 209.0, 141.2, 133.2, 128.6, 127.0, 69.1, 51.7, 30.7.

MS (ESI): *m/z* 200 $[M+H]^+$.

4-Hydroxy-4-(4-methoxyphenyl)butan-2-one (**3x**)

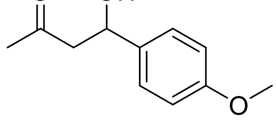

White solid

^{1}H NMR (400 MHz); δ = 7.29-7.26 (m, 2H), 6.89 (d, *J* = 8.7 Hz, 2H), 5.12-5.08 (m, 1H), 3.80 (s, 3H), 3.23 (br, 1H), 2.89-2.76 (m, 2H), 2.19 (s, 3H).

^{13}C NMR (100 MHz); δ = 209.2, 159.1, 134.8, 126.9, 113.9, 69.5, 55.3, 51.9, 30.8.

MS (ESI): *m*/*z* 195 $[M+H]^+$.

Methyl-3-hydroxy-3-phenylpropanoate (**3y**)

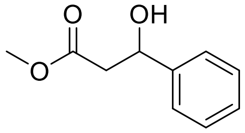

Colorless oil

^1H NMR (400 MHz); δ = 7.39-7.29 (m, 5H), 5.16 (dd, *J* = 8.9, 3.3 Hz, 1H), 3.73 (s, 3H), 2.81-2.89 (m, 2H).

^{13}C NMR (100 MHz); δ = 172.8, 142.4, 128.6, 127.6, 125.6, 70.3, 51.9, 43.1.

MS (ESI): *m*/*z* 181 $[M+H]^+$.

Ethyl-3-hydroxy-3-phenylpropanoate (**3z**)

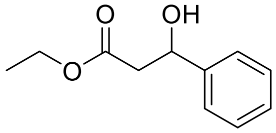

Colorless oil

^1H NMR (400 MHz); δ = 7.39-7.29 (m, 5H), 5.16 (m, 1H), 4.22 (q, *J*= 7.2 Hz, 2H), 2.80-2.68 (m, 2H), 1.29 (t, *J* = 7.0 Hz, 3H).

^{13}C NMR (100 MHz); δ = 172.5, 142.4, 128.5, 127.8, 125.6, 70.3, 60.9, 43.3, 14.1.

MS (ESI): *m*/*z* 195 $[M+H]^+$.

N,*N*-diethyl-3-hydroxy-3-phenylpropanamide (**3aa**)

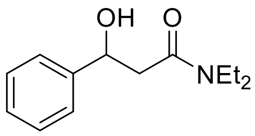

A little bit yellow oil

^1H NMR (400 MHz); δ = 7.41-7.26 (m, 5H), 5.16-5.13 (m, 1H), 3.44-3.34 (m, 2H), 3.26-3.20 (m, 2H), 2.71-2.57 (m, 2H), 1.16-1.10 (m, 6H)

^{13}C NMR (100 MHz); δ = 171.5, 143.1, 128.4, 127.5, 125.7, 70.6, 41.9, 41.5, 40.2, 14.1, 13.0

MS (ESI): *m*/*z* 222 [M+H] $^+$.

(*E*)-3-Hydroxy-1,5-diphenylpent-4-en-1-one (**3ab**)

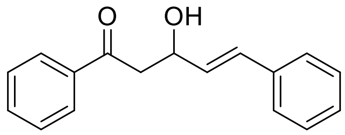

White solid

^{1}H NMR (400 MHz); δ = 7.90 (d, *J* = 7.2 Hz, 2H), 7.54 (t, *J* = 7.3 Hz, 1H), 7.42 (t, *J* = 7.6 Hz, 2H), 7.33 (d, *J* = 7.0 Hz, 2H), 7.26-7.22 (m, 2H), 7.18-7.15 (m, 1H), 6.65 (d, *J* = 15.8 Hz, 1H), 6.27 (dd, *J* = 15.9 Hz, 6.0 Hz, 1H), 4.89-4.86 (m, 1H), 3.38 (d, *J* = 3.7 Hz, 1H), 3.22-3.19 (m, 2H).

^{13}C NMR (100 MHz); δ = 200.0, 136.5, 133.6, 130.4, 130.2, 128.7, 128.5, 128.1, 127.7, 126.5, 68.6, 45.2.

MS (ESI): *m*/*z* 253 [M+H] $^+$.

2. ^{1}H and ^{13}C NMR spectra

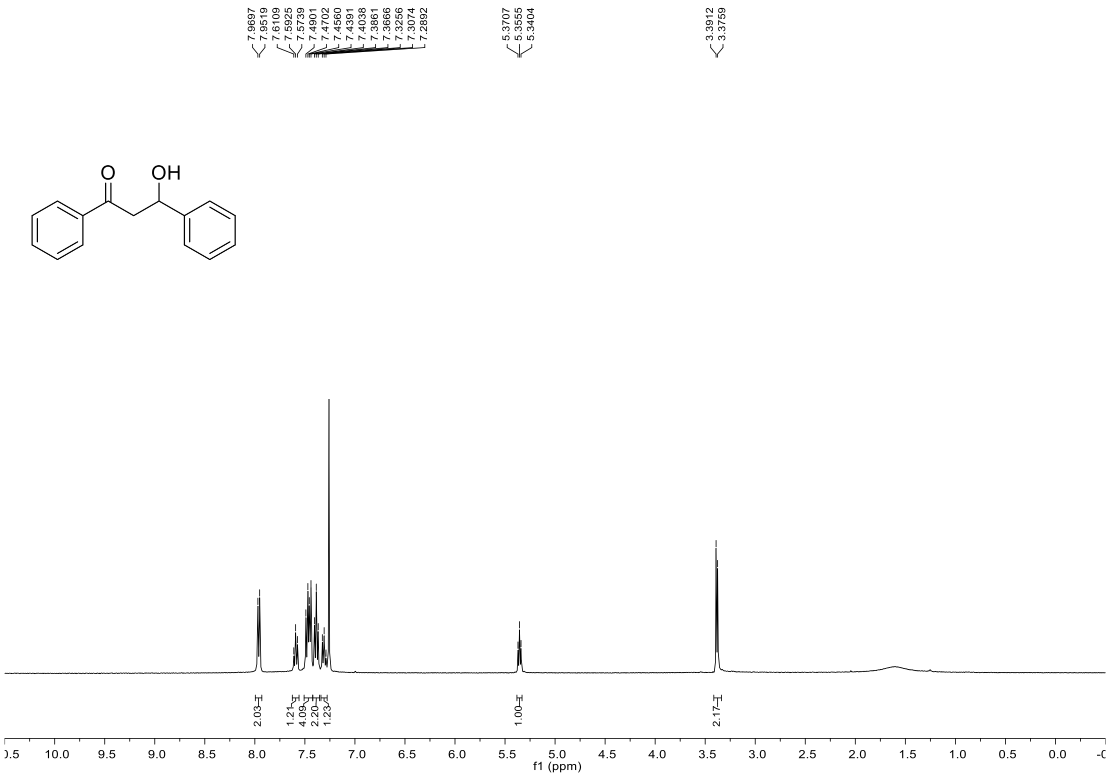

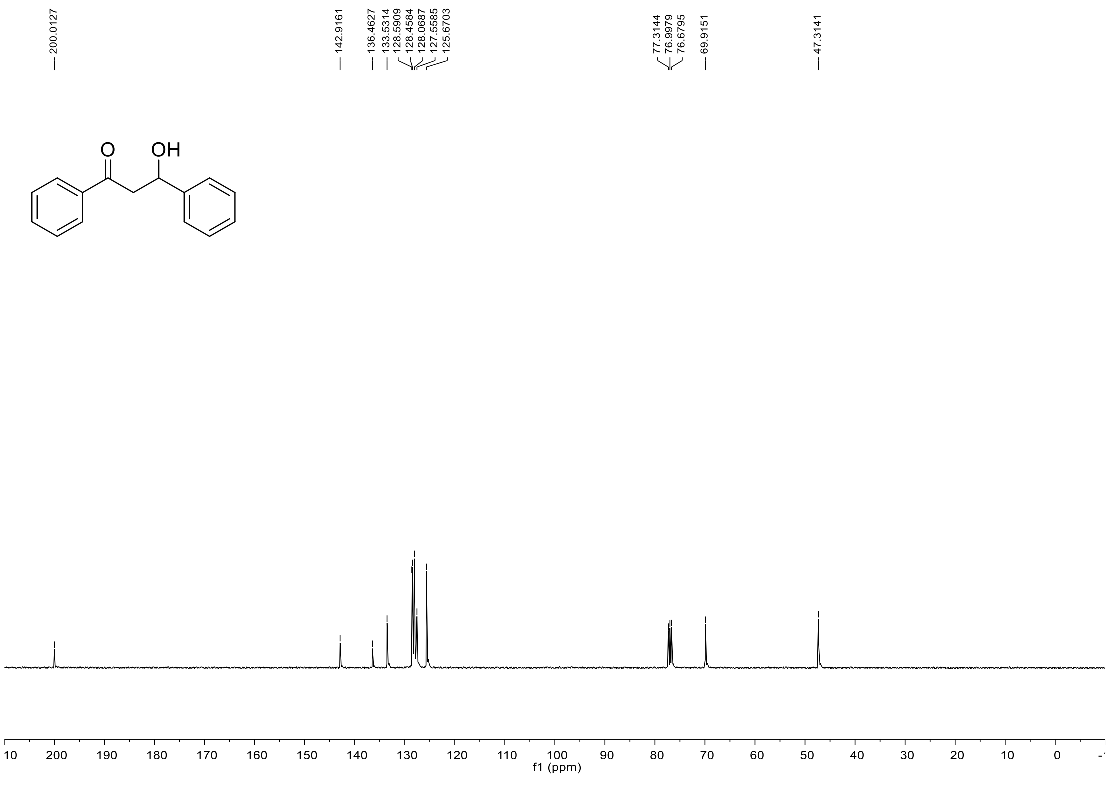

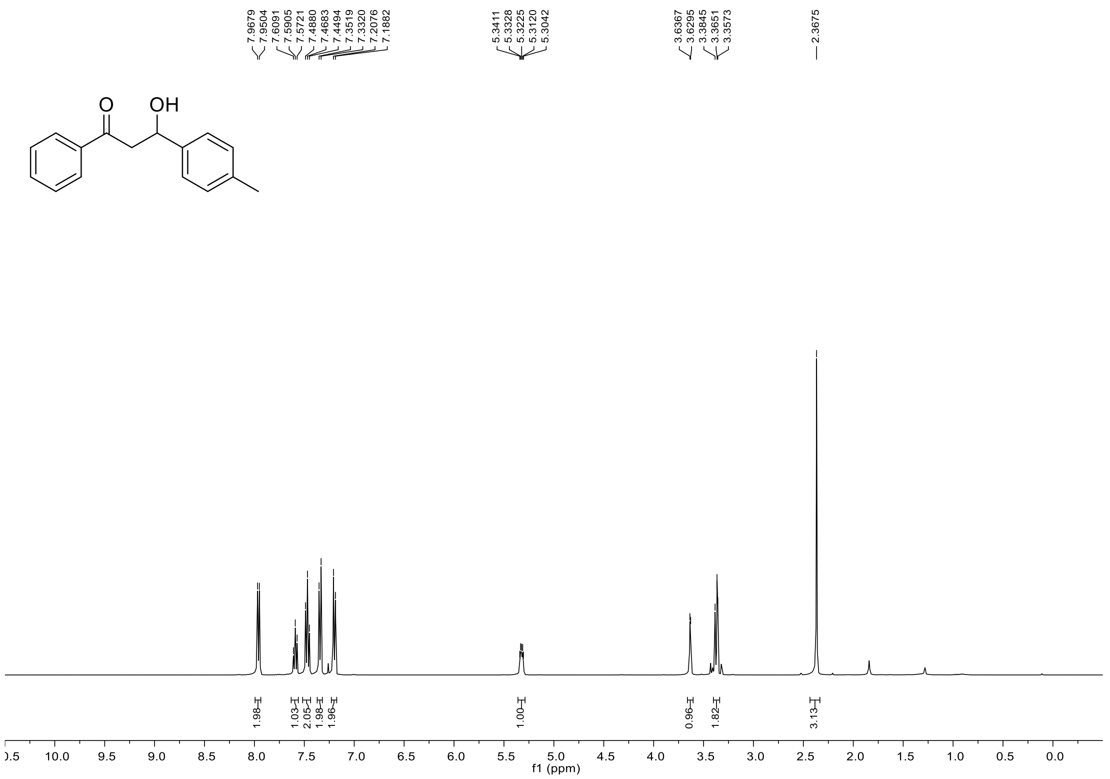
7.9679
7.9504
7.6091
7.5905
7.5721
7.4880
7.4683
7.4494
7.3519
7.3320
7.2076
7.1882
5.3411
5.3328
5.3225
5.3120
5.3042
3.6367
3.6295
3.3845
3.3651
3.3573
2.3675
O
OH
1.98
1.03
2.05
1.98
1.96
1.00
0.96
1.82
3.13
f1 (ppm)

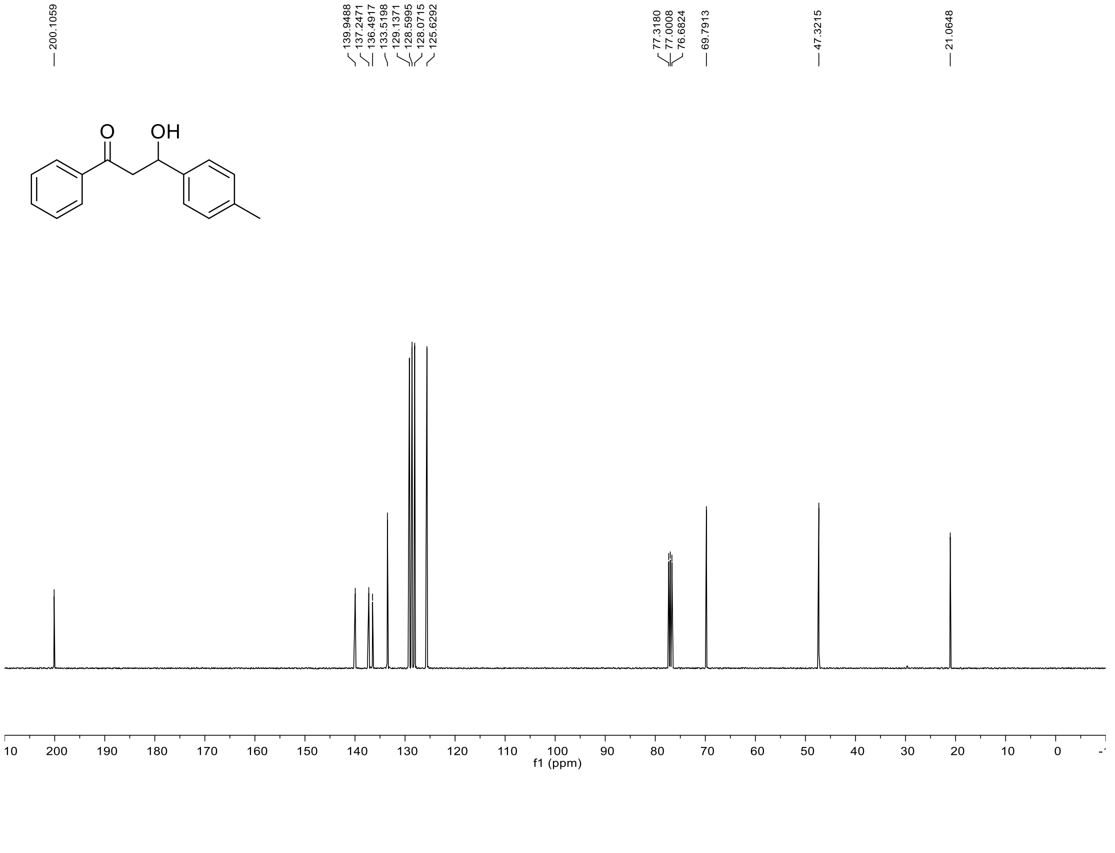
200.1059
139.9488
137.2471
136.4917
133.5198
129.1371
128.5995
128.0715
125.6292
77.3180
77.0008
76.6824
69.7913
47.3215
21.0648
O
OH
f1 (ppm)

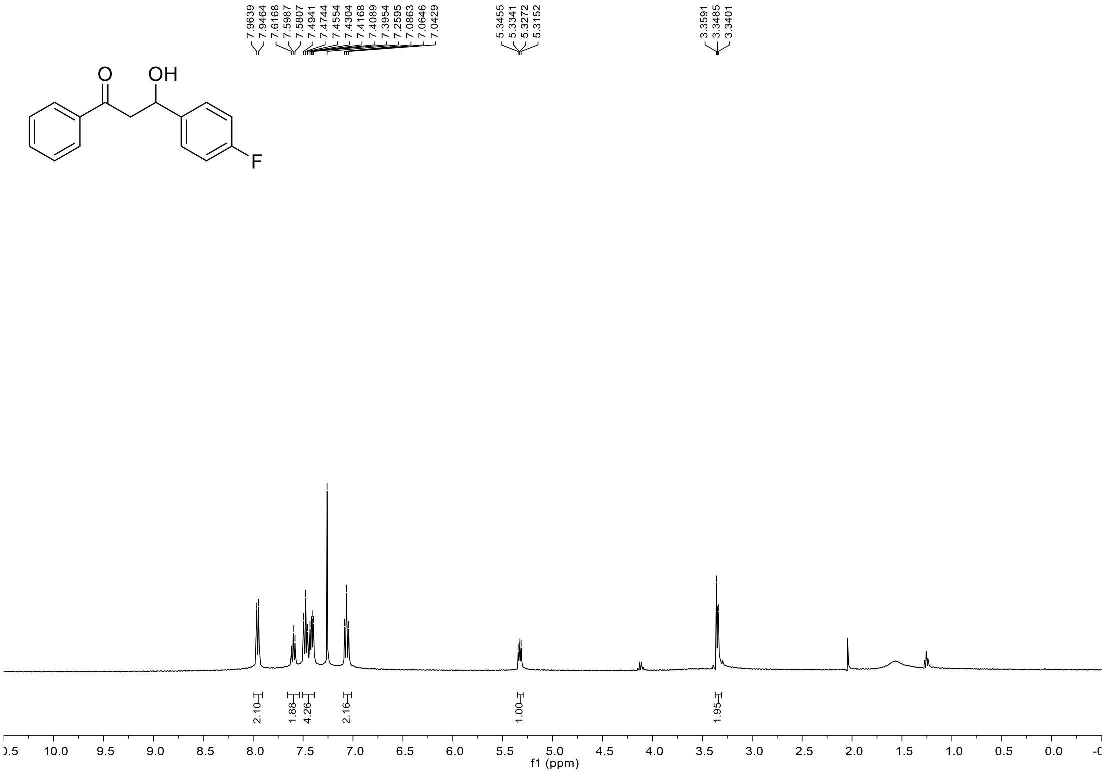
7.9639
7.9464
7.6168
7.5987
7.5807
7.4941
7.4744
7.4554
7.4304
7.4168
7.4089
7.3954
7.2595
7.0863
7.0646
7.0429
5.3455
5.3341
5.3272
5.3152
3.3591
3.3485
3.3401
O
OH
F
2.10
1.88
4.26
2.16
1.00
1.95
f1 (ppm)

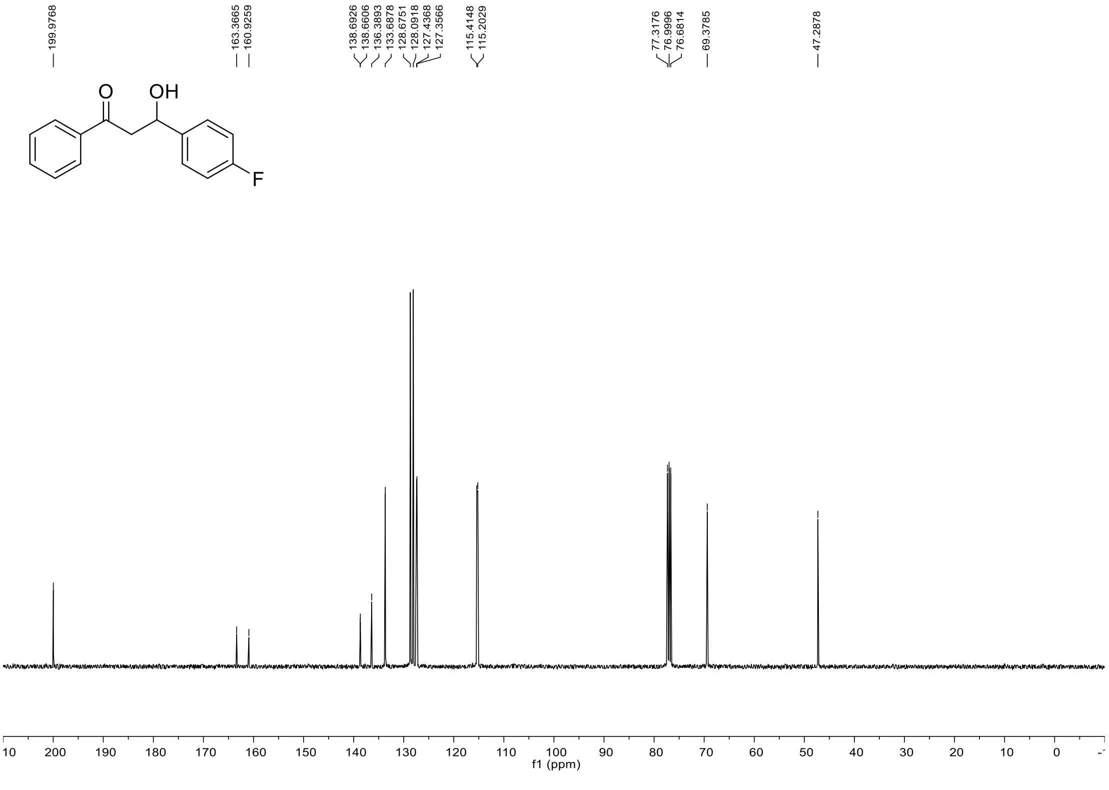
199.9768
163.3665
160.9259
138.6926
138.6606
136.3893
133.6878
128.6751
128.0918
127.4368
127.3566
115.4148
115.2029
77.3176
76.9996
76.6814
69.3785
47.2878
O
OH
F
f1 (ppm)

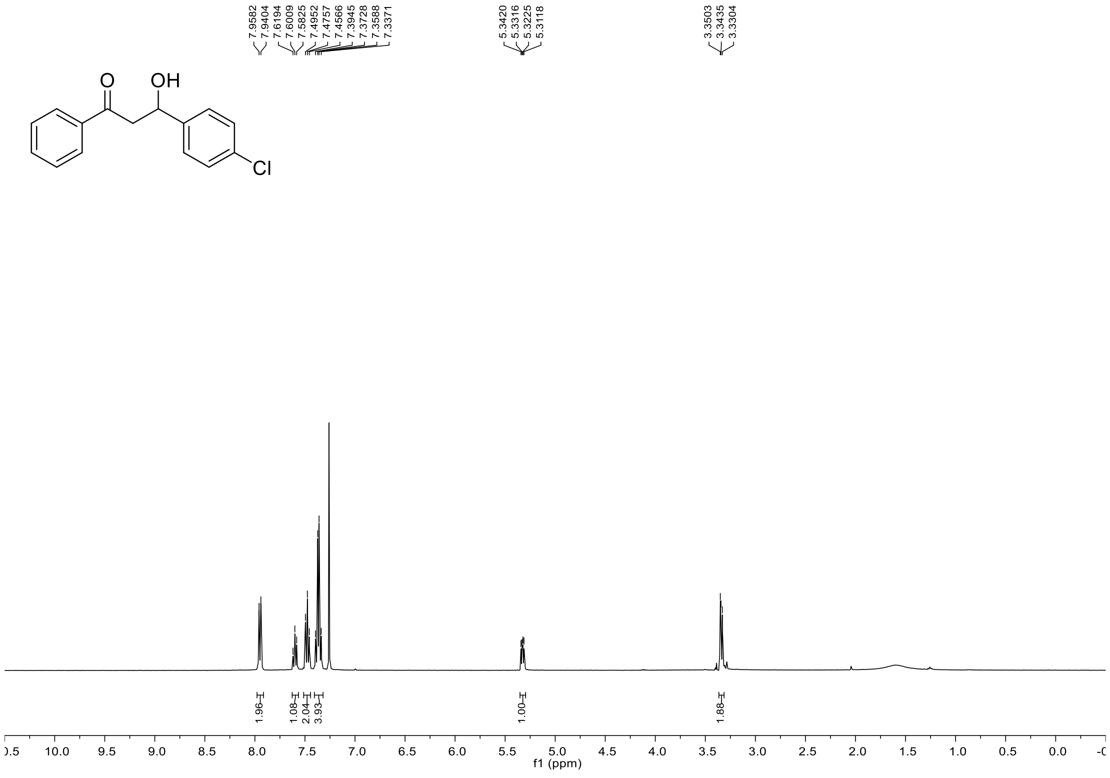
7.9582
7.9404
7.6194
7.6009
7.5825
7.4952
7.4757
7.4566
7.3945
7.3728
7.3588
7.3371
5.3420
5.3316
5.3225
5.3118
3.3503
3.3435
3.3304
O
OH
Cl
1.96
1.08
2.04
3.93
1.00
1.88
f1 (ppm)

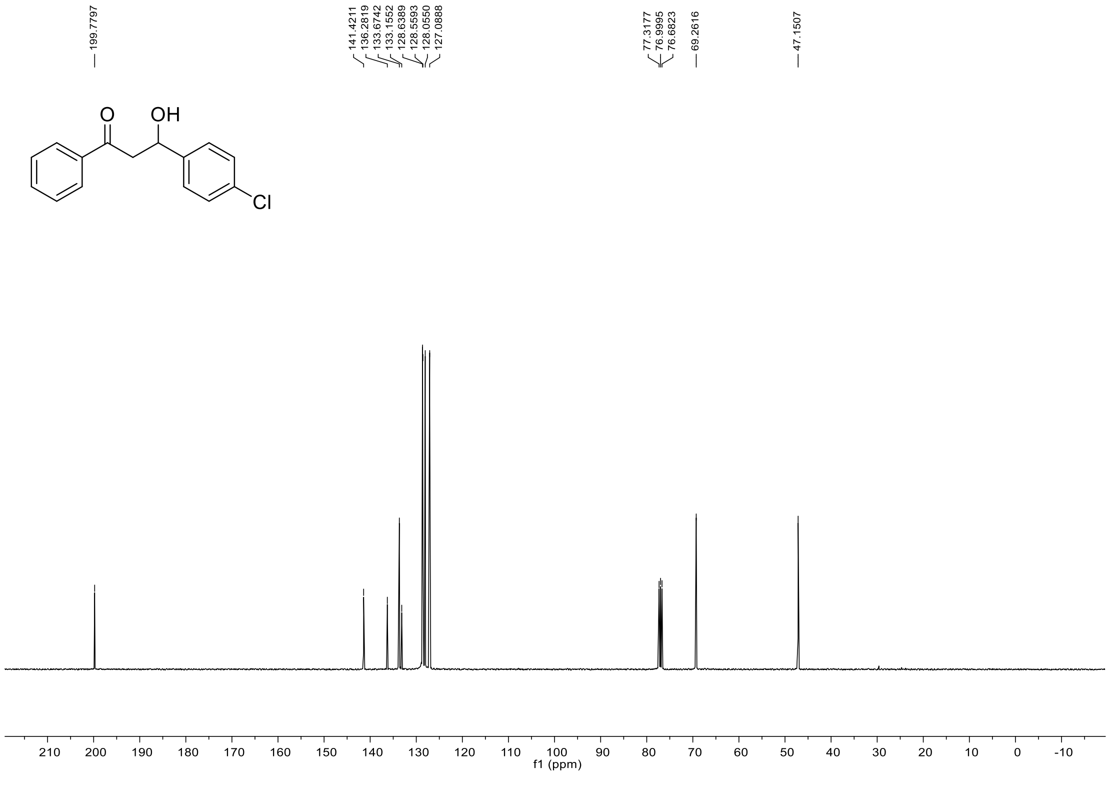
199.7797
141.4211
136.2819
133.6742
133.1552
128.6389
128.5593
128.0550
127.0888
77.3177
76.9995
76.6823
69.2616
47.1507
O
OH
Cl
f1 (ppm)

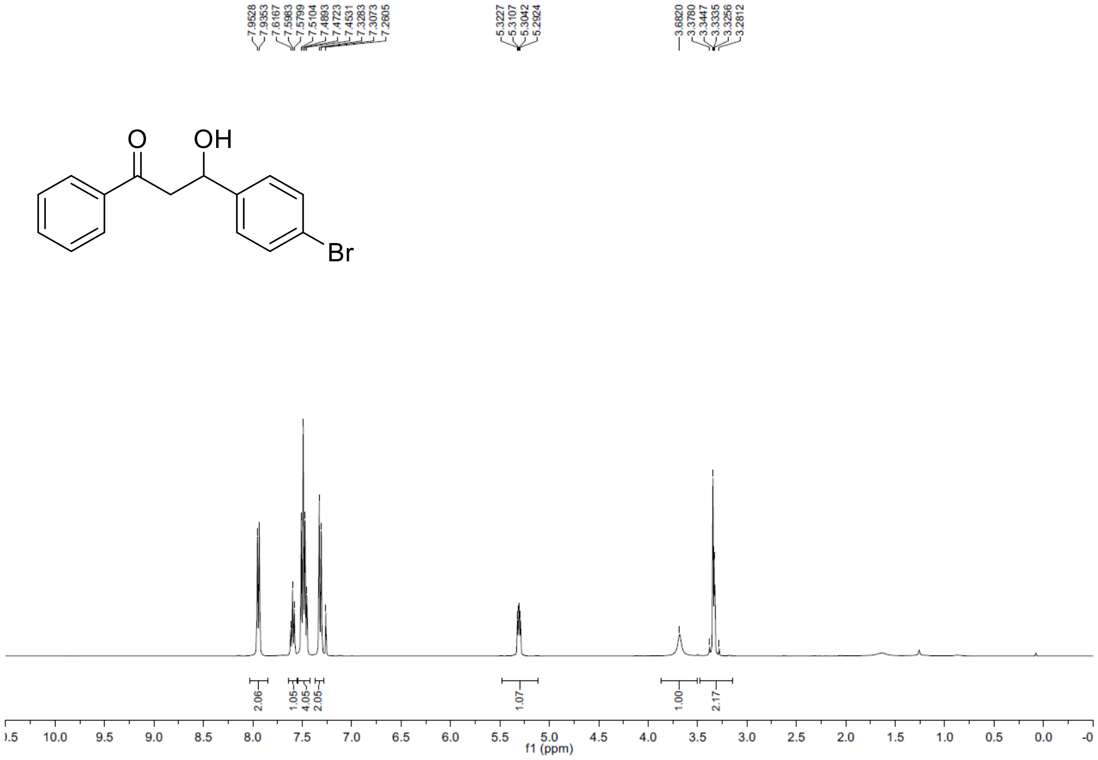
O
OH
Br
f1 (ppm)

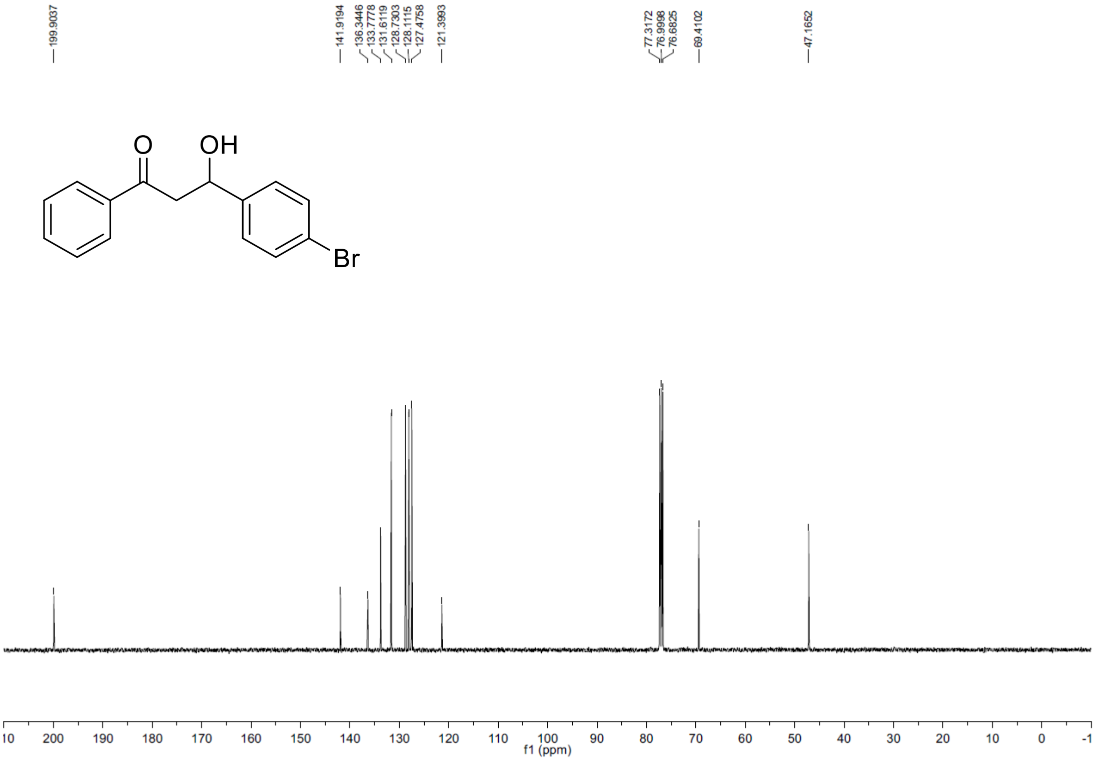
O
OH
Br
f1 (ppm)

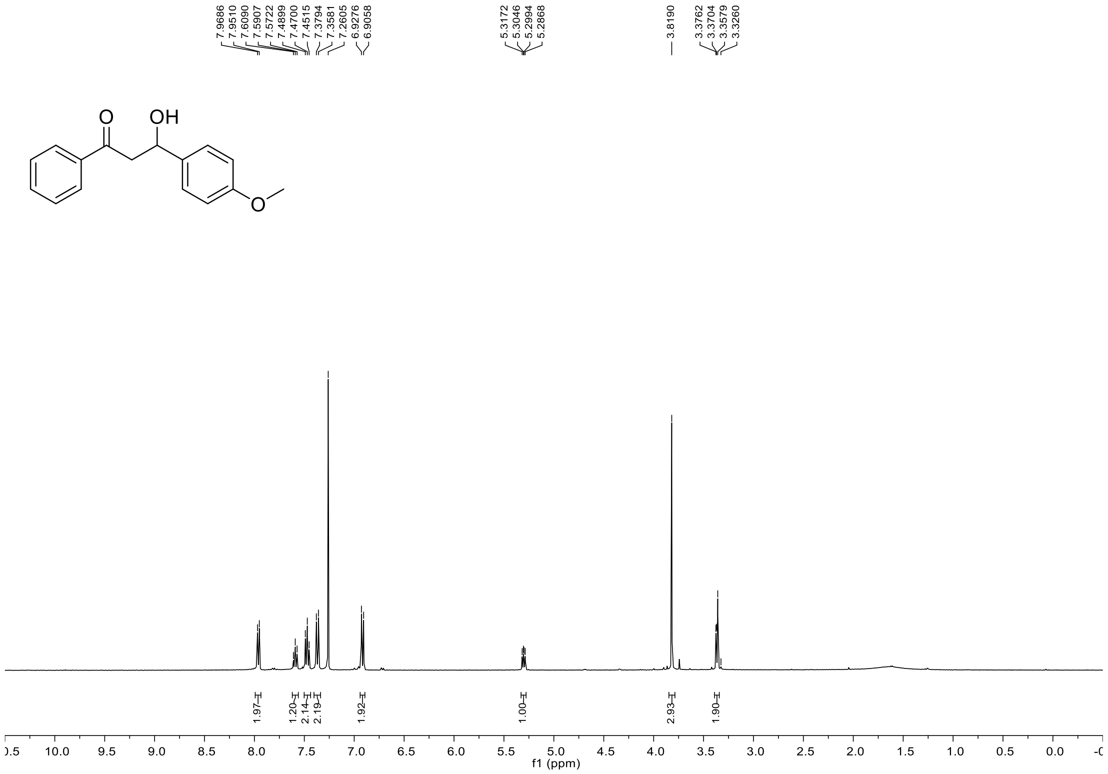
7.9686
7.9510
7.6090
7.5907
7.5722
7.4899
7.4700
7.4515
7.3794
7.3581
7.2605
6.9276
6.9058
5.3172
5.3046
5.2994
5.2868
3.8190
3.3762
3.3704
3.3579
3.3260
O
OH
O
1.97
1.20
2.14
2.19
1.92
1.00
2.93
1.90
f1 (ppm)

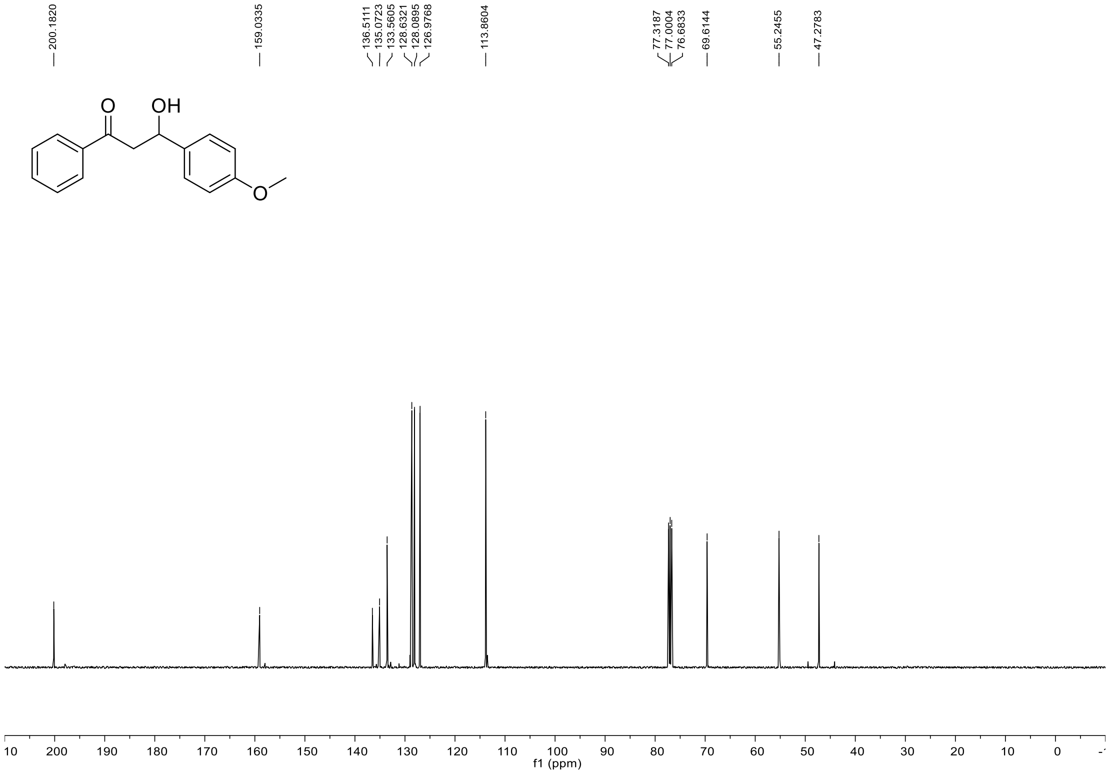
200.1820
159.0335
136.5111
135.0723
133.5605
128.6321
128.0895
126.9768
113.8604
77.3187
77.0004
76.6833
69.6144
55.2455
47.2783
O
OH
O
f1 (ppm)

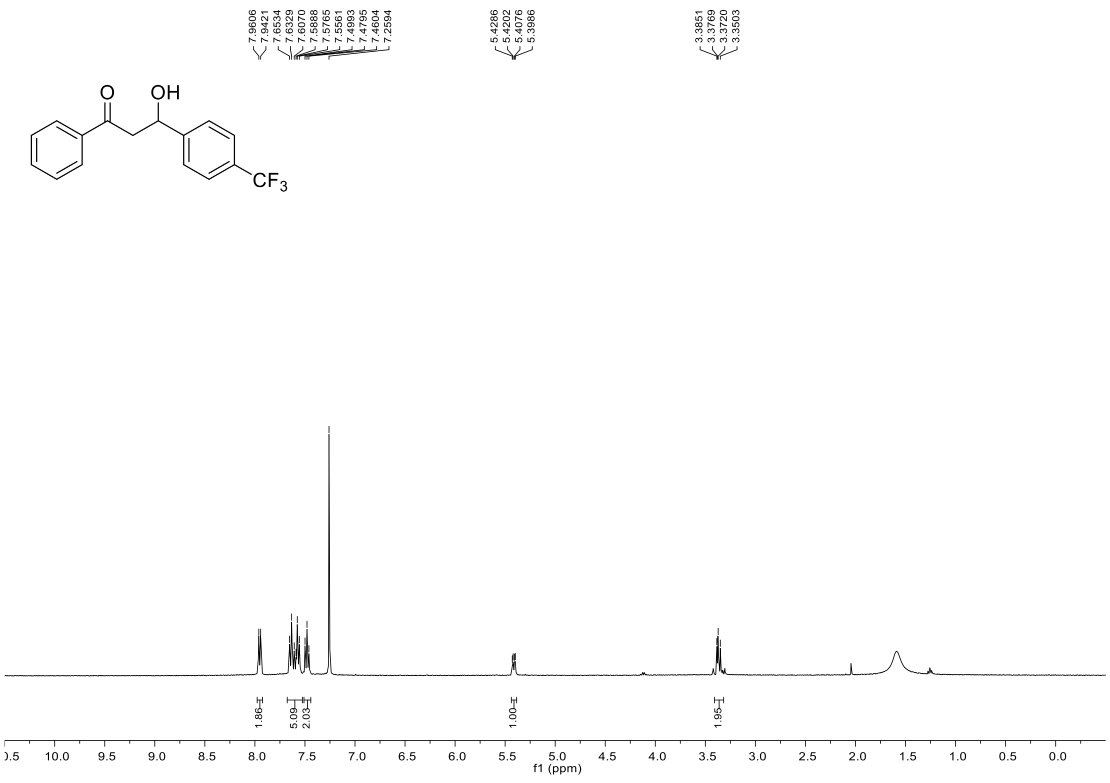
7.9606
7.9421
7.6534
7.6329
7.6070
7.5888
7.5765
7.5561
7.4993
7.4795
7.4604
7.2594
5.4286
5.4202
5.4076
5.3986
3.3851
3.3769
3.3720
3.3503
O
OH
CF3
1.86
5.09
2.03
1.00
1.95
f1 (ppm)

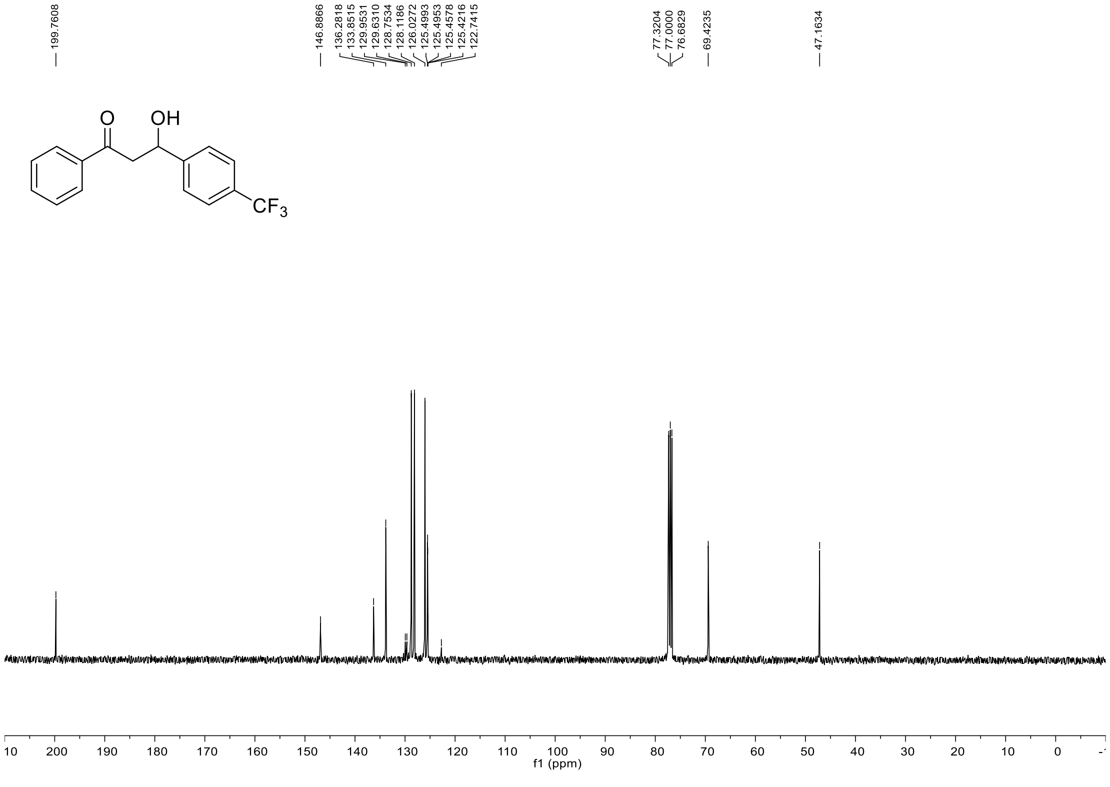
199.7608
146.8866
136.2818
133.8515
129.9531
129.6310
128.7534
128.1186
126.0272
125.4993
125.4953
125.4578
125.4216
122.7415
77.3204
77.0000
76.6829
69.4235
47.1634
O
OH
CF3
f1 (ppm)

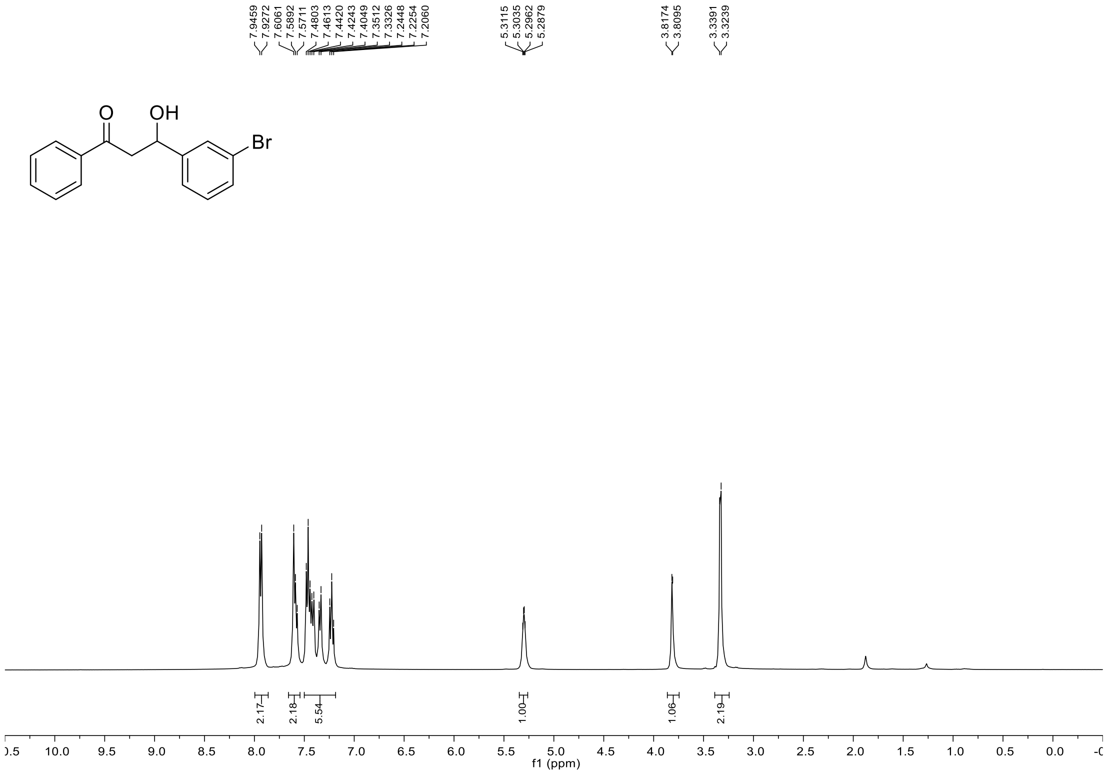
7.9459
7.9272
7.6061
7.5892
7.5711
7.4803
7.4613
7.4420
7.4243
7.4049
7.3512
7.3326
7.2448
7.2254
7.2060
5.3115
5.3035
5.2962
5.2879
3.8174
3.8095
3.3391
3.3239
O
OH
Br
2.17
2.18
5.54
1.00
1.06
2.19
f1 (ppm)

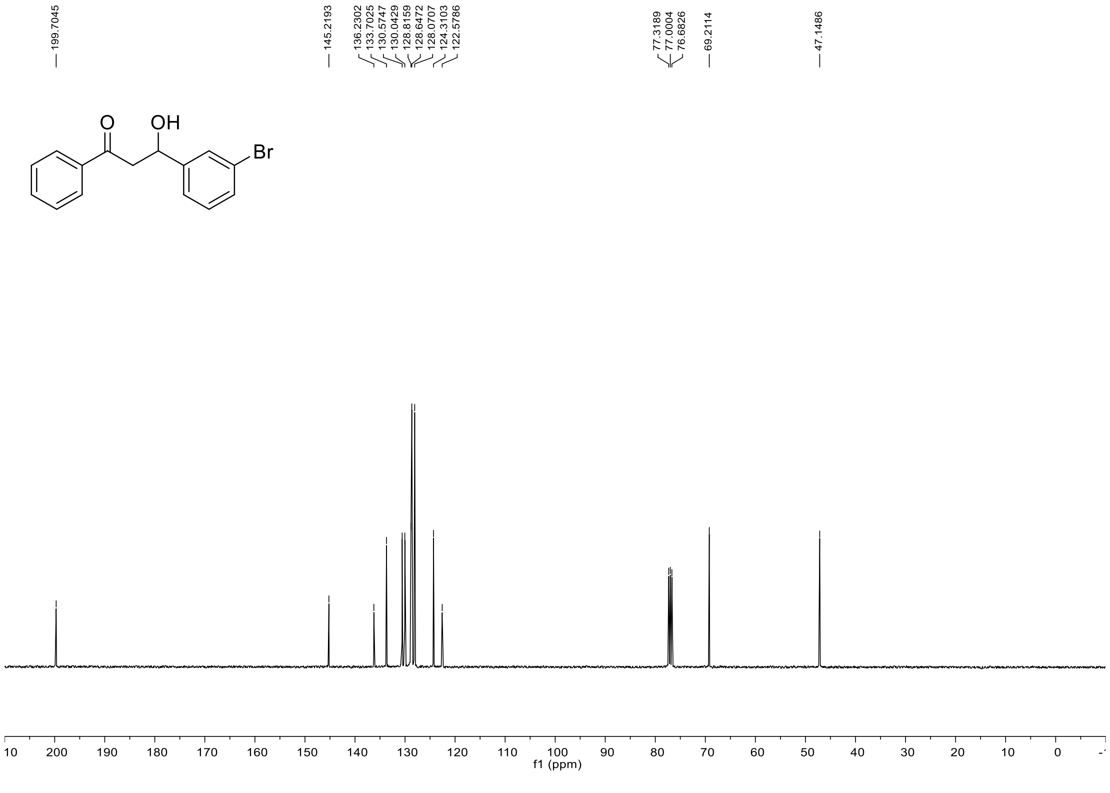
199.7045
145.2193
136.2302
133.7025
130.5747
130.0429
128.8159
128.6472
128.0707
124.3103
122.5786
77.3189
77.0004
76.6826
69.2114
47.1486
O
OH
Br
f1 (ppm)

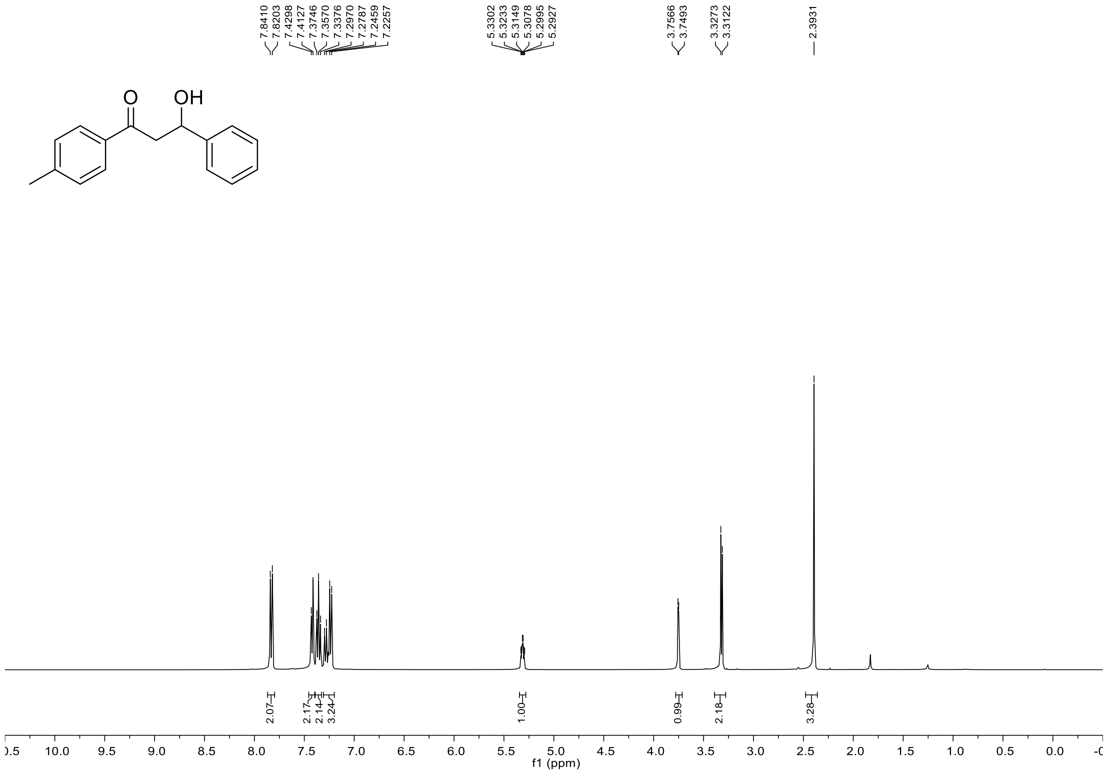
7.8410
7.8203
7.4298
7.4127
7.3746
7.3570
7.3376
7.2970
7.2787
7.2459
7.2257
5.3302
5.3233
5.3149
5.3078
5.2995
5.2927
3.7566
3.7493
3.3273
3.3122
2.3931
O
OH
2.07
2.17
2.14
3.24
1.00
0.99
2.18
3.28
f1 (ppm)

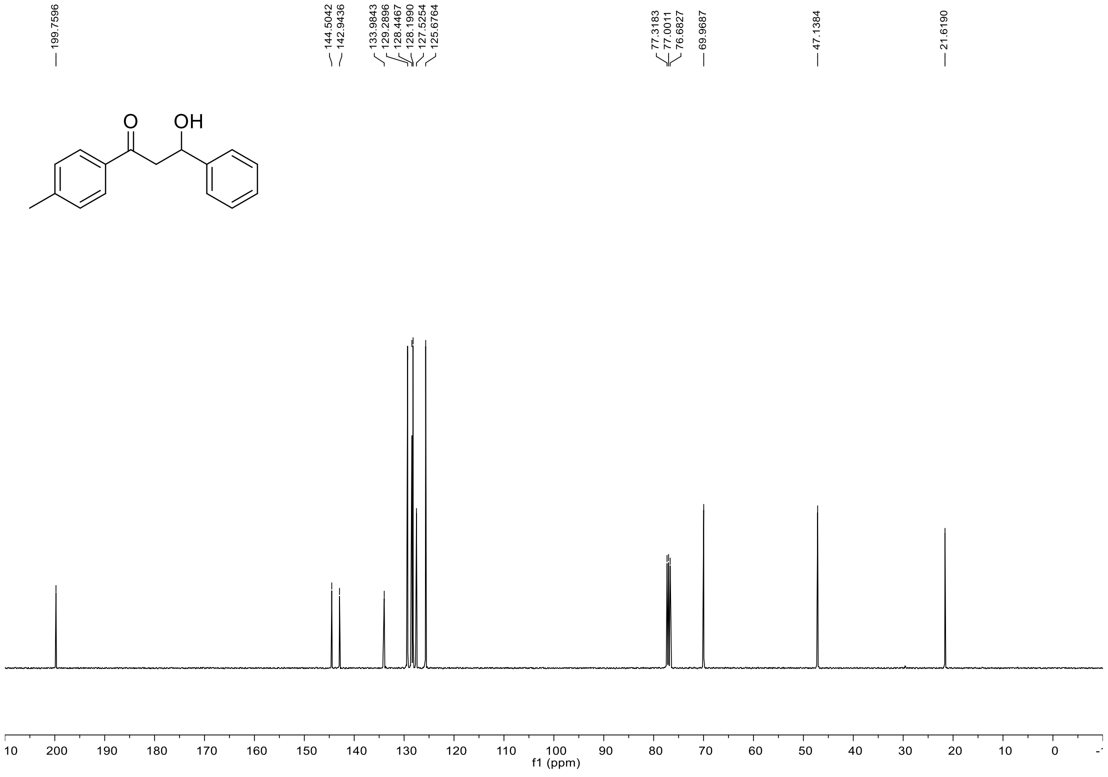
199.7596
144.5042
142.9436
133.9843
129.2896
128.4467
128.1990
127.5254
125.6764
77.3183
77.0011
76.6827
69.9687
47.1384
21.6190
O
OH
f1 (ppm)

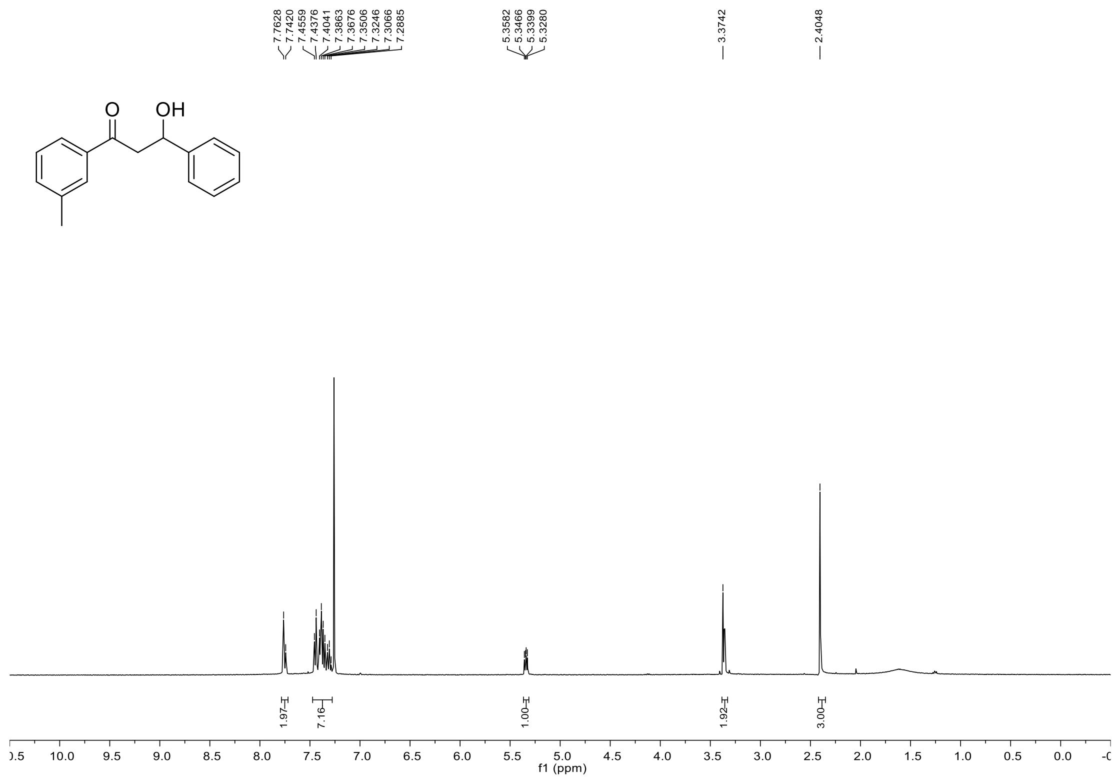
7.7628
7.7420
7.4559
7.4376
7.4041
7.3863
7.3676
7.3506
7.3246
7.3066
7.2885
5.3582
5.3466
5.3399
5.3280
3.3742
2.4048
O
OH
1.97
7.16
1.00
1.92
3.00
f1 (ppm)

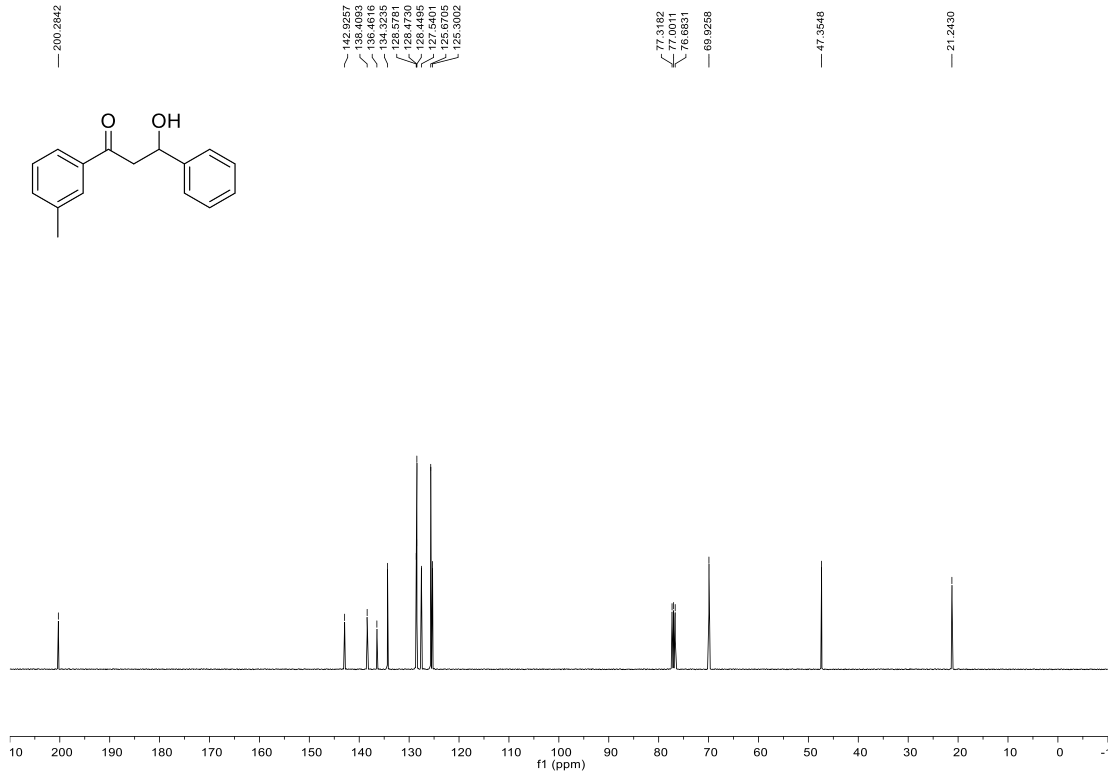
200.2842
142.9257
138.4093
136.4616
134.3235
128.5781
128.4730
128.4495
127.5401
125.6705
125.3002
77.3182
77.0011
76.6831
69.9258
47.3548
21.2430
O
OH
f1 (ppm)

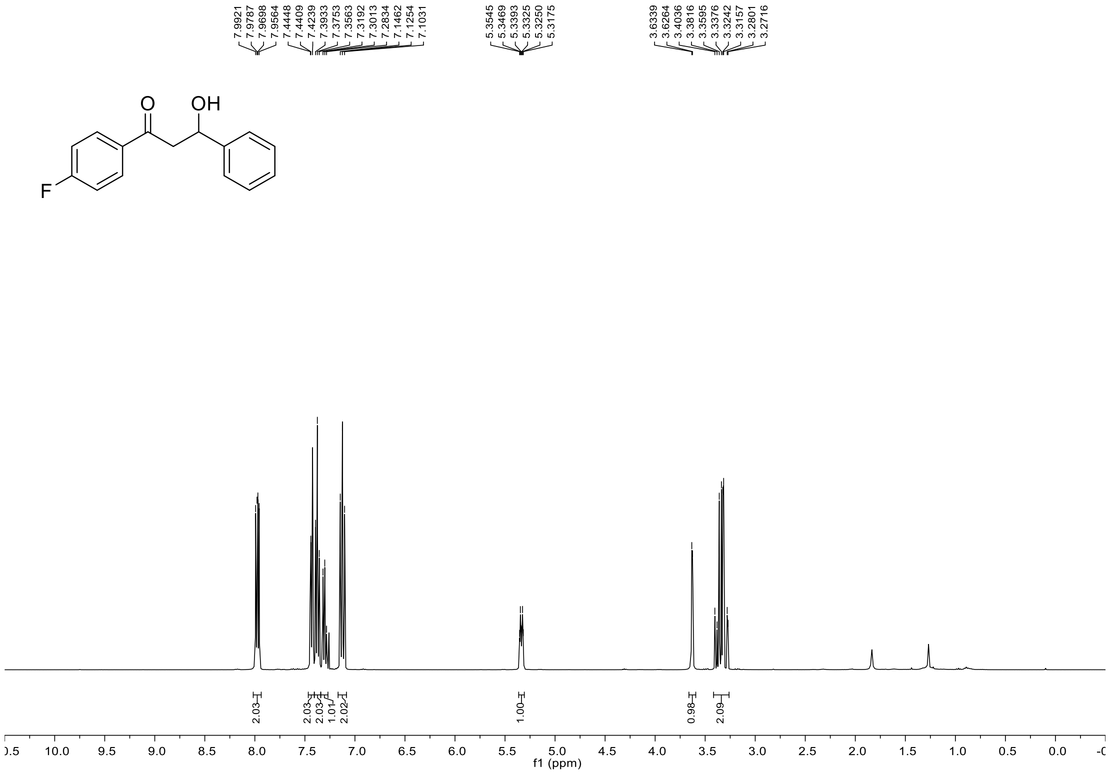
7.9921
7.9787
7.9698
7.9564
7.4448
7.4409
7.4239
7.3933
7.3753
7.3563
7.3192
7.3013
7.2834
7.1462
7.1254
7.1031
5.3545
5.3469
5.3393
5.3325
5.3250
5.3175
3.6339
3.6264
3.4036
3.3816
3.3595
3.3376
3.3242
3.3157
3.2801
3.2716
O
OH
F
2.03
2.03
2.03
1.01
2.02
1.00
0.98
2.09
f1 (ppm)

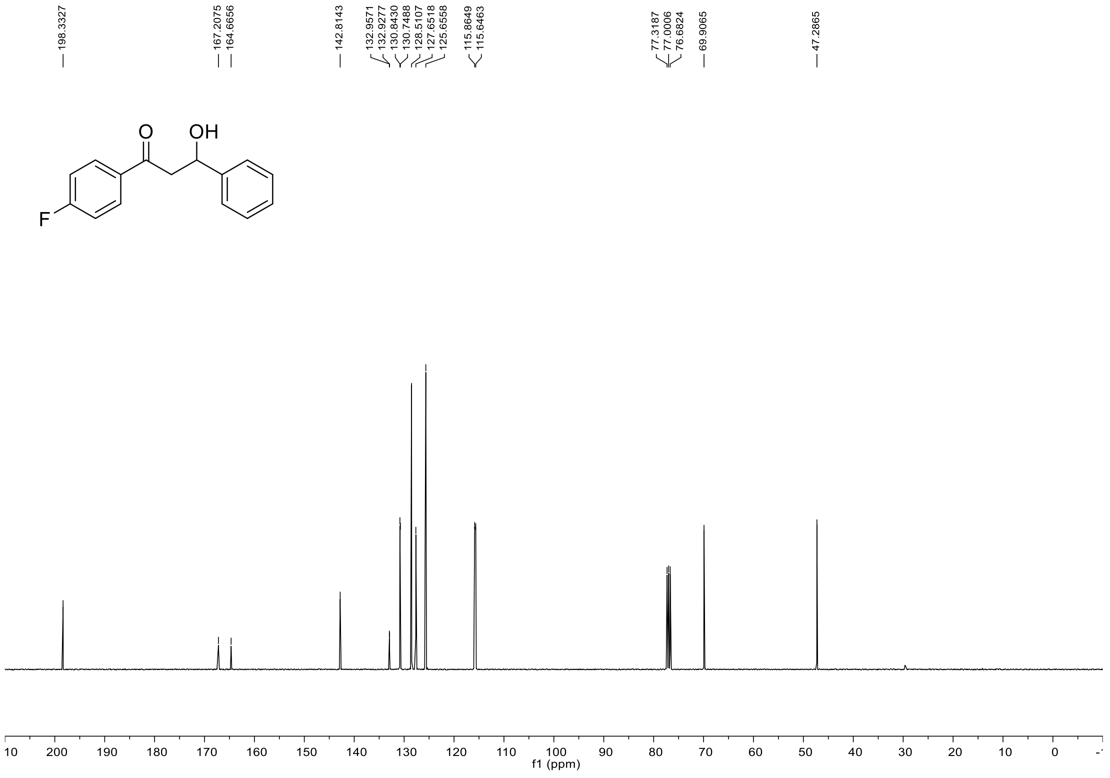
198.3327
167.2075
164.6656
142.8143
132.9571
132.9277
130.8430
130.7488
128.5107
127.6518
125.6558
115.8649
115.6463
77.3187
77.0006
76.6824
69.9065
47.2865
O
OH
F
f1 (ppm)

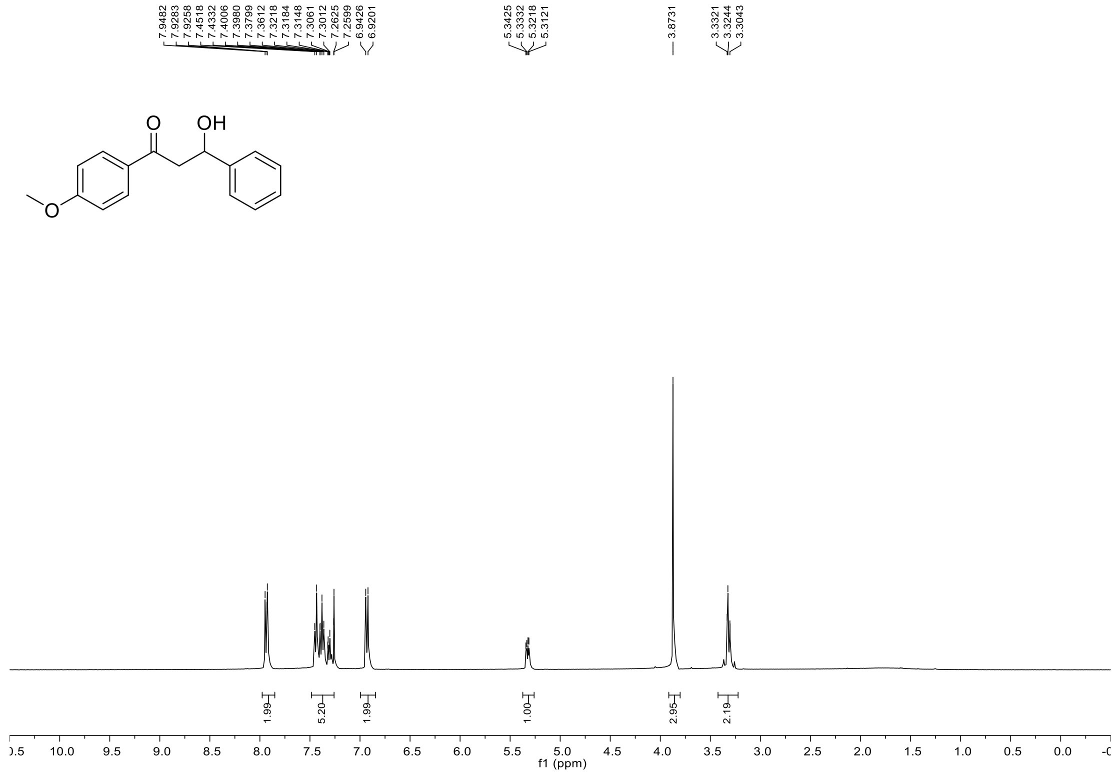
7.9482
7.9283
7.9258
7.4518
7.4332
7.4006
7.3980
7.3799
7.3612
7.3218
7.3184
7.3148
7.3061
7.3012
7.2625
7.2599
6.9426
6.9201
5.3425
5.3332
5.3218
5.3121
3.8731
3.3321
3.3244
3.3043
O
OH
O
1.99
5.20
1.99
1.00
2.95
2.19
f1 (ppm)

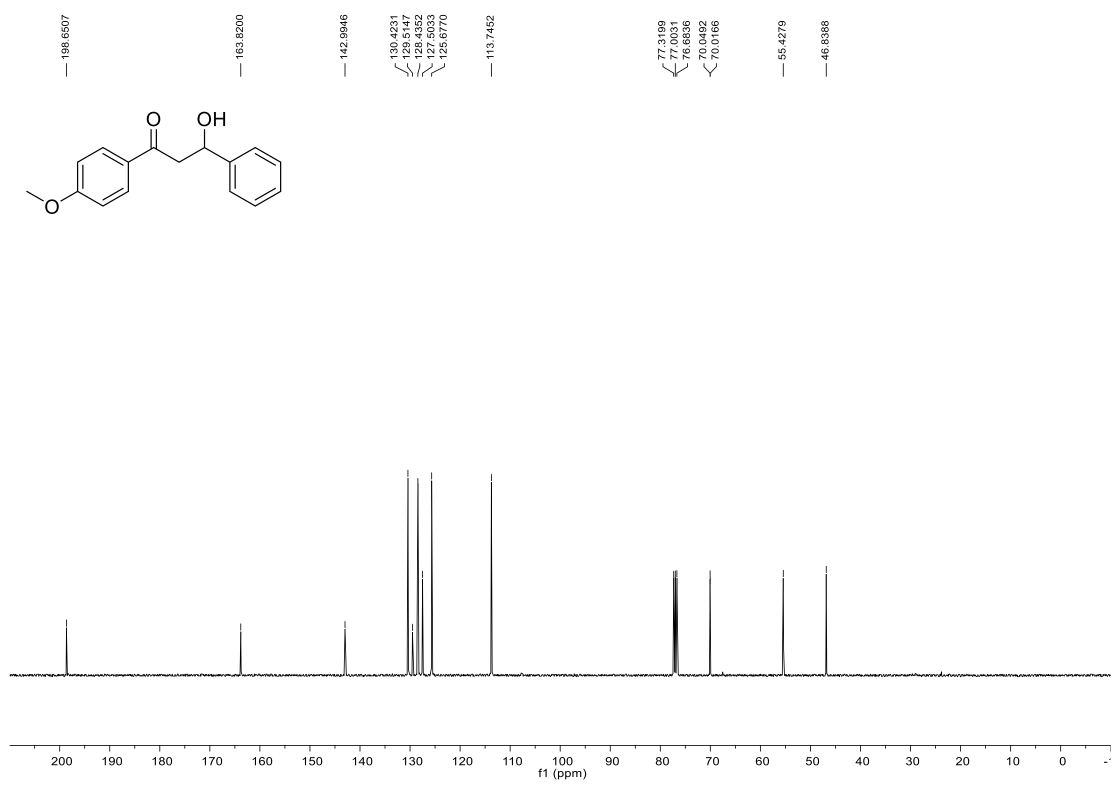
198.6507
163.8200
142.9946
130.4231
129.5147
128.4352
127.5033
125.6770
113.7452
77.3199
77.0031
76.6836
70.0492
70.0166
55.4279
46.8388
O
OH
O
f1 (ppm)

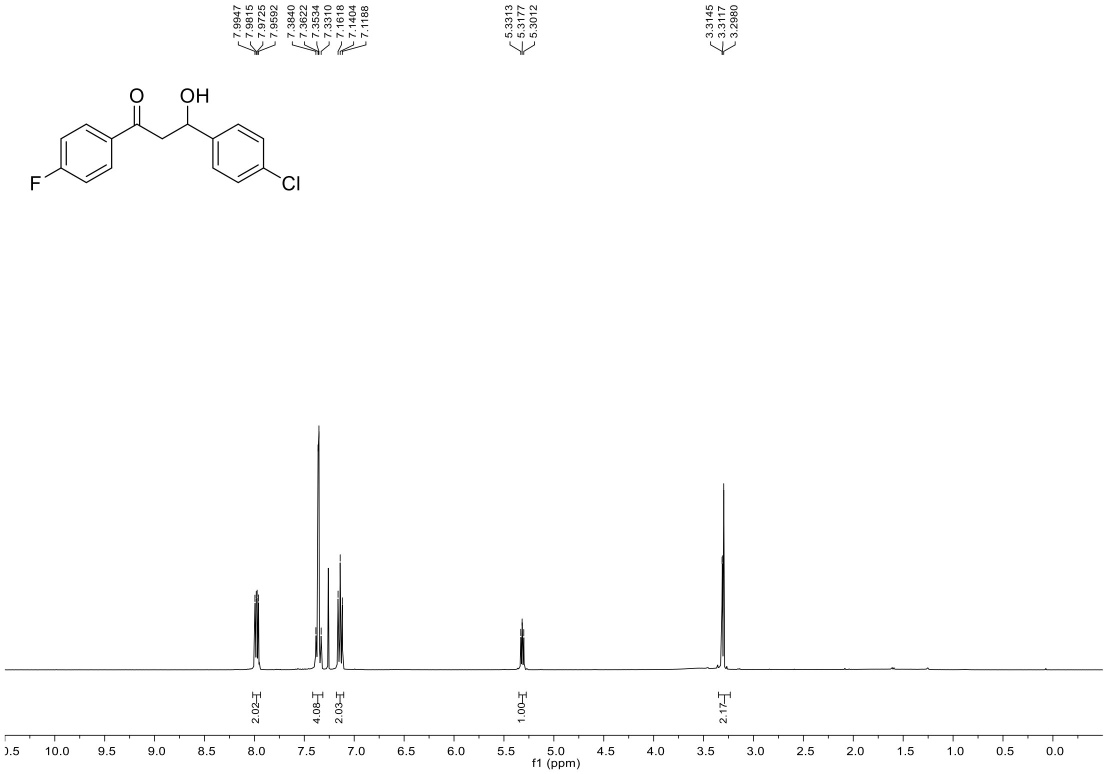
7.9947
7.9815
7.9725
7.9592
7.3840
7.3622
7.3534
7.3310
7.1618
7.1404
7.1188
5.3313
5.3177
5.3012
3.3145
3.3117
3.2980
O
OH
F
Cl
2.02
4.08
2.03
1.00
2.17
f1 (ppm)

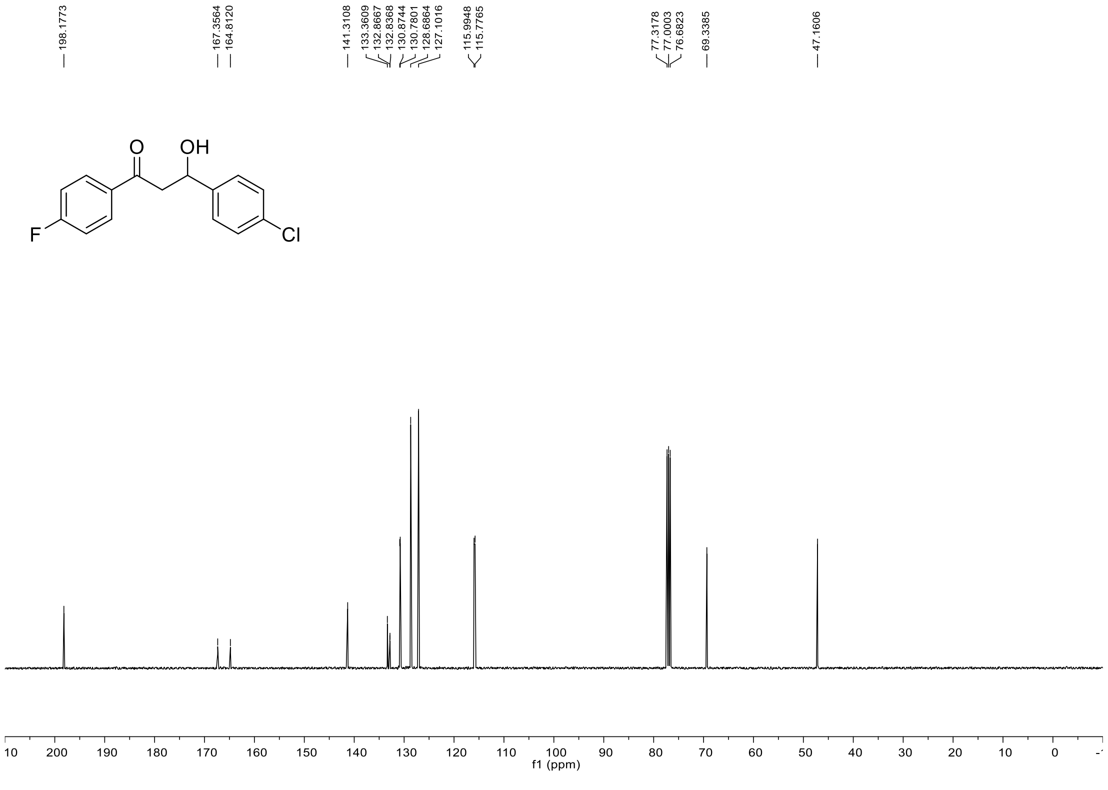
198.1773
167.3564
164.8120
141.3108
133.3609
132.8667
132.8368
130.8744
130.7801
128.6864
127.1016
115.9948
115.7765
77.3178
77.0003
76.6823
69.3385
47.1606
O
OH
F
Cl
f1 (ppm)

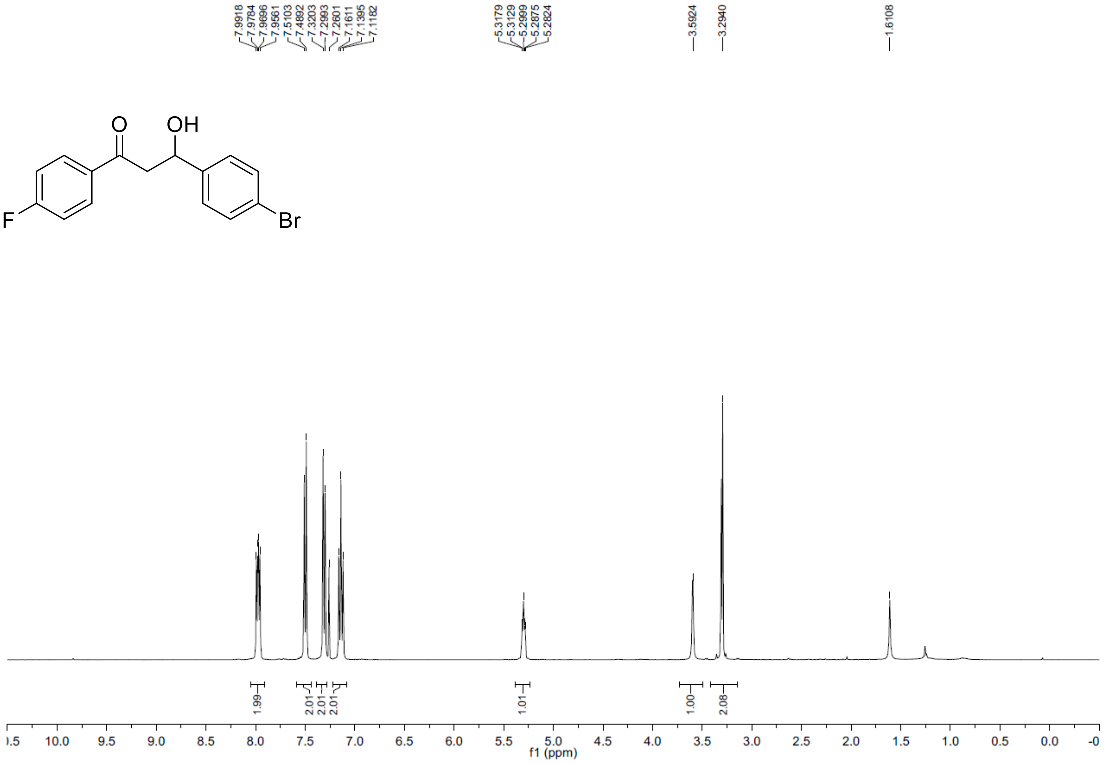
7.9918
7.9784
7.9696
7.9561
7.5103
7.4892
7.3203
7.2993
7.2601
7.1611
7.1395
7.1182
5.3179
5.3129
5.2999
5.2875
5.2824
3.5924
3.2940
1.6108
O
OH
F
Br
1.99
2.01
2.01
2.01
1.01
1.00
2.08
f1 (ppm)

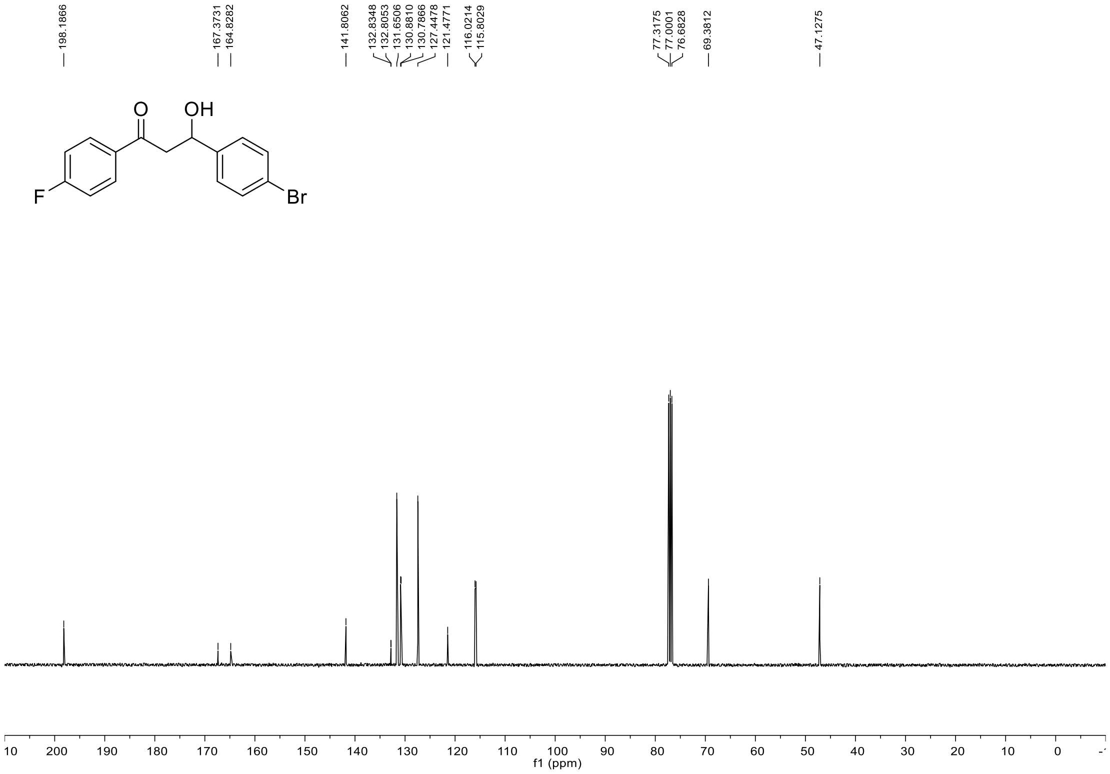
198.1866
167.3731
164.8282
141.8062
132.8348
132.8053
131.6506
130.8810
130.7866
127.4478
121.4771
116.0214
115.8029
77.3175
77.0001
76.6828
69.3812
47.1275
O
OH
F
Br
f1 (ppm)

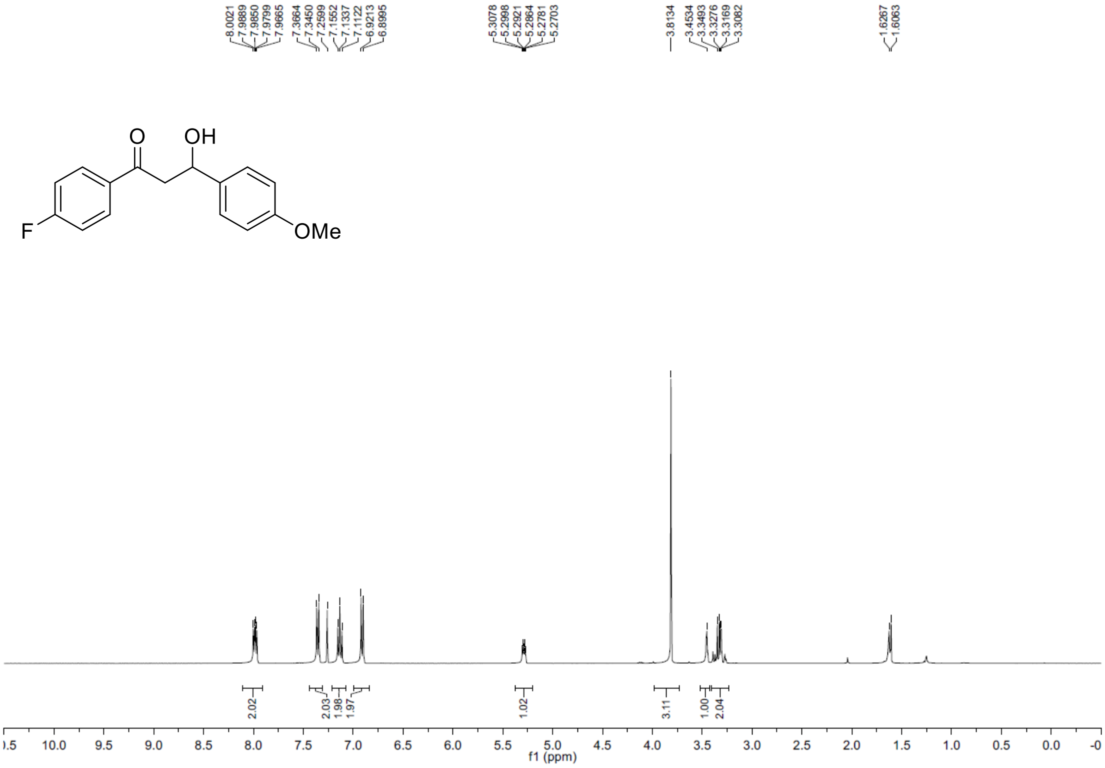
8.0021
7.9889
7.9850
7.9799
7.9665
7.3664
7.3450
7.2599
7.1552
7.1337
7.1122
6.9213
6.8995
5.3078
5.2998
5.2921
5.2864
5.2781
5.2703
3.8134
3.4534
3.3493
3.3276
3.3169
3.3082
1.6267
1.6063
O
OH
F
OMe
2.02
2.03
1.98
1.97
1.02
3.11
1.00
2.04
f1 (ppm)

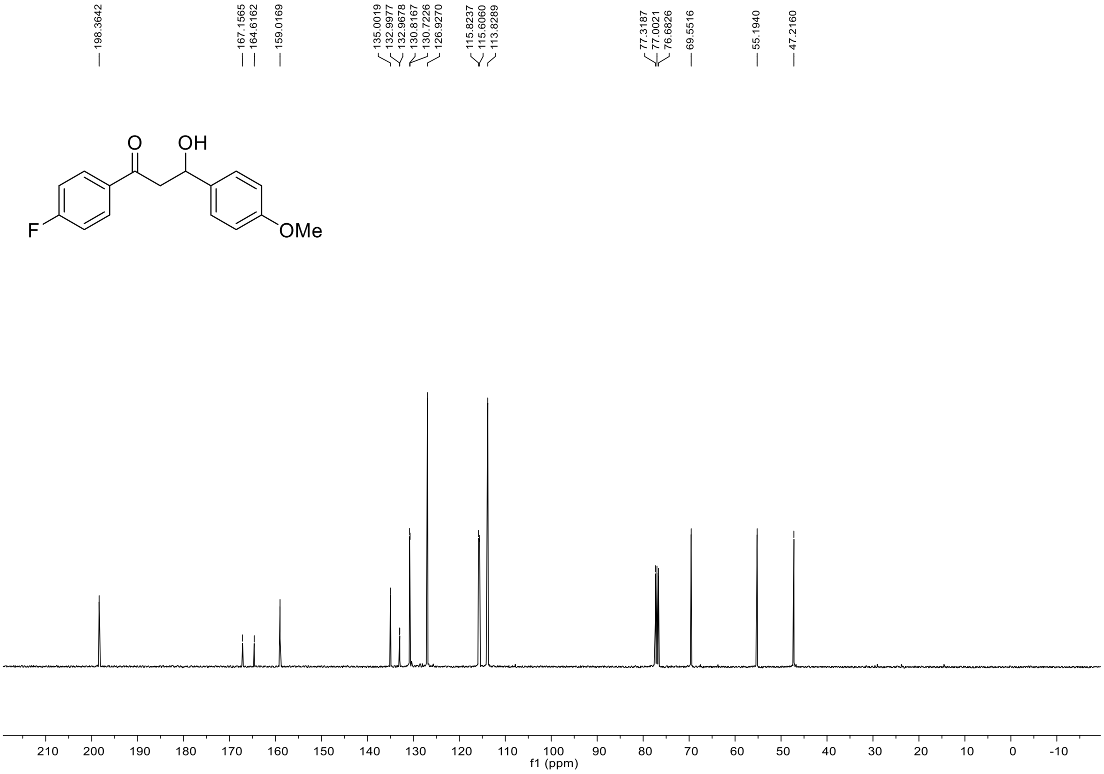
198.3642
167.1565
164.6162
159.0169
135.0019
132.9977
132.9678
130.8167
130.7226
126.9270
115.8237
115.6060
113.8289
77.3187
77.0021
76.6826
69.5516
55.1940
47.2160
O
OH
F
OMe
f1 (ppm)

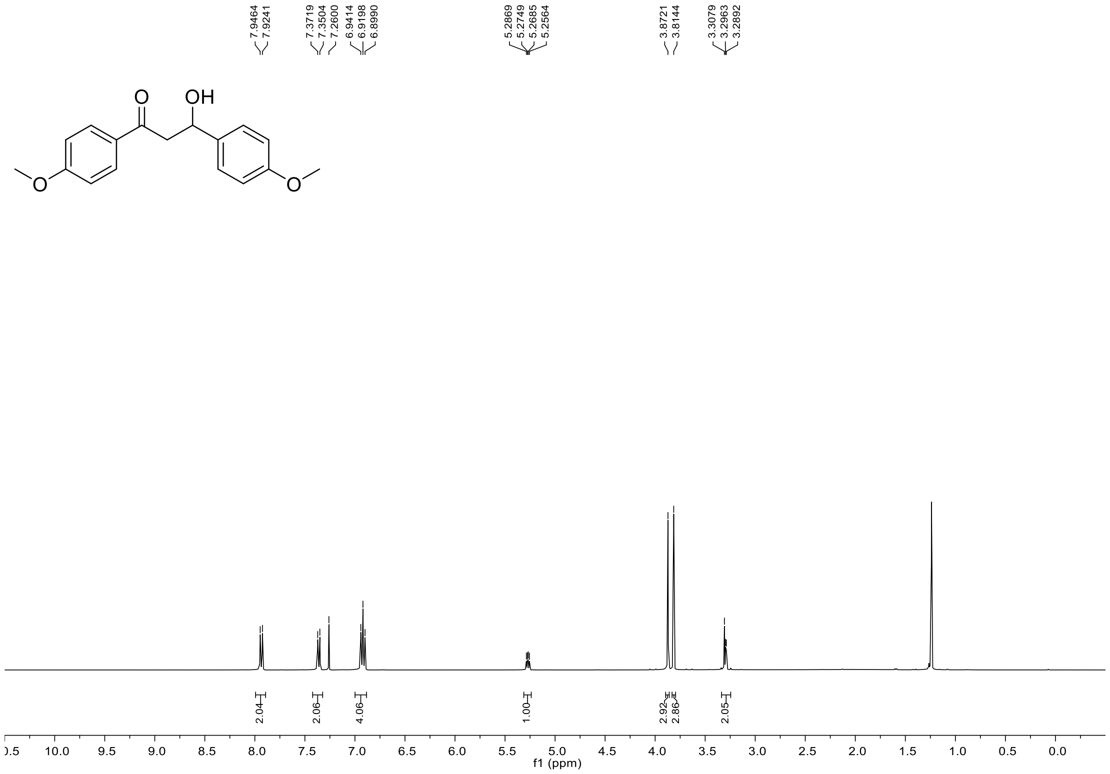
7.9464
7.9241
7.3719
7.3504
7.2600
6.9414
6.9198
6.8990
5.2869
5.2749
5.2685
5.2564
3.8721
3.8144
3.3079
3.2963
3.2892
O
OH
O
O
2.04
2.06
4.06
1.00
2.92
2.86
2.05
10.0 9.5 9.0 8.5 8.0 7.5 7.0 6.5 6.0 5.5 5.0 4.5 4.0 3.5 3.0 2.5 2.0 1.5 1.0 0.5 0.0
f1 (ppm)

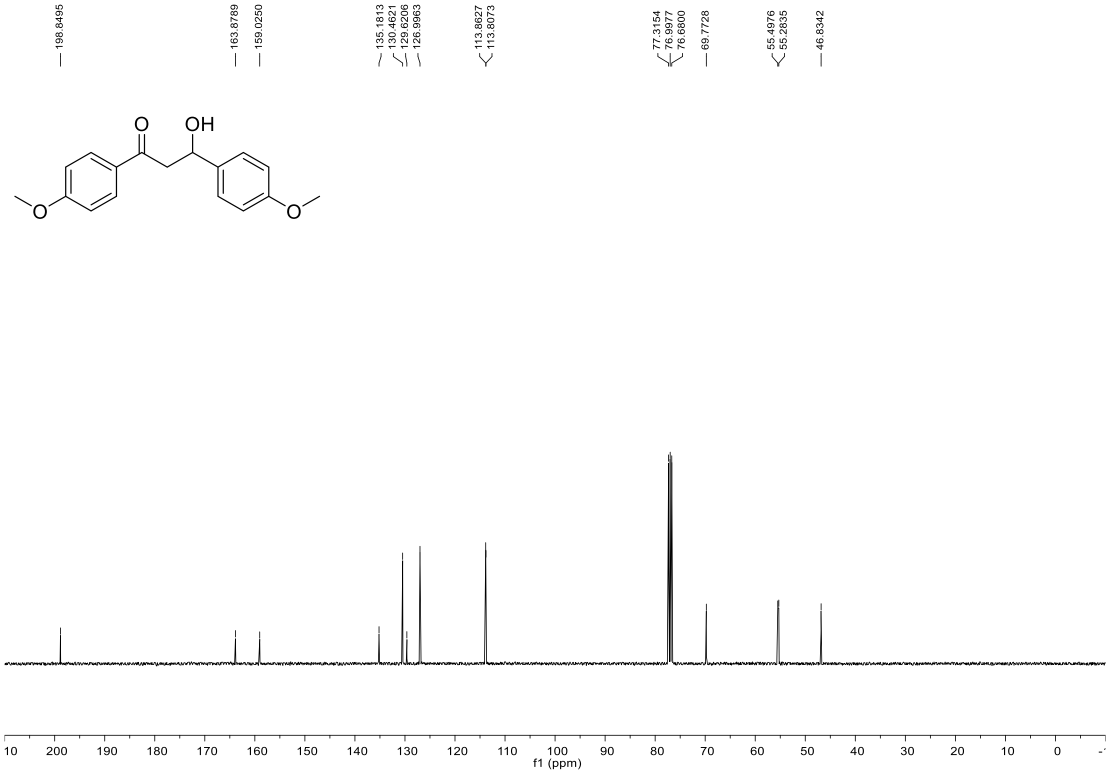
198.8495
163.8789
159.0250
135.1813
130.4621
129.6206
126.9963
113.8627
113.8073
77.3154
76.9977
76.6800
69.7728
55.4976
55.2835
46.8342
O
OH
O
O
200 190 180 170 160 150 140 130 120 110 100 90 80 70 60 50 40 30 20 10 0
f1 (ppm)

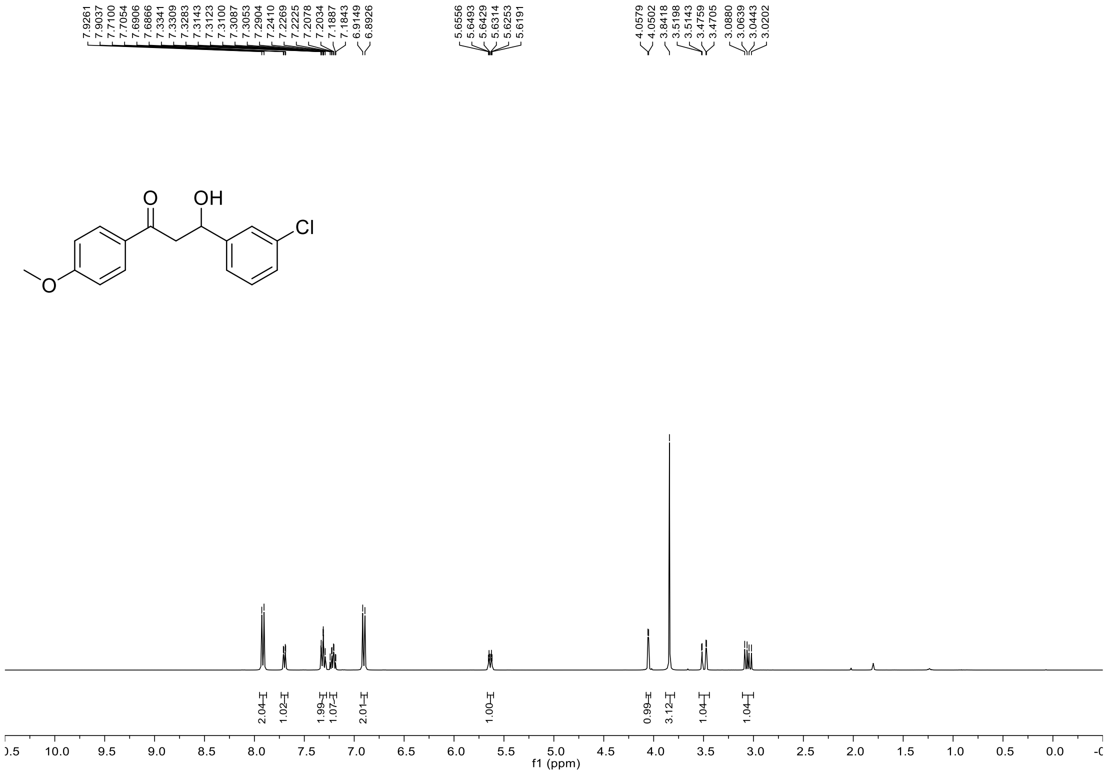
7.9261
7.9037
7.7100
7.7054
7.6906
7.6866
7.3341
7.3309
7.3283
7.3143
7.3123
7.3100
7.3087
7.3053
7.2904
7.2410
7.2269
7.2225
7.2078
7.2034
7.1887
7.1843
6.9149
6.8926
5.6556
5.6493
5.6429
5.6314
5.6253
5.6191
4.0579
4.0502
3.8418
3.5198
3.5143
3.4759
3.4705
3.0880
3.0639
3.0443
3.0202
O
OH
Cl
O
2.04
1.02
1.99
1.07
2.01
1.00
0.99
3.12
1.04
1.04
10.0
9.5
9.0
8.5
8.0
7.5
7.0
6.5
6.0
5.5
5.0
4.5
4.0
3.5
3.0
2.5
2.0
1.5
1.0
0.5
0.0
f1 (ppm)

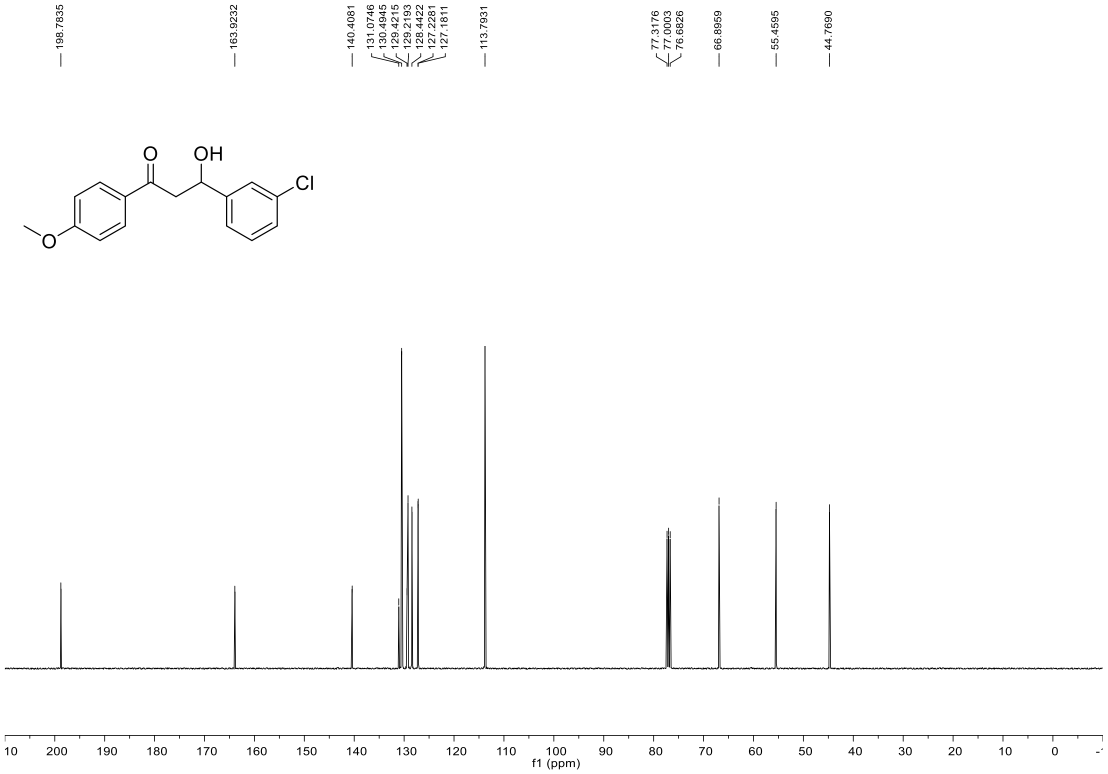
198.7835
163.9232
140.4081
131.0746
130.4945
129.4215
129.2193
128.4422
127.2281
127.1811
113.7931
77.3176
77.0003
76.6826
66.8959
55.4595
44.7690
O
OH
Cl
O
200
190
180
170
160
150
140
130
120
110
100
90
80
70
60
50
40
30
20
10
0
f1 (ppm)

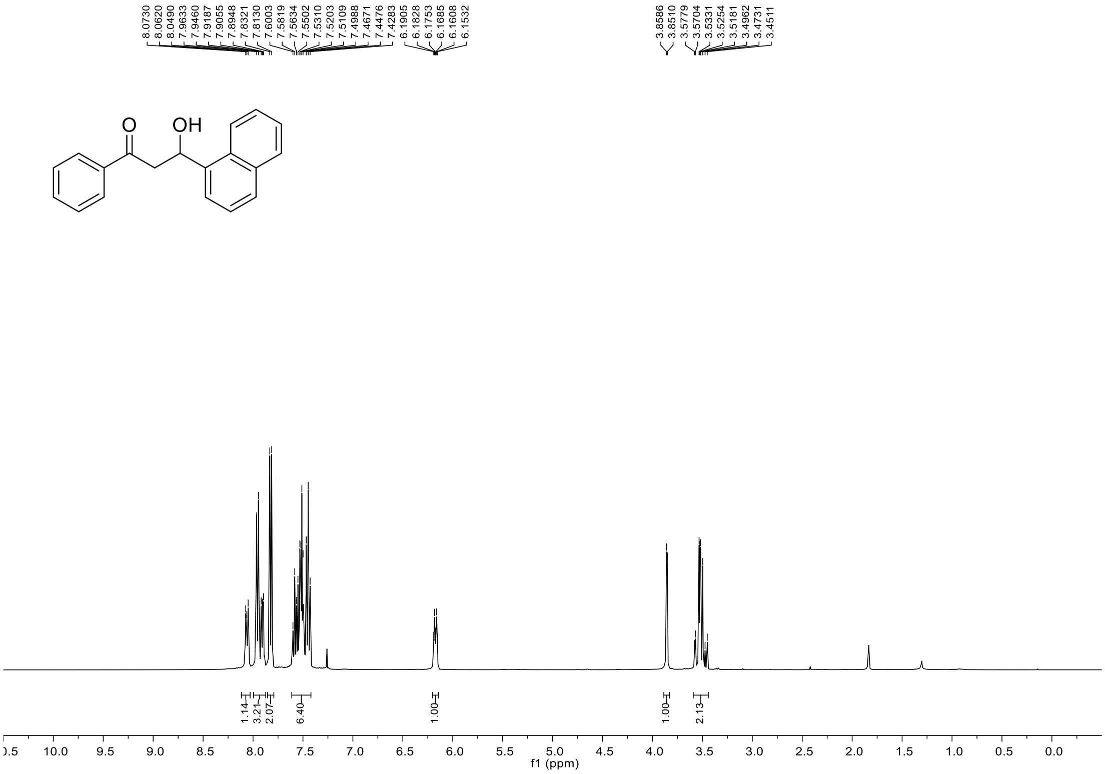
8.0730
8.0620
8.0490
7.9833
7.9460
7.9187
7.9055
7.8948
7.8321
7.8130
7.6003
7.5819
7.5634
7.5502
7.5310
7.5203
7.5109
7.4988
7.4671
7.4476
7.4283
6.1905
6.1828
6.1753
6.1685
6.1608
6.1532
3.8586
3.8510
3.5779
3.5704
3.5331
3.5254
3.5181
3.4962
3.4731
3.4511
O
OH
1.14
3.21
2.07
6.40
1.00
1.00
2.13
f1 (ppm)

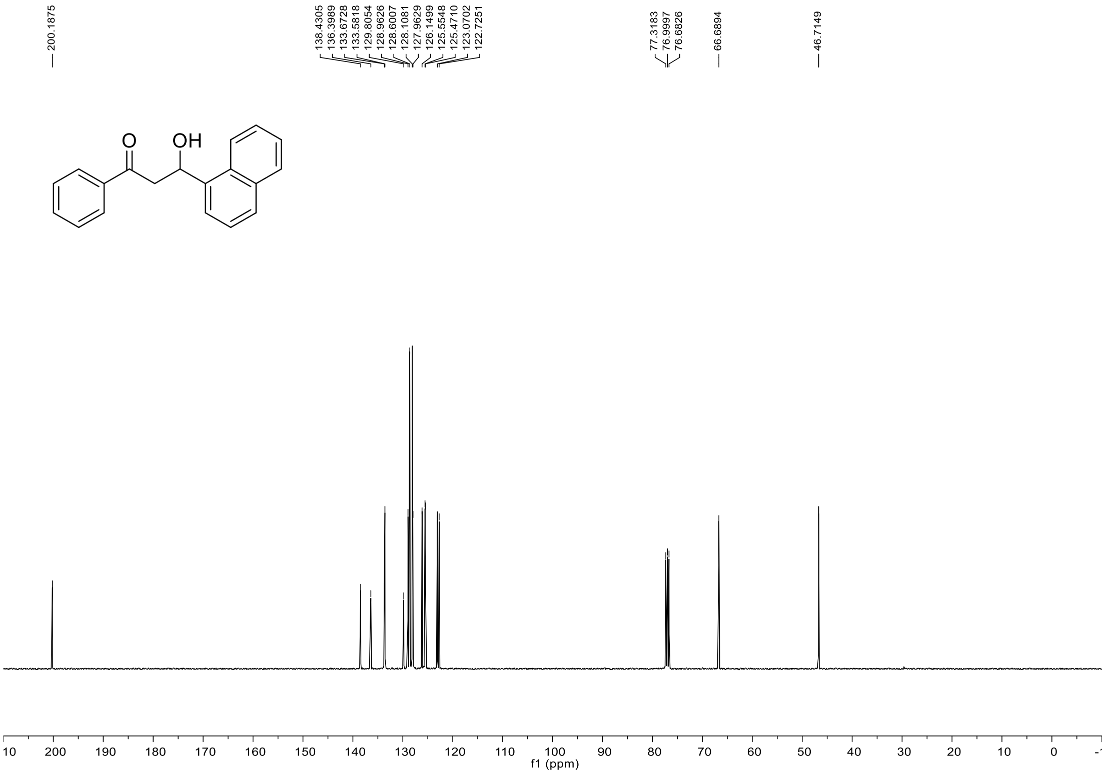
200.1875
138.4305
136.3989
133.6728
133.5818
129.8054
128.9626
128.6007
128.1081
127.9629
126.1499
125.5548
125.4710
123.0702
122.7251
77.3183
76.9997
76.6826
66.6894
46.7149
O
OH
f1 (ppm)

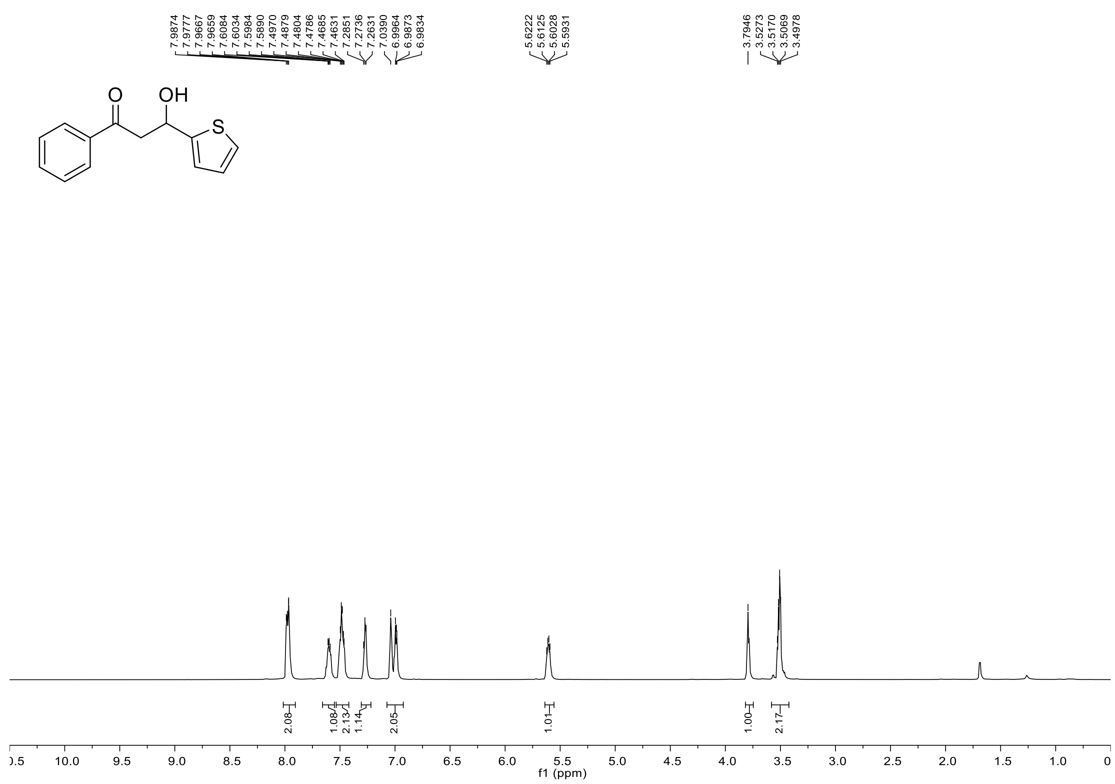
7.9874
7.9777
7.9667
7.9659
7.6084
7.6034
7.5984
7.5890
7.4970
7.4879
7.4804
7.4786
7.4685
7.4631
7.2851
7.2736
7.2631
7.0390
6.9964
6.9873
6.9834
5.6222
5.6125
5.6028
5.5931
3.7946
3.5273
3.5170
3.5069
3.4978
O
OH
S
2.08
1.08
2.13
1.14
2.05
1.01
1.00
2.17
10.0 9.5 9.0 8.5 8.0 7.5 7.0 6.5 6.0 5.5 5.0 4.5 4.0 3.5 3.0 2.5 2.0 1.5 1.0
f1 (ppm)

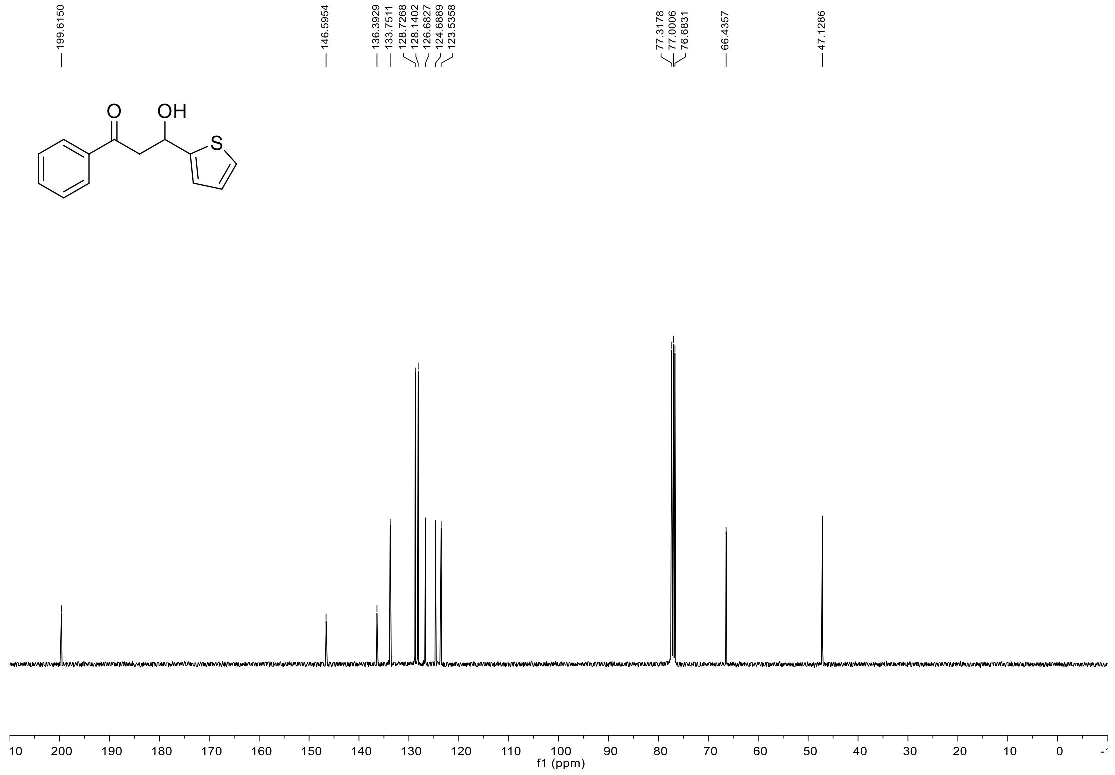
199.6150
146.5954
136.3929
133.7511
128.7268
128.1402
126.6827
124.6889
123.5358
77.3178
77.0006
76.6831
66.4357
47.1286
O
OH
S
200 190 180 170 160 150 140 130 120 110 100 90 80 70 60 50 40 30 20 10 0
f1 (ppm)

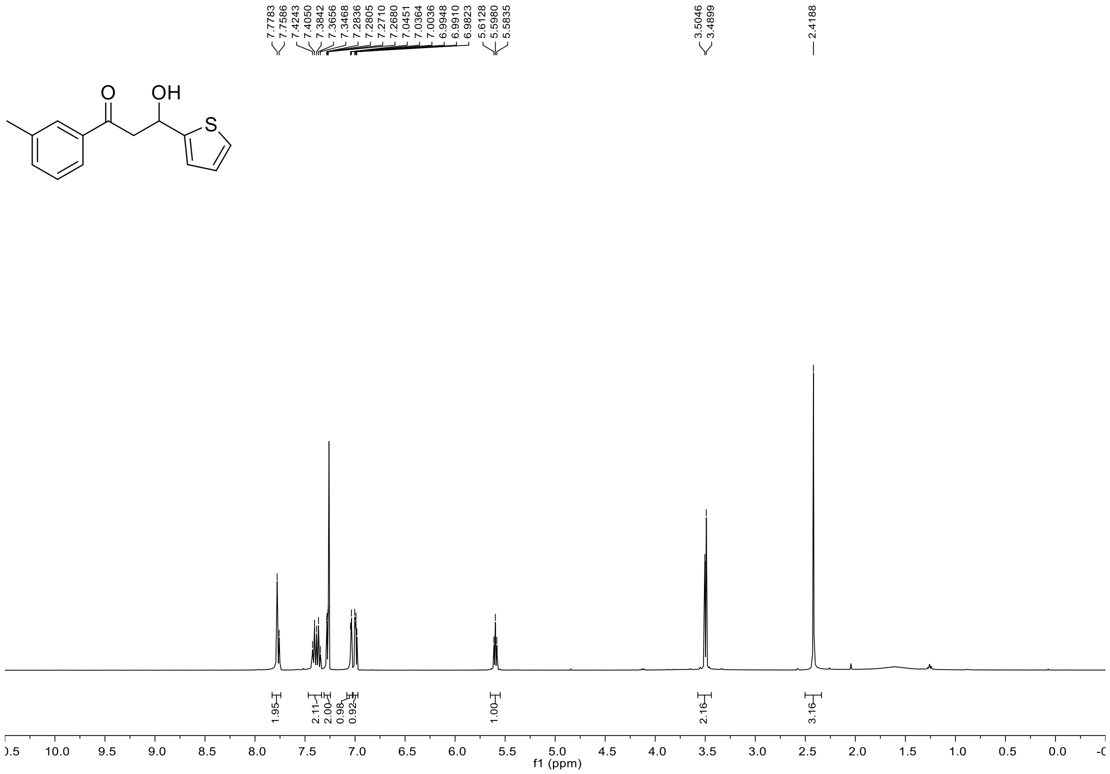
7.7783
7.7586
7.4243
7.4050
7.3842
7.3656
7.3468
7.2836
7.2805
7.2710
7.2680
7.0451
7.0364
7.0036
6.9948
6.9910
6.9823
5.6128
5.5980
5.5835
3.5046
3.4899
2.4188
O
OH
S
1.95
2.11
2.00
0.98
0.92
1.00
2.16
3.16
f1 (ppm)

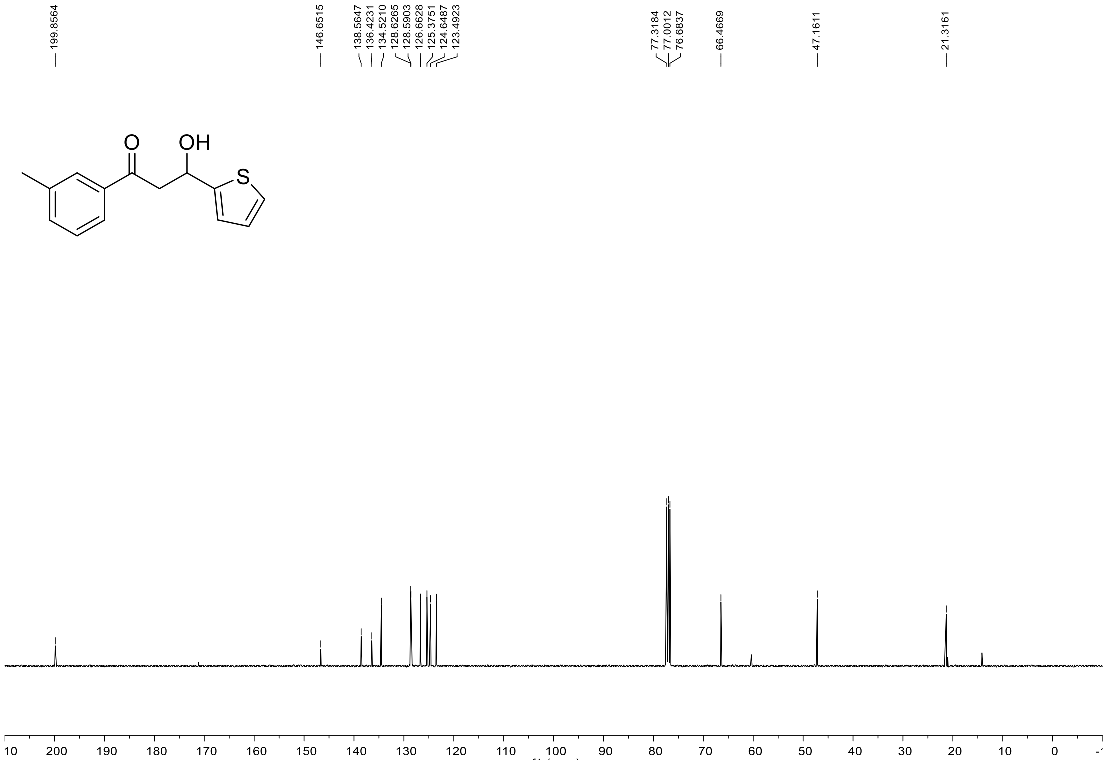
199.8564
146.6515
138.5647
136.4231
134.5210
128.6265
128.5903
126.6628
125.3751
124.6487
123.4923
77.3184
77.0012
76.6837
66.4669
47.1611
21.3161
O
OH
S
f1 (ppm)

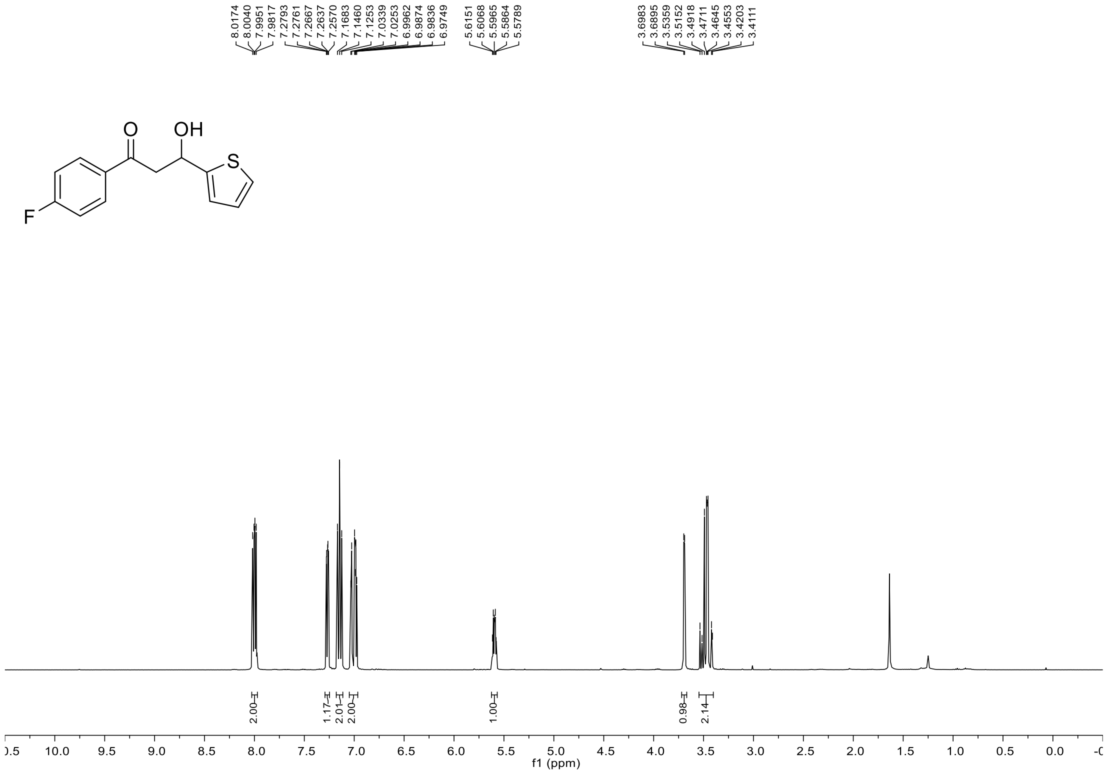
8.0174
8.0040
7.9951
7.9817
7.2793
7.2761
7.2667
7.2637
7.2570
7.1683
7.1460
7.1253
7.0339
7.0253
6.9962
6.9874
6.9836
6.9749
5.6151
5.6068
5.5965
5.5864
5.5789
3.6983
3.6895
3.5359
3.5152
3.4918
3.4711
3.4645
3.4553
3.4203
3.4111
O
OH
S
F
2.00
1.17
2.01
2.00
1.00
0.98
2.14
f1 (ppm)

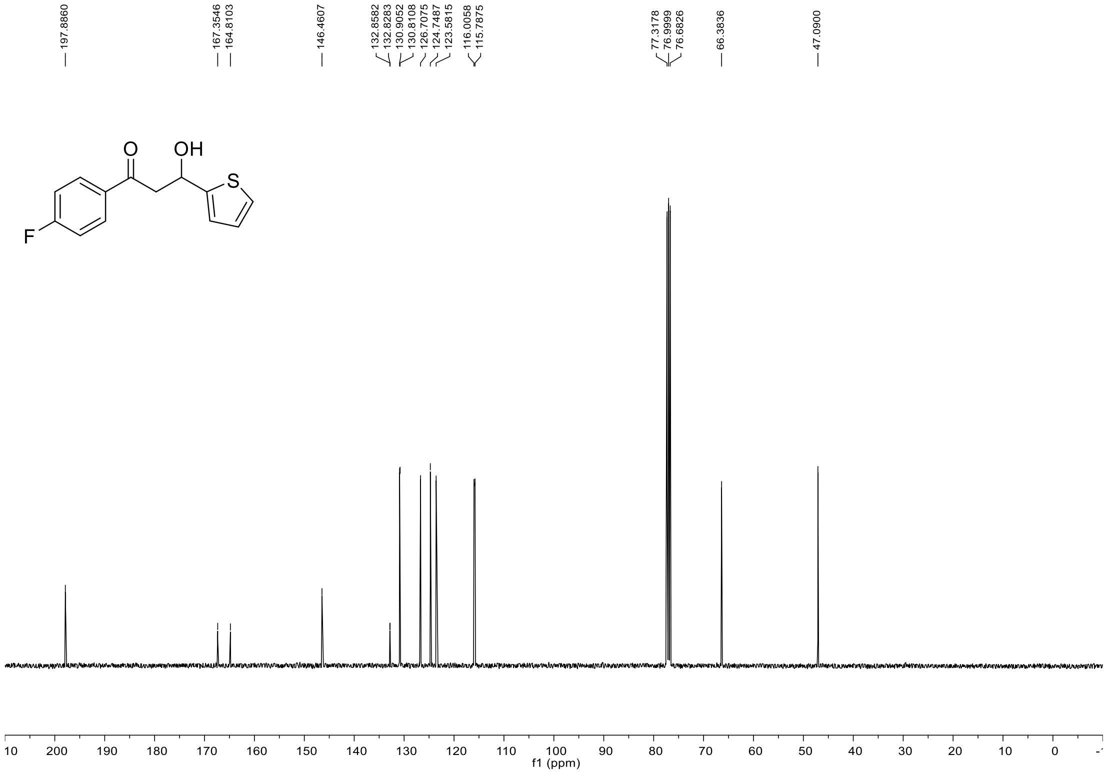
197.8860
167.3546
164.8103
146.4607
132.8582
132.8283
130.9052
130.8108
126.7075
124.7487
123.5815
116.0058
115.7875
77.3178
76.9999
76.6826
66.3836
47.0900
O
OH
S
F
f1 (ppm)

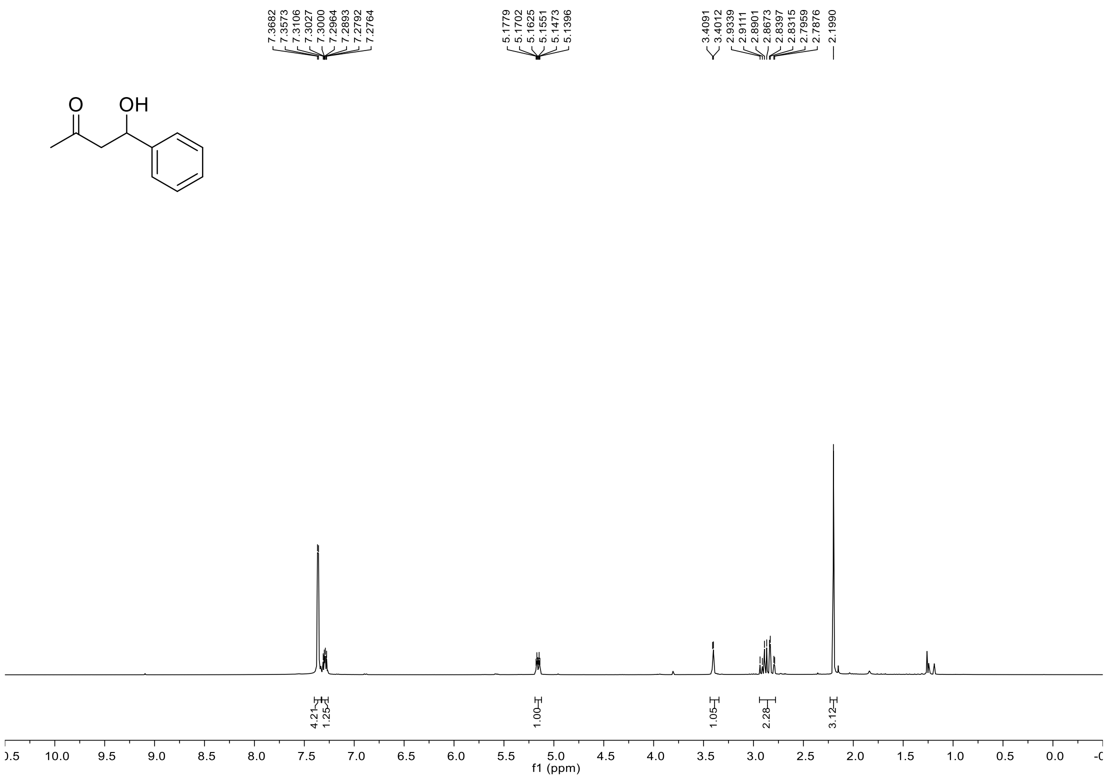
7.3682
7.3573
7.3106
7.3027
7.3000
7.2964
7.2893
7.2792
7.2764
5.1779
5.1702
5.1625
5.1551
5.1473
5.1396
3.4091
3.4012
2.9339
2.9111
2.8901
2.8673
2.8397
2.8315
2.7959
2.7876
2.1990
O
OH
4.21
1.25
1.00
1.05
2.28
3.12
f1 (ppm)

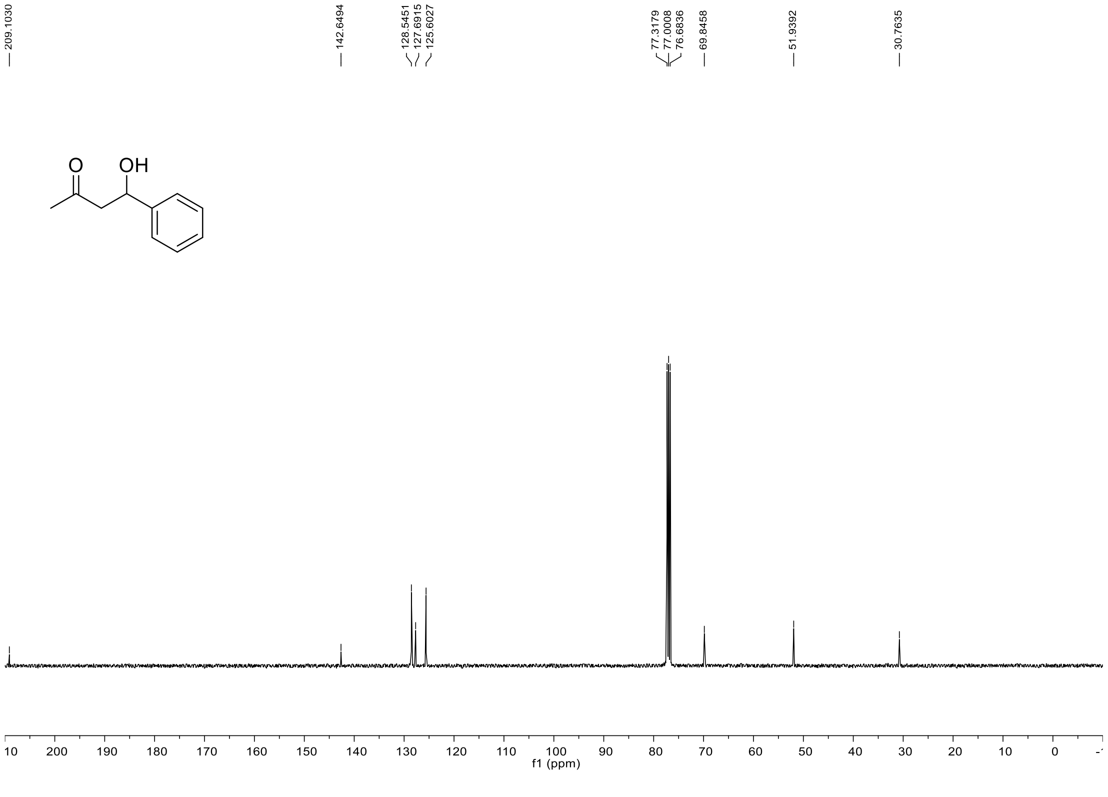
209.1030
142.6494
128.5451
127.6915
125.6027
77.3179
77.0008
76.6836
69.8458
51.9392
30.7635
O
OH
f1 (ppm)

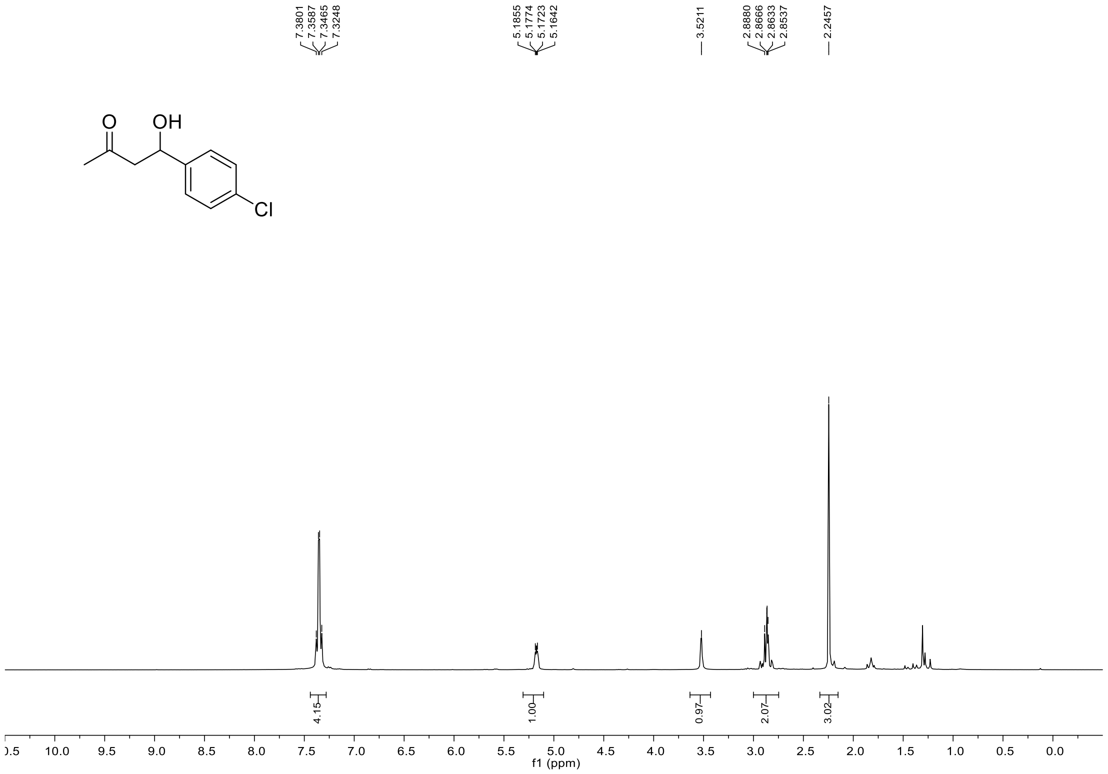
7.3801
7.3587
7.3465
7.3248
5.1855
5.1774
5.1723
5.1642
3.5211
2.8880
2.8666
2.8633
2.8537
2.2457
O
OH
Cl
4.15
1.00
0.97
2.07
3.02
f1 (ppm)

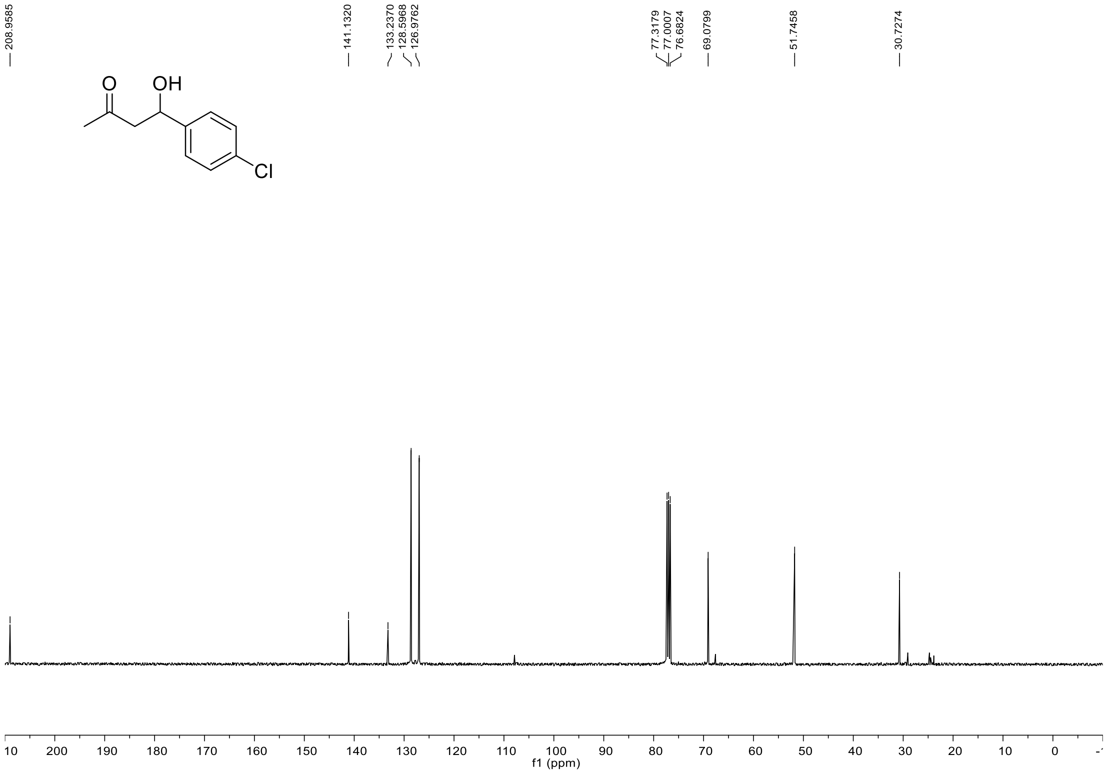
208.9585
141.1320
133.2370
128.5968
126.9762
77.3179
77.0007
76.6824
69.0799
51.7458
30.7274
O
OH
Cl
f1 (ppm)

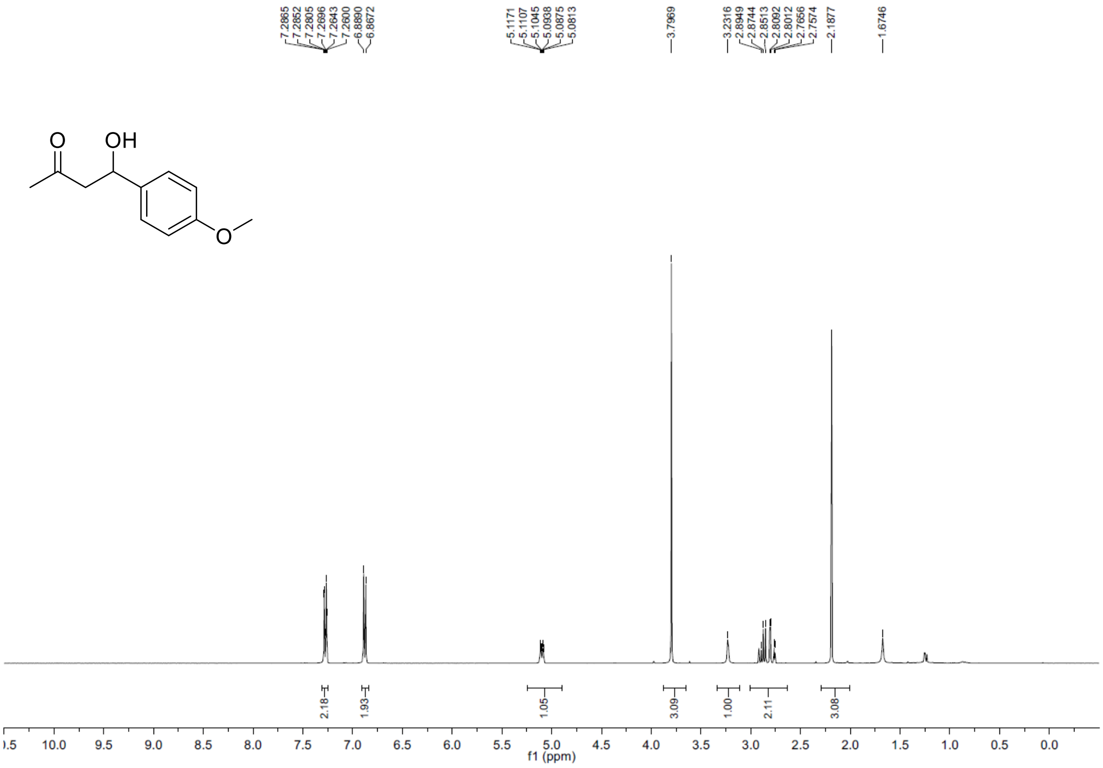

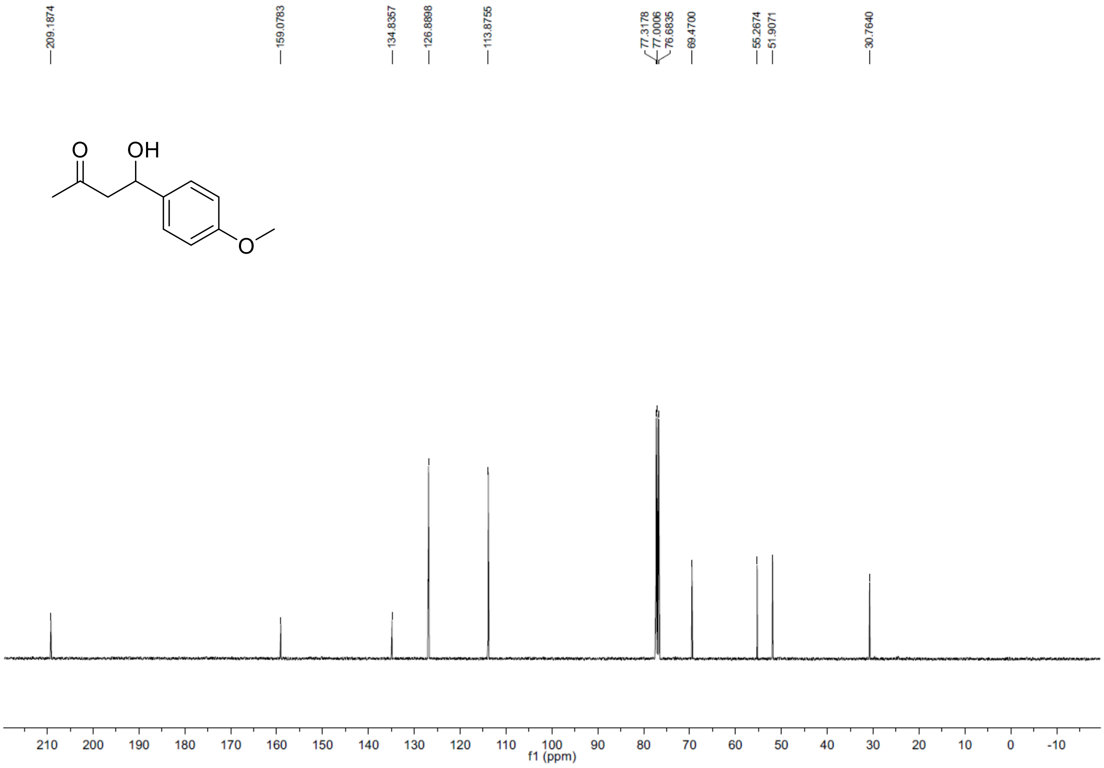

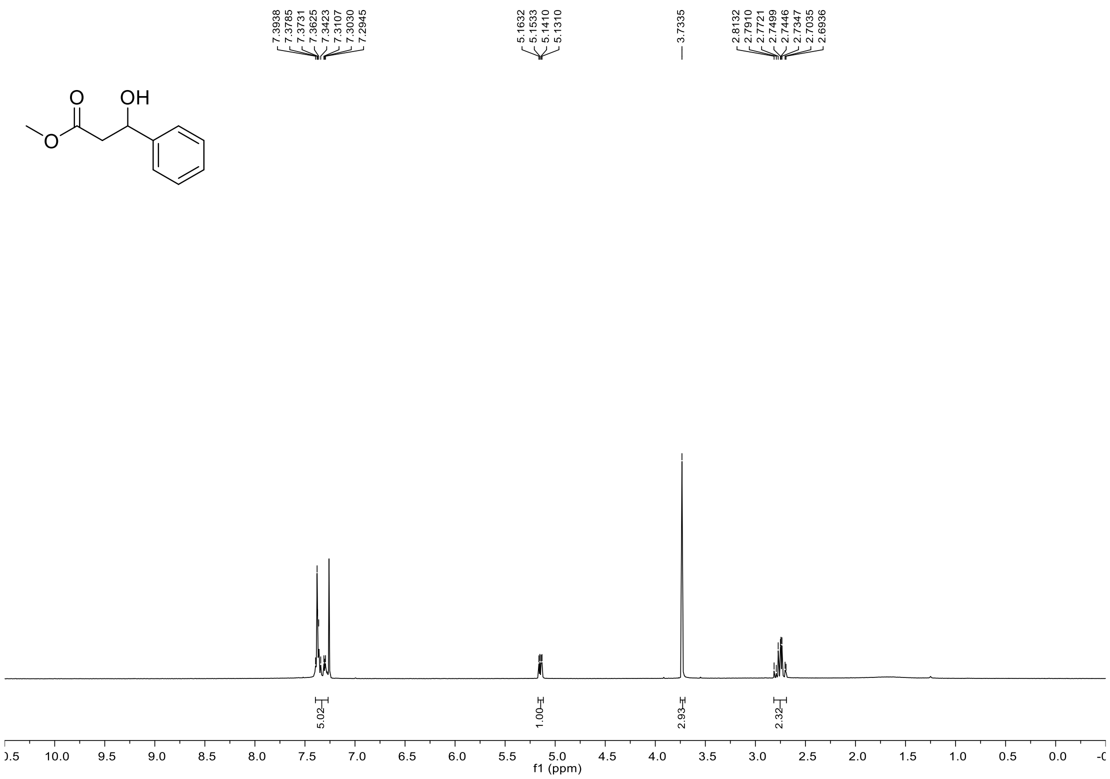
7.3938
7.3785
7.3731
7.3625
7.3423
7.3107
7.3030
7.2945
5.1632
5.1533
5.1410
5.1310
3.7335
2.8132
2.7910
2.7721
2.7499
2.7446
2.7347
2.7035
2.6936
O
OH
O
5.02
1.00
2.93
2.32
f1 (ppm)

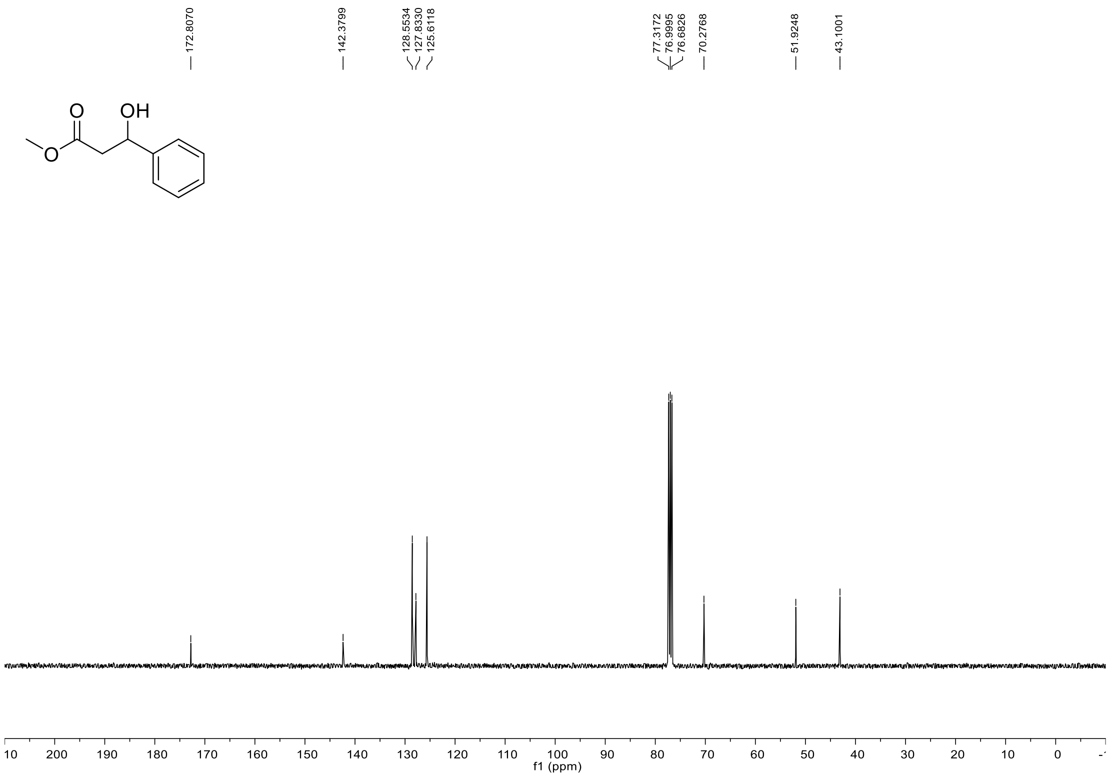
172.8070
142.3799
128.5534
127.8330
125.6118
77.3172
76.9995
76.6826
70.2768
51.9248
43.1001
O
OH
O
f1 (ppm)

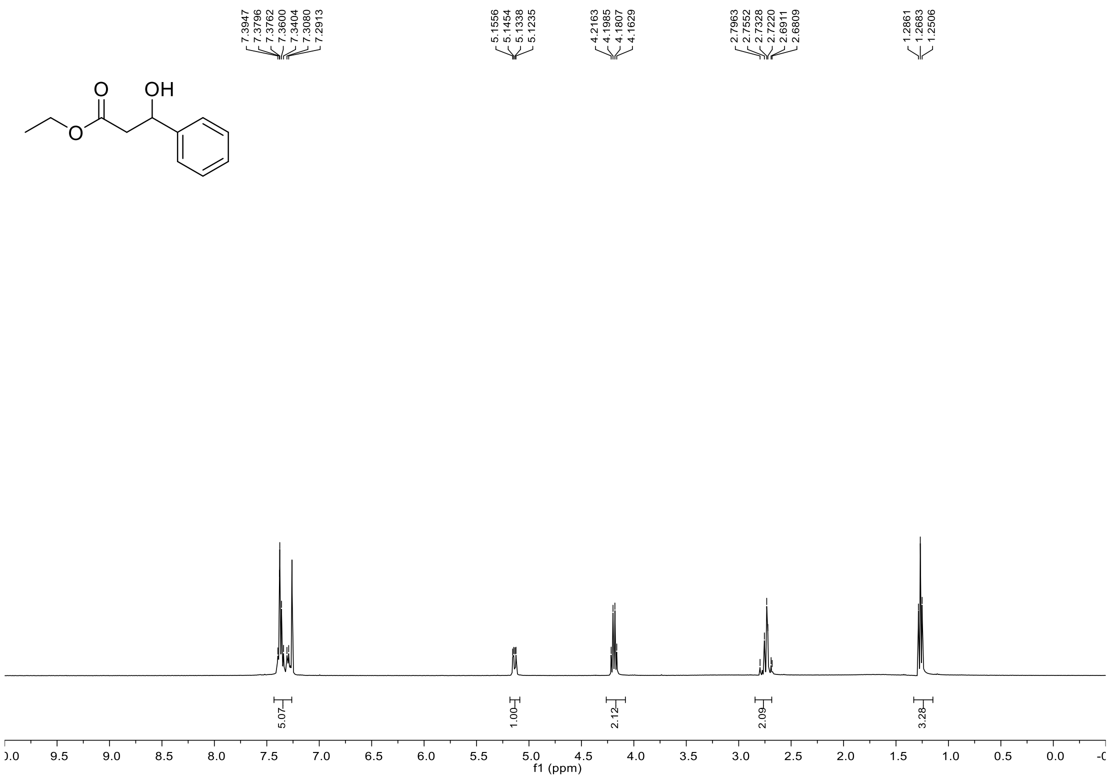
7.3947
7.3796
7.3762
7.3600
7.3404
7.3080
7.2913
5.1556
5.1454
5.1338
5.1235
4.2163
4.1985
4.1807
4.1629
2.7963
2.7552
2.7328
2.7220
2.6911
2.6809
1.2861
1.2683
1.2506
OH
O
O
5.07
1.00
2.12
2.09
3.28
f1 (ppm)

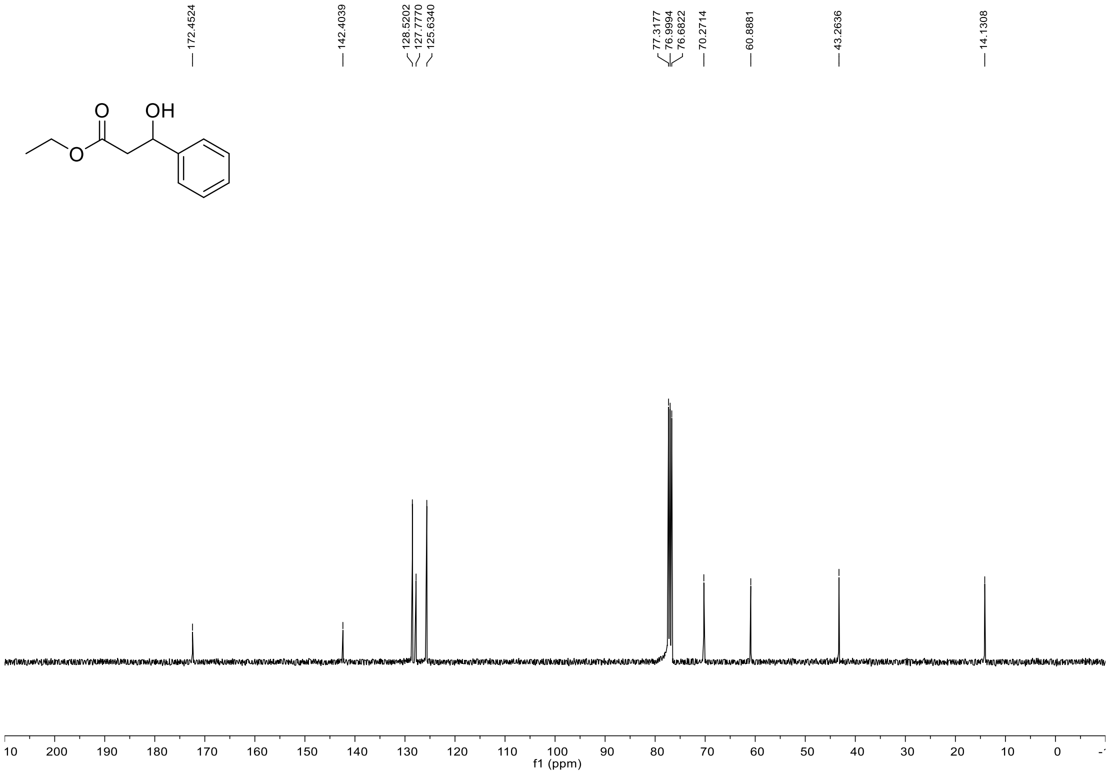
172.4524
142.4039
128.5202
127.7770
125.6340
77.3177
76.9994
76.6822
70.2714
60.8881
43.2636
14.1308
OH
O
O
f1 (ppm)

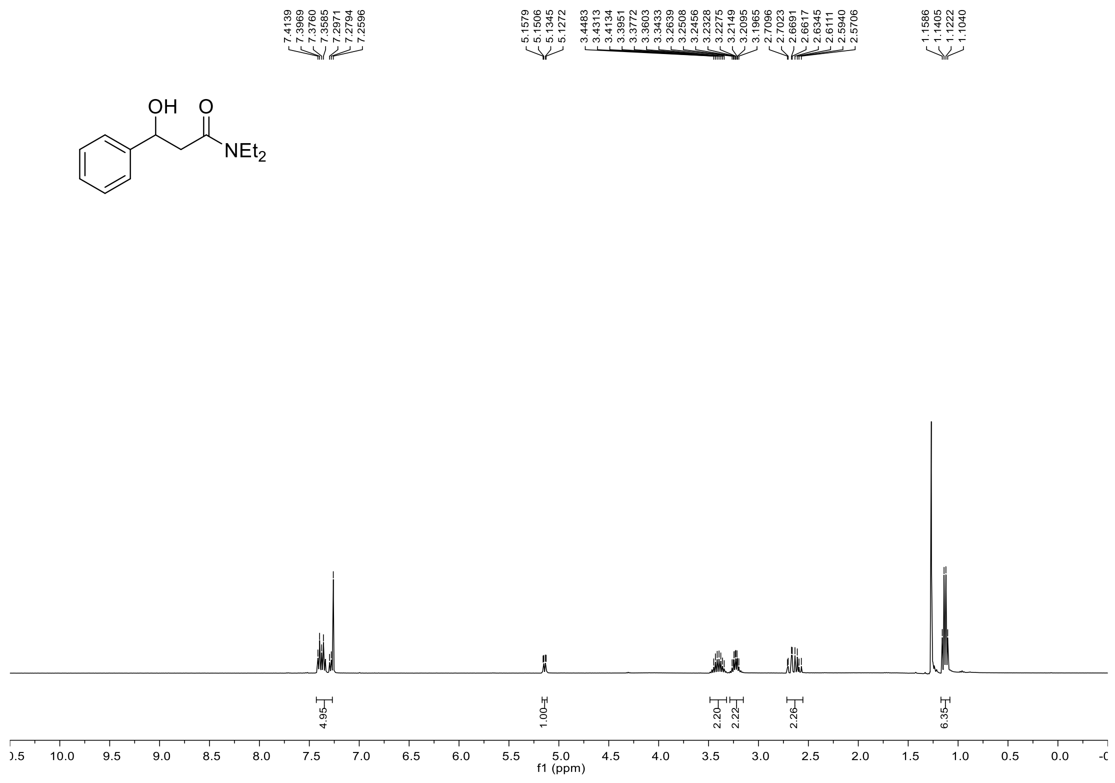
7.4139
7.3969
7.3760
7.3585
7.2971
7.2794
7.2596
5.1579
5.1506
5.1345
5.1272
3.4483
3.4313
3.4134
3.3951
3.3772
3.3603
3.3433
3.2639
3.2508
3.2456
3.2328
3.2275
3.2149
3.2095
3.1965
2.7096
2.7023
2.6691
2.6617
2.6345
2.6111
2.5940
2.5706
1.1586
1.1405
1.1222
1.1040
OH
O
NEt2
4.95
1.00
2.20
2.22
2.26
6.35
f1 (ppm)

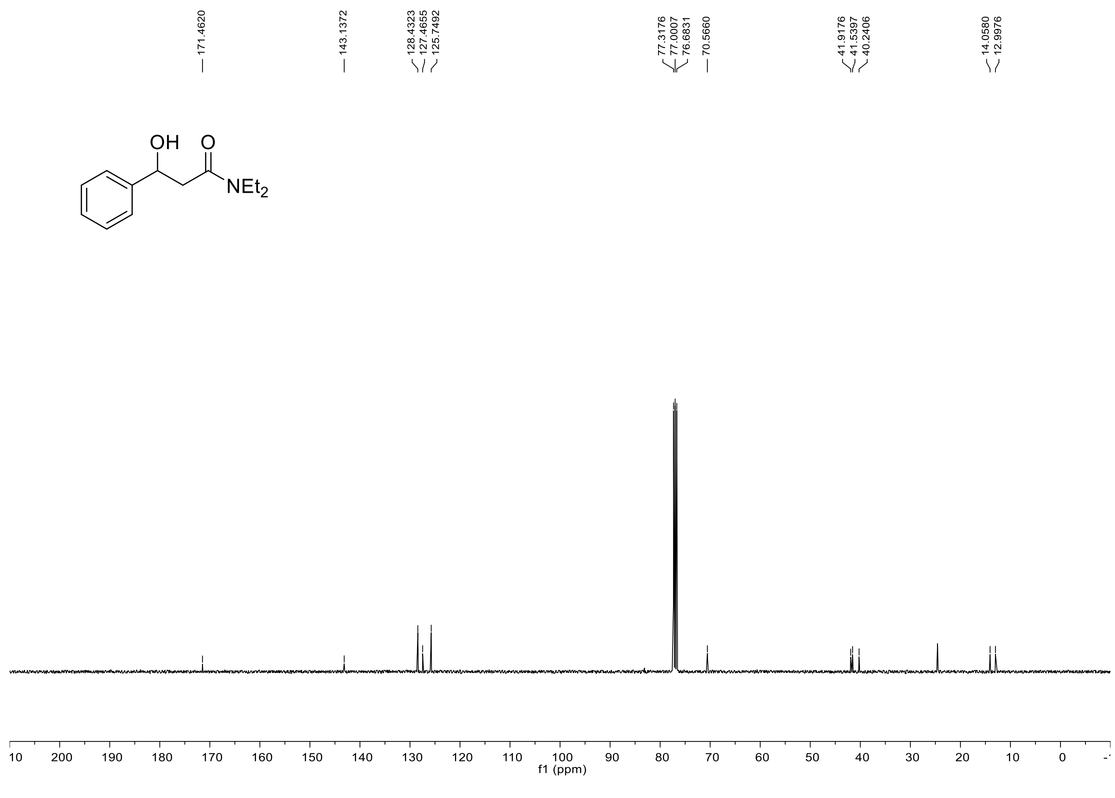
171.4620
143.1372
128.4323
127.4655
125.7492
77.3176
77.0007
76.6831
70.5660
41.9176
41.5397
40.2406
14.0580
12.9976
OH
O
NEt2
f1 (ppm)

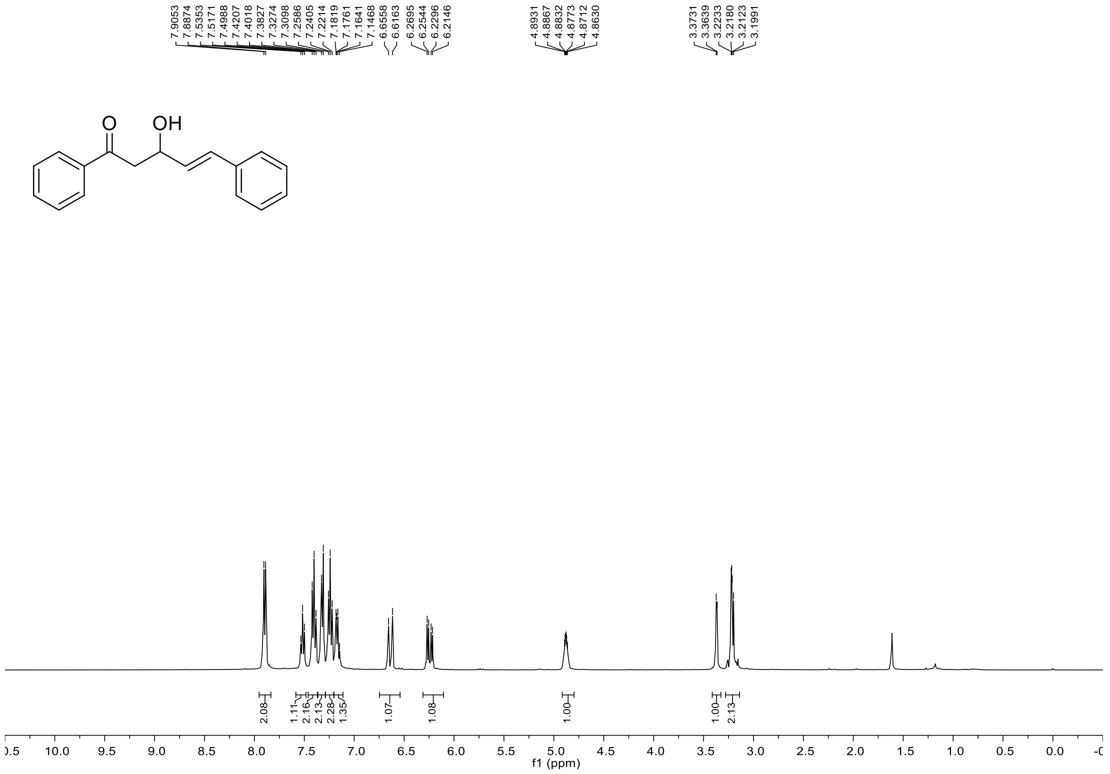
7.9053
7.8874
7.5353
7.5171
7.4988
7.4207
7.4018
7.3827
7.3274
7.3098
7.2586
7.2405
7.2214
7.1819
7.1761
7.1641
7.1468
6.6558
6.6163
6.2695
6.2544
6.2296
6.2146
4.8931
4.8867
4.8832
4.8773
4.8712
4.8630
3.3731
3.3639
3.2233
3.2180
3.2123
3.1991
O
OH
2.08
1.11
2.16
2.13
2.28
1.35
1.07
1.08
1.00
1.00
2.13
f1 (ppm)

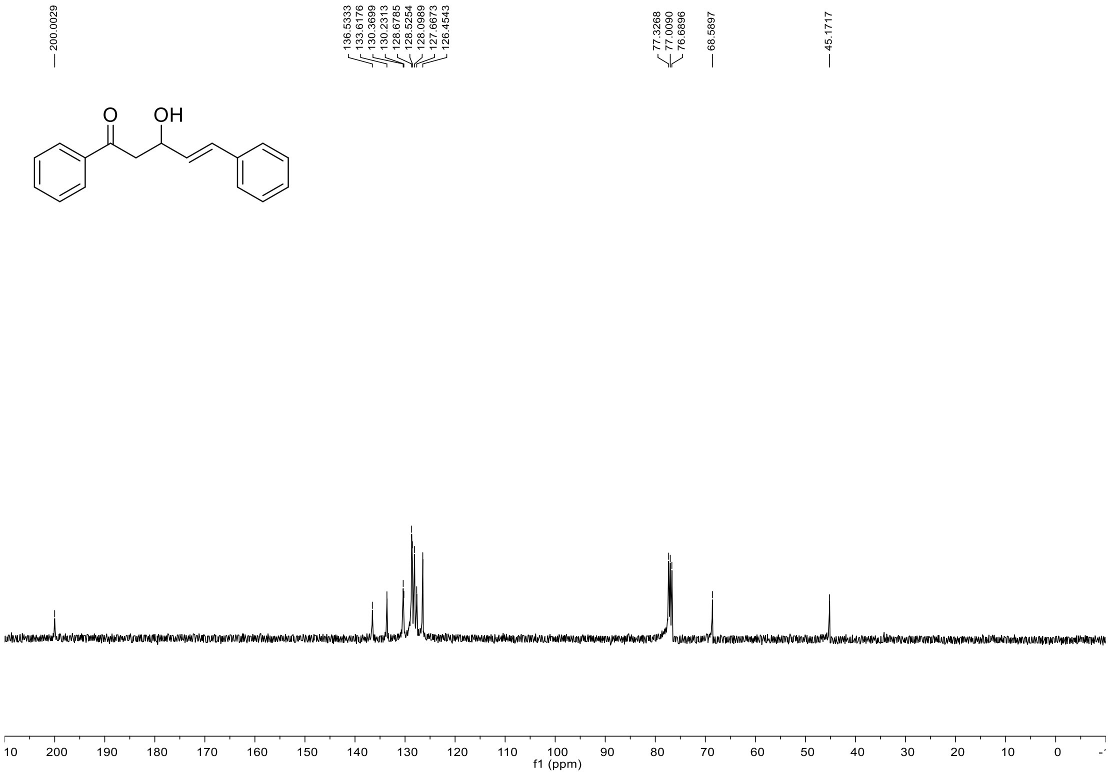
200.0029
136.5333
133.6176
130.3699
130.2313
128.6785
128.5254
128.0989
127.6673
126.4543
77.3268
77.0090
76.6896
68.5897
45.1717
O
OH
f1 (ppm)

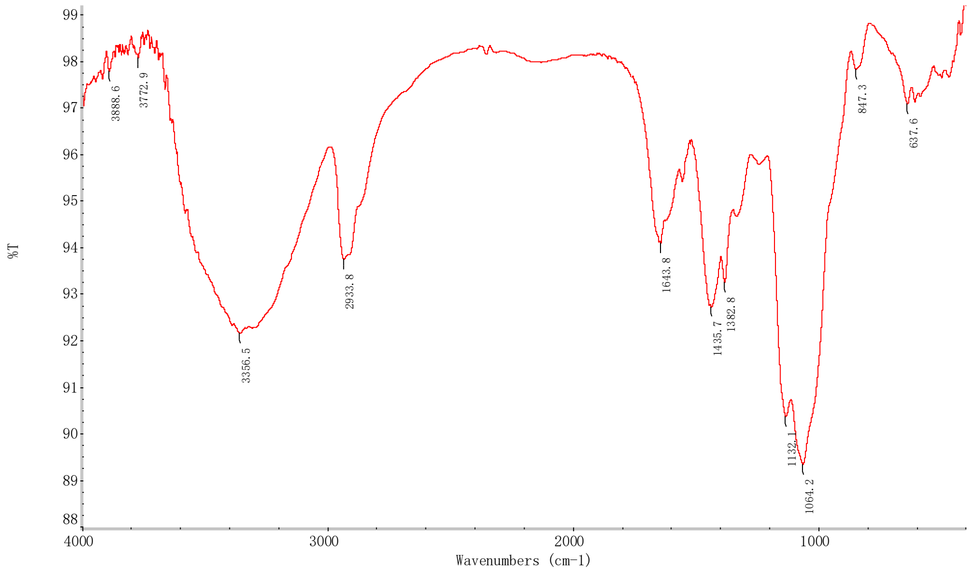

Figure S1. IR spectra of CP@Cu nanoparticles

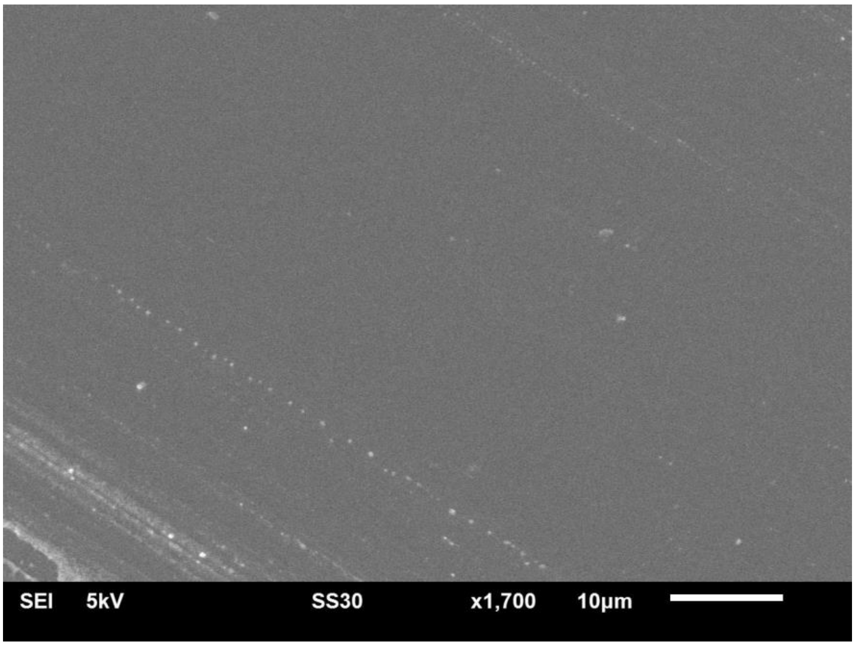

Figure S2. SEM image of CP@Cu nanoparticles
